# Digital Health Interventions in Pediatric Perioperative Care: A Network Meta-Analysis

**DOI:** 10.1001/jamapediatrics.2025.3099

**Published:** 2025-09-15

**Authors:** Ziyue Luo, Ruihao Zhou, Kailei Nong, Xiran Peng, Lu Chen, Peiyi Li, Sisi Deng, Mengchan Ou, Xuechao Hao, Ling Ye, Yaqiang Wang, Guo Chen, Sheyu Li, Tao Zhu

**Affiliations:** 1 Department of Anesthesiology, National Clinical Research Center for Geriatrics, West China Hospital, Sichuan University, Chengdu, China; 2 The Research Units of West China, Chinese Academy of Medical Sciences, West China Hospital, Sichuan University, Chengdu, China; 3 Department of Endocrinology and Metabolism, Laboratory of Diabetes and Metabolism Research, West China Hospital, Sichuan University, Chengdu, China; 4 Making GRADE the Irresistible Choice China Centre, Cochrane China Centre, Chinese Evidence-based Medicine, West China Hospital, Sichuan University, Chengdu, China; 5 Department of Pain Management, National Clinical Research Center for Geriatrics, West China Hospital, Sichuan University, Chengdu, China; 6 College of Software Engineering, Chengdu University of Information Technology, Chengdu, Sichuan, China

## Abstract

**Importance:**

Pediatric surgical patients often face considerable perioperative challenges, including anxiety and pain. Digital health interventions offer promise, but their efficacy remains uncertain.

**Objective:**

To compare the effects of digital health interventions in pediatric perioperative care.

**Data Sources:**

PubMed, Embase, Web of Science, CENTRAL, and CINAHL databases were searched up to March 1, 2025.

**Study Selection:**

Randomized clinical trials (RCTs) involving pediatric patients (aged ≤18 years) undergoing surgery with general anesthesia, where digital technology was used as a distraction intervention.

**Data Extraction and Synthesis:**

A frequentist network meta-analysis with random-effects model was used to calculate standardized mean differences (SMDs) or mean differences (MDs) with 95% CIs. Interventions were ranked using *P* values, risk of bias assessed using the Cochrane risk of bias tool 2, and certainty of evidence rated using the Grading of Recommendations, Assessment, Development, and Evaluations framework.

**Main Outcomes and Measures:**

Critical outcomes included pediatric preoperative anxiety, postoperative pain, emergence delirium, and induction compliance. Important but noncritical outcomes encompassed parental preoperative anxiety and postoperative satisfaction.

**Results:**

Of 7734 RCTs screened, 49 were included involving 4535 youth (pooled mean age, 7.42 years; 95% CI, 6.85 to 7.99; 2989 [65.9%] male) with 7 interventions: virtual reality (VR), 2-dimensional (2D) games, 2D videos, interactive robots, midazolam, control (standard care), and enhanced control (eg, booklets). Compared to control, VR (SMD, −1.14; 95% CI, −1.54 to −0.74; moderate certainty), 2D videos (SMD, −1.08; 95% CI, −1.51 to −0.65; moderate certainty), 2D games (SMD, −1.02; 95% CI, −1.54 to −0.49; low certainty), and enhanced control (SMD, −0.83; 95% CI, −1.53 to −0.13; low certainty) reduced preoperative anxiety. VR (SMD, −1.09; 95% CI, −1.58 to −0.59; moderate certainty), 2D games (SMD, −0.87; 95% CI, −1.62 to −0.12; low certainty), and 2D videos (SMD, −0.56; 95% CI, −1.06 to −0.06; moderate certainty) reduced postoperative pain. VR showed the greatest effect on compliance (MD, −0.93; 95% CI, −1.62 to −0.24; moderate certainty). No significant differences compared to control.

**Conclusions and Relevance:**

This network meta-analysis of RCTs found that VR, 2D videos, and 2D games significantly reduced pediatric perioperative anxiety and pain and improved induction compliance. These findings support the use of digital health interventions in pediatric perioperative care and their broader clinical adoption.

## Key Points

**Question:**

What are the comparative effects of digital health interventions across multiple perioperative outcomes (eg, anxiety, pain, delirium, and compliance) in youth undergoing surgery?

**Findings:**

In this network meta-analysis with 49 trials and 4535 youth, virtual reality (VR) was the most effective intervention for reducing preoperative anxiety and postoperative pain while improving anesthesia induction compliance; 2-dimensional (2D) videos and games showed moderate benefits. No digital intervention reduced postoperative delirium compared to standard care.

**Meaning:**

The findings suggest that VR should be prioritized as a first-line nonpharmacological intervention in pediatric perioperative care, with 2D–based tools serving as accessible alternatives when VR is unavailable.

## Teaser

This network meta-analysis evaluates the effects of digital health interventions in pediatric perioperative care.

## Introduction

Pediatric patients face considerable perioperative challenges, including preoperative anxiety, postoperative pain, emergence delirium, and compliance difficulties, which may negatively impact both immediate recovery and long-term health care experiences.^1,2,3^ Preoperative anxiety in youth—often stemming from fear of the unknown, anticipated pain, and separation from parents—peaks during induction of general anesthesia.^4^ This experience also profoundly impacts parents, influencing their anxiety levels and care satisfaction.^5^ Although midazolam remains commonly used for anxiety management, concerns about adverse effects and safety profiles have prompted exploration of nonpharmacological alternatives as potentially safer and more effective strategies in pediatric populations.^6,7,8,9^

Digital health interventions represent innovative nonpharmacological distraction techniques with demonstrated efficacy.^10^ These encompass virtual reality (VR), interactive 2-dimensional (2D) video games, educational videos, social robots, mobile health applications, and telemedicine services.^8^ VR’s immersive 3-dimensional environment create presence and full engagement, diverting attention from painful stimuli to reduce pain and anxiety.^11^ Interactive 2D modalities leverage youth’s digital familiarity to improve procedural compliance and preoperative preparedness.^12,13^ Emerging adjuncts, like social robots, improve induction cooperation through responsive interactions, supported by preliminary feasibility data.^14^ By addressing developmental needs while providing scalable, cost-effective solutions, these technologies constitute viable alternatives to pharmacologic approaches in contemporary perioperative care.^15^

Existing systematic reviews^11,12^ have typically focused on single modalities, such as virtual reality or video games, without comparing a broader range of digital health interventions. This study uses a network meta-analysis to systematically evaluate the effectiveness of diverse digital strategies in addressing key perioperative challenges, allowing for indirect comparisons and hierarchical ranking in the absence of head-to-head trials. This analysis provides comprehensive insights into the impact of digital health interventions on both youth and their families during the perioperative phase. By identifying effective and scalable digital strategies—particularly the superior performance of virtual reality—this study may help inform clinical decision-making and policy development in pediatric perioperative care.

## Methods

### Literature Search and Study Selection

This study followed the Preferred Reporting Items for Systematic Reviews and Meta-analyses (PRISMA) reporting guideline, and was registered in PROSPERO. We used a comprehensive search strategy, including medical subject headings and free text terms, to systematically search PubMed, Embase, Web of Science, CENTRAL, and CINAHL (via EBSCOhost) from inception to March 1, 2025. Key search terms included *child*,* perioperative period*,* surgery*, and* digital health interventions of interests* (eTable 1 in Supplement 1). We reviewed the reference lists of the included studies to supplement the eligible articles not found in the search.

We focused on youth (aged ≤18 years) undergoing surgery with general anesthesia who were treated by perioperative digital health interventions. These interventions were digital technologies, including VR, 2D videos, interactive games, and robots. The comparators were either standard care (no distraction), enhanced control (eg, educational materials), midazolam (a commonly used sedative with established anxiolytic effects), or an alternative active digital technology. Detailed definitions of interventions and comparators were presented in eTable 2 in Supplement 1. Eligible studies reported 1 of the following outcomes: pediatric preoperative anxiety, postoperative pain, emergence delirium, induction compliance, parental preoperative anxiety, and postoperative satisfaction. The definitions of outcome were listed in eTable 3 in Supplement 1. We included randomized clinical trials (RCTs) only. Studies were excluded if they involved nondigital interventions (eg, music, iPod, and radio) or multicomponent programs in which the specific effect of the digital component could not be isolated.

Two reviewers first independently screened studies based on the titles and abstracts and then screened the full texts. Any discrepancies between them prompted a thorough review of the discrepant articles. A senior reviewer was consulted to resolve the discrepancies if they could not reach the consensus.

### Data Extraction

Two reviewers independently extracted data from eligible articles, including the name of first author, publication year, countries, type of surgical procedures, baseline characteristics (eg, number of participants and age) in the intervention and control groups, timing of the digital intervention, and instruments and time points of outcome assessments. Self-reports were prioritized as the outcome measure for youth able to express their experiences, given their subjective reliability. When unavailable, caregiver or clinician observations were used as alternative assessments. Any discrepancy between reviewers were resolved through discussion.

### Risk of Bias Assessment

Pairwise reviewers assessed the risk of bias of the included RCTs using the Cochrane risk of bias tool 2. The tool includes the following 5 domains: (1) bias in the randomization process, (2) bias due to deviations from intended interventions, (3) bias from missing outcome data, (4) bias in outcome measurement, and (5) bias in selecting reported results. Each domain was rated as low risk, some concerns, or high risk. Overall bias was rated as low if all domains were low risk, as some concerns if no domain was high risk but some were concerns, and as high if any domain was high risk. Any discrepancy was resolved by discussion.

### Statistical Analysis

This study conducted a random-effects frequentist network meta-analysis using the graph-theoretical method available in the R package netmeta (version 3.1-1 [R Foundation]).^16^ We measured preoperative anxiety and postoperative pain using standardized mean differences (SMDs) and corresponding 95% CIs due to different scales or instruments across studies. We measured emergence delirium and induction compliance using mean differences (MDs) and 95% CIs due to the same scale across studies. Studies with missing outcome data were excluded from quantitative synthesis, and no imputation was performed. For better clinical interpretation, we converted SMDs back to the most commonly reported scales by multiplying them with the pooled standard deviation of the control groups in the included studies. These scales included the Modified Yale Preoperative Anxiety Scale for children’s anxiety, the Wong-Baker Faces Pain Rating Scale for children’s pain, the State-Trait Anxiety Inventory for parental anxiety, and the Verbal Score for parental satisfaction. Effect magnitudes were interpreted using Cohen criteria: small (0.2 to 0.5), medium (0.5 to 0.8), and large (>0.8). Effect sizes were interpreted using their 95% CIs to avoid overestimating clinical relevance. Large effects were considered as those where the entire CI exceeded 0.8. Statistical significance was defined by a 95% CI excluding 0.

We used forest plots and league tables to present network estimates, with interventions ranked by *P* values. Incoherence between direct and indirect comparisons was assessed using the netsplit function in the netmeta package (R software version 4.4.2), with a 2-tailed *P* value less than .05 considered significant.^17^ Transitivity was evaluated by comparing covariate distributions (eg, baseline anxiety and pain severity) across treatment comparisons. Heterogeneity (inconsistency) was assessed using the *I*^2^ statistic and Cochran *Q* test, with *P* less than .05 considered significant.^18^ We assessed publication bias using comparison-adjusted funnel plots and the Egger test for asymmetry in analyses with at least 10 studies.^19^ A tailed *P* value less than .10 in the Egger test was considered indicative of potential publication bias.

For preoperative anxiety and postoperative pain, we conducted a predefined subgroup analysis based on the type of surgery, categorized as day (outpatient) surgery or elective surgery. Additionally, sensitivity analyses were performed by excluding studies with a high risk of bias to assess the robustness of the findings.

### Certainty of Evidence

We followed the Grading of Recommendations, Assessment, Development, and Evaluation (GRADE) framework for network meta-analyses to rate the certainty of evidence for direct, indirect, and network estimates, categorizing evidence as high, moderate, low, or very low.^20^ Seven domains were considered: risk of bias, heterogeneity, indirectness, publication bias, intransitivity, incoherence, and imprecision.^21,22^

## Results

### Description of Included Studies

**Figure 1.**
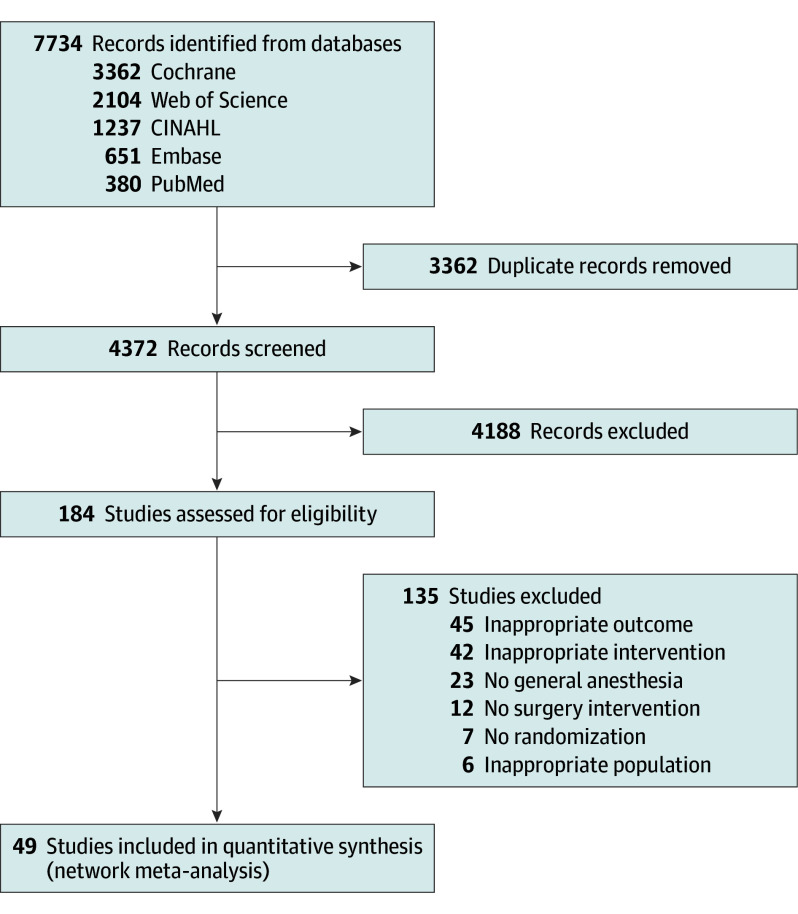
Study Flow Diagram

We screened 7734 records, reviewed 184 full-text articles, and finally included 49 RCTs (Figure 1; eTable 4 in Supplement 1) with 4535 youth (pooled mean age, 7.42 years; 95% CI, 6.85 to 7.99; 2989 [65.9%] male). Details of the identified RCTs are provided in eTable 5 in Supplement 1. The risk of bias assessment (eFigure 1 and eTable 6 in Supplement 1) showed that most studies (40 of 49 [81.6%]) had a low or concerned risk of bias. Nine studies were at high risk of bias: 4 for randomization issues, 4 for deviations from intended interventions, 2 for missing outcome data, and 1 for outcome measurement bias. Publication bias assessments are presented in eFigure 2 in Supplement 1 and indicated no evidence of publication bias for any outcome. Incoherence assessments of the network meta-analysis are summarized in eTable 7 in Supplement 1, heterogeneity assessments are shown in eTable 8 in Supplement 1, and the certainty of evidence for direct, indirect, and network estimates are provided in eTable 9 in Supplement 1.

### Critical Outcomes

**Figure 2.**
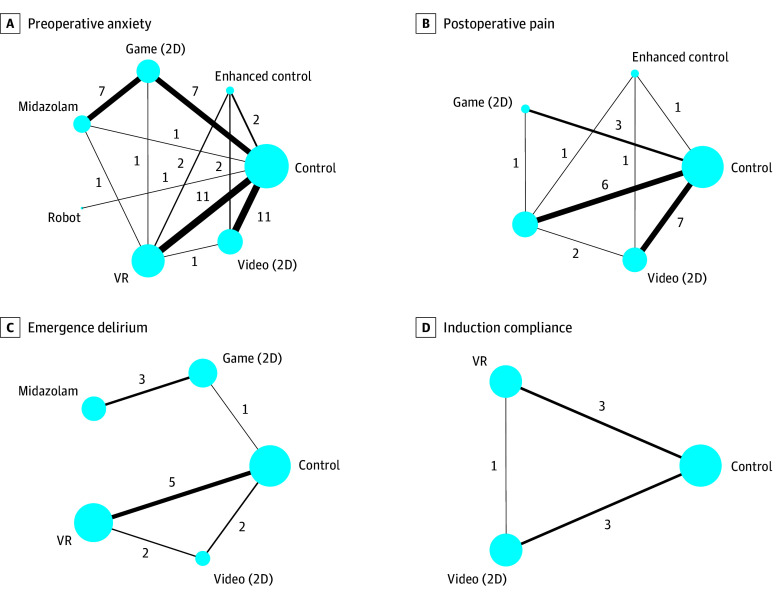
Digital Health Interventions in Pediatric Perioperative Care Each circular node represents a type of intervention. Circle size is proportional to the total number of patients. Connecting lines indicate direct comparisons of interventions, and their width is proportional to the number of pairwise comparisons. 2D indicates 2-dimensional; VR, virtual reality.

**Figure 3.**
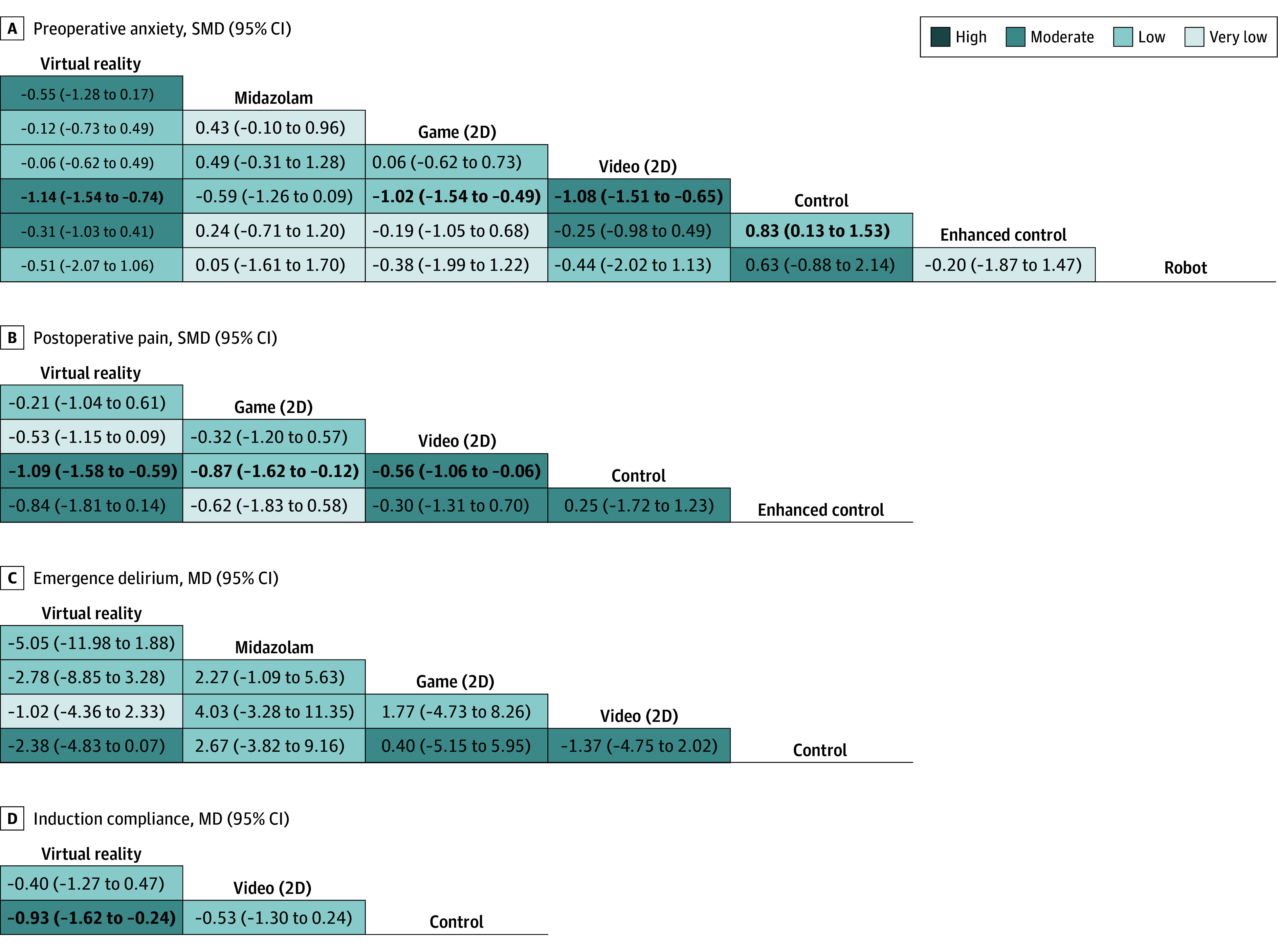
Preoperative Anxiety, Postoperative Pain, Emergence Delirium, and Induction Compliance in Youth The league tables present the absolute effects of each intervention compared to usual care (column treatment vs row treatment). Absolute effects are reported as standardized mean differences (SMDs) or mean differences (MDs) with 95% CIs. Bold text indicates statistical significance. Cell colors represent the certainty of evidence based on the Grading of Recommendations, Assessment, Development, and Evaluations (GRADE). 2D indicates 2-dimensional.

**Figure 4.**
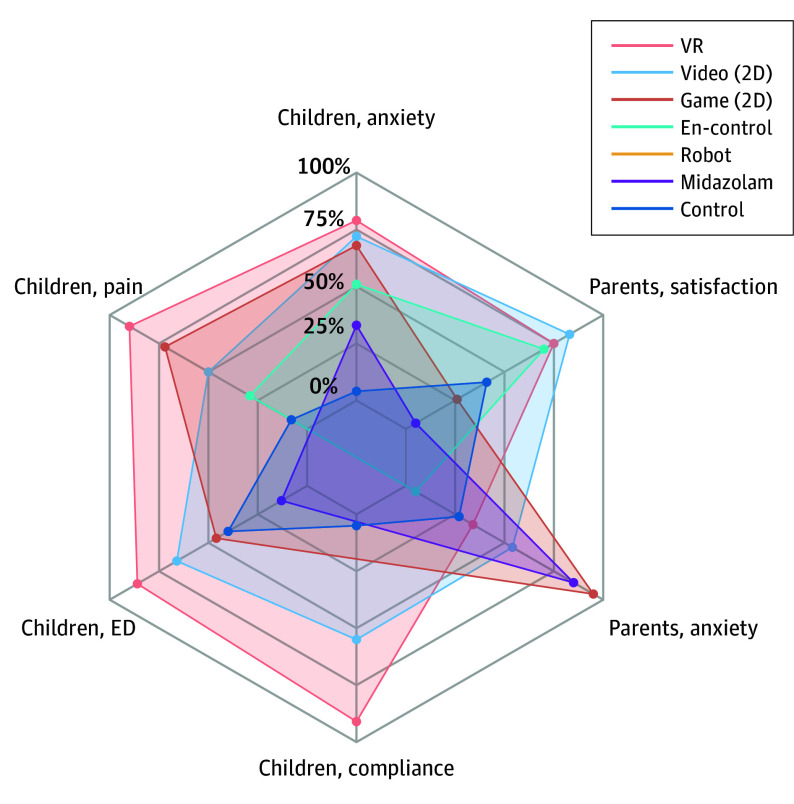
Interventions Based on *P* Values for Each Outcome The 6 angles of the radar plot represent different outcomes. Each point on the radar plot indicates the ranking probability, with points closer to the outer edge representing a higher-ranking probability. 2D indicates 2-dimentional; ED, emergence delirium; VR, virtual reality.

The Table summarizes the network meta-analysis findings across critical outcomes: preoperative anxiety, postoperative pain emergence delirium, and compliance during anesthesia induction. Figure 2 presents the network plots, with study volumes indicated: preoperative anxiety (43 studies), postoperative pain (18 studies), emergence delirium (11 studies), and compliance during anesthesia induction (7 studies). Figure 3 displays league tables of comparative effect estimates and GRADE certainty ratings for these outcomes. eFigure 3 in Supplement 1 presents forest plots summarizing all results. Figure 4 and eFigure 4 in Supplement 1 visualize the *P* values for all outcomes.

**Table.** Estimated Effect Size Results for Youth

Outcome	SMD or MD (95% CI)	SMD re-expressed on a common scale, MD (95% CI)^a^	*P* value	Certainty of evidence
**Youth, preoperative anxiety**				
Virtual reality	SMD, −1.14 (−1.54 to −0.74)	The Modified Yale Preoperative Anxiety scale (23 to 100): MD, −23.85 (−32.22 to −15.48)	.79	⊕⊕⊕⊝ Moderate^b^
Video (2D)	SMD, −1.08 (−1.51 to −0.65)	The Modified Yale Preoperative Anxiety scale (23 to 100): MD, −2.59 (−31.59 to −13.60)	.72	⊕⊕⊕⊝ Moderate^b^
Game (2D)	SMD, −1.02 (−1.54 to −0.49)	The Modified Yale Preoperative Anxiety scale (23 to 100): MD, −21.34 (−32.22 to −10.25)	.68	⊕⊕⊝⊝ Low^b^^,^^c^
Enhanced control	SMD, −0.83 (−1.53 to −0.13)	The Modified Yale Preoperative Anxiety scale (23 to 100): MD, −17.36 (−32.01 to −2.72)	.51	⊕⊕⊝⊝ Low^b^^,^^d^
An interactive robot	SMD, −0.63 (−2.14 to 0.88)	The Modified Yale Preoperative Anxiety scale (23 to 100): MD, −13.18 (−44.77 to 18.41)	.43	⊕⊕⊕⊝ Moderate^e^
Midazolam	SMD, −0.59 (−1.26 to 0.09)	The Modified Yale Preoperative Anxiety scale (23 to 100): MD, −12.34 (−26.36 to 1.88)	.33	⊕⊕⊝⊝ Low^c^^,^^e^
Control	NA	NA	.04	NA
**Youth, postoperative pain**				
Virtual reality	SMD, −1.09 (−1.58 to −0.59)	Wong-Baker Faces Pain Rating scale (0 to 10): MD, −1.84 (−2.67 to −1.00)	.90	⊕⊕⊕⊝ Moderate^b^
Game (2D)	SMD, −0.87 (−1.62 to −0.12)	Wong-Baker Faces Pain Rating scale (0 to 10): MD, −1.47 (−2.74 to −0.20)	.72	⊕⊕⊝⊝ Low^b^^,^^c^
Video (2D)	SMD, −0.56 (−1.06 to −0.06)	Wong-Baker Faces Pain Rating scale (0 to 10): MD, −0.95 (−1.79 to −0.10)	.50	⊕⊕⊕⊝ Moderate^b^
Enhanced control	SMD, −0.25 (−1.23 to 0.72)	Wong-Baker Faces Pain Rating scale (0 to 10): MD, −0.42 (−2.08 to 1.22)	.29	⊕⊕⊕⊝ Moderate^e^
Control	NA	NA	.08	NA
**Youth, emergence delirium**				
Virtual reality	MD, −2.38 (−4.83 to 0.07)	NA	.86	⊕⊕⊝⊝ Low^b^^,^^e^
Video (2D)	MD, −1.37 (−4.75 to 2.02)	NA	.66	⊕⊕⊕⊝ Moderate^e^
Control	NA	NA	.46	NA
Game (2D)	MD, 0.40 (−5.15 to 5.95)	NA	.40	⊕⊕⊕⊝ Moderate^e^
Midazolam	MD, 2.67 (−3.82 to 9.16)	NA	.13	⊕⊕⊝⊝ Low^e^^,^^f^
**Youth, induction compliance**				
Virtual reality	MD, −0.93 (−1.62 to −0.24)	NA	.91	⊕⊕⊕⊝ Moderate^b^
Video (2D)	MD, −0.53 (−1.30 to 0.24)	NA	.55	⊕⊕⊝⊝ Low^b^^,^^e^
Control	NA	NA	.05	NA

Abbreviations: 2D, 2-dimensional; GRADE, the grading of recommendations assessment, development, and evaluation; MD, mean difference; NA, not applicable; SMD, standardized mean difference.

^a^ MDs should be interpreted with caution because the results are based on the pooled control group SD for a subset of studies in the analysis that used the most commonly reported scale.

^b^ Downgraded by 1 level due to concerns about heterogeneity of effect estimates across trials.

^c^ Downgraded by 1 level due to concerns about methodological considerations, including a lack of blinding and, in some cases, other sources of bias.

^d^ Downgraded by 1 level due to incoherence, based on the difference between direct and indirect estimates, their 95% confidence intervals, and the *P* value for inconsistency.

^e^ Downgraded by 1 level due to concerns about imprecision; the confidence interval suggests the possibility of a null effect or benefit for either intervention.

^f^ Downgraded by 1 level due to concerns about the indirect certainty being derived from other direct comparison evidence, which is of moderate quality.

Compared to control group, VR (SMD, −1.14; 95% CI, −1.54 to −0.74; moderate certainty), 2D videos (SMD, −1.08; 95% CI, −1.51 to −0.65; moderate certainty), 2D games (SMD, −1.02; 95% CI, −1.54 to −0.49; low certainty), and enhanced control (SMD, −0.83; 95% CI, −1.53 to −0.13; low certainty) showed statistically significant reductions in pediatric preoperative anxiety (Figure 3). Based on Cohen criteria, VR, 2D videos, and 2D games showed moderate to large effects, while enhanced control showed a small to large effect. SMDs were recalibrated using a pooled SD of 20.92 points on the Modified Yale Preoperative Anxiety Scale (range, 23-100). For example, VR’s SMD, of −1.14 corresponds to a 23.85-point reduction (95% CI, −32.22 to −15.48) on this scale, indicating a clinically meaningful improvement in preoperative anxiety levels.

Compared to control group, VR demonstrated a moderate to large effect (SMD, −1.09; 95% CI, −1.58 to −0.59; moderate certainty), equivalent to a 1.84-point reduction (95% CI, −2.67 to −1.00) on the Wong-Baker Faces Pain Rating Scale (range, 0-10). 2D games (SMD, −0.87; 95% CI, −1.62 to −0.12; low certainty) and 2D videos (SMD, −0.56; 95% CI, −1.06 to −0.06; moderate certainty) showed small to large effects, with wide 95% CIs spanning minimal to substantial effects. VR was the most effective intervention for improving pediatric compliance during anesthesia induction (MD, −0.93; 95% CI, −1.62 to −0.24; moderate certainty). None of the interventions showed a significant effect on postoperative emergence delirium. Although virtual reality ranked highest among interventions, it did not demonstrate a statistically significant benefit (MD, −2.38; 95% CI, −4.83 to 0.07; low certainty) (Figure 3; eFigure 3 in Supplement 1).

### Important But Noncritical Outcomes

eTable 10 in Supplement 1 summarizes the findings for important but noncritical outcomes: parental preoperative anxiety (11 studies) and postoperative satisfaction (10 studies). Their network plots are shown in eFigure 5 in Supplement 1 and league tables are presented in eFigure 6 in Supplement 1.

Regarding parental preoperative anxiety, 2D games (SMD, −1.59; 95% CI, −2.12 to −1.06; moderate certainty) and midazolam (SMD, −1.46; 95% CI, −2.10 to −0.82; moderate certainty) indicated a large effect in reducing anxiety levels compared to the control group. In contrast, VR showed no statistically significant effect (SMD, −0.03; 95% CI, −0.21 to 0.15; moderate certainty; eTable 9 in Supplement 1). On the State-Trait Anxiety Inventory (range, 20-80), 2D games resulted in an 11.0-point reduction in parental preoperative anxiety (95% CI, −14.67 to −7.34), indicating a clinically meaningful improvement. No statistically significant differences were observed among the interventions in parental satisfaction compared to the control group. Although 2D video ranked highest, it did not demonstrate a statistically significant benefit (SMD, 0.43; 95% CI, −0.08 to 0.95; moderate certainty).

### Subgroup and Sensitivity Analyses

To evaluate anxiety management strategies across clinical settings, we conducted subgroup analyses comparing pediatric patients undergoing day/outpatient procedures vs elective inpatient surgeries. For preoperative anxiety in outpatient surgery, 2D video showed the strongest effect (SMD, −1.45, 95% CI, −2.02 to −0.88). In elective inpatient surgery, multiple interventions were effective: 2D games (SMD, −1.30, 95% CI, −1.93 to −0.67), VR (SMD, −1.28, 95% CI, −1.70 to −0.86), enhanced control (SMD, −1.30, 95% CI, −2.11 to −0.50), midazolam (SMD, −0.97, 95% CI, −1.87 to −0.06), and 2D video (SMD, −0.78, CI −1.28 to −0.29). These results suggest 2D video is optimal for outpatient settings, while 2D games and VR may be selected for elective inpatient cases (eFigures 7-9 in Supplement 1).

For reducing postoperative pain in youth undergoing day or outpatient surgery, 2D video showed significant efficacy (SMD, −0.74, 95% CI, −1.31 to −0.17; eFigure 10 in Supplement 1). In elective surgery, VR (SMD, −1.28, 95% CI, −1.78 to −0.77) and 2D games (SMD, −1.10, 95% CI, −1.90 to −0.30) were statistically effective (eFigure 11 in Supplement 1). Thus, 2D videos are likely the most suitable option for pain reduction in day or outpatient surgery, whereas VR and 2D games appear to be the most effective options for pain reduction in elective surgery (eFigure 12 in Supplement 1). Sensitivity analysis excluding studies with high risk of bias (n = 9) yielded results consistent with the primary analysis (eFigure 13 in Supplement 1).

## Discussion

This study presents the first network meta-analysis comparing digital health interventions for reducing preoperative anxiety, postoperative pain, and emergence delirium while improving compliance in youth undergoing elective surgery, concurrently evaluating parental preoperative anxiety and postoperative satisfaction. Consistent with previous studies,^23^ our findings on critical outcomes revealed that digital health interventions (VR, 2D videos, and 2D games) significantly reduced preoperative anxiety and postoperative pain vs control group (standard care). Notably, compared to midazolam—the pharmacological benchmark for preoperative anxiety management—digital interventions achieved comparable efficacy in anxiety reduction without drug-related adverse effects.^24^ This finding challenges pharmacology’s dominance in pediatric preoperative care, supporting integration of nonpharmacological alternatives.

Our analysis detected no statistically significant differences in critical outcomes between VR, 2D games, and 2D videos. However, subgroup analysis indicated 2D videos were most effective in reducing preoperative anxiety and postoperative pain in day/outpatient surgical settings, potentially attributable to their accessibility and ease of deployment in time-constrained settings. Unlike VR, which requires specialized equipment, 2D videos offer immediate engagement with minimal logistical demands.^25^ While this practical advantage supports their adoption in outpatient care, such recommendations may be driven more by resource availability than by true differences in efficacy—and their applicability may vary depending on institutional context. For preoperative anxiety and postoperative pain management in elective surgeries, VR and 2D games demonstrated potentially stronger effects. VR provides immersive multisensory input that diverts focus from noxious stimuli,^26,27,28,29,30^ while 2D games promote cognitive distraction and emotional regulation, particularly benefiting youth with anticipatory anxiety.^31^

Additionally, VR demonstrated superior effectiveness in improving youth’s compliance compared to the control group, although findings across seven randomized trials were inconsistent,^3,32,33,34,35,36^ likely due to heterogeneity in compliance assessments and variations in VR exposure duration. No significant differences in emergence delirium were observed among digital interventions; however, midazolam appeared to increase its risk, raising safety concerns.^37^ While these findings may support digital strategies over pharmacologic options, interpretation is limited by the small number of studies, modest sample sizes, and inconsistent outcome definitions and measurement tools. These limitations highlight the need for future multicenter studies with larger cohorts and standardized outcome measures.

Analysis of important but noncritical outcomes revealed both 2D games and midazolam reduced parental preoperative anxiety via distinct pathways, with moderate certainty of evidence. When comparing digital and pharmacologic interventions, it is important to consider both the magnitude and certainty of effects. Interactive 2D games achieved anxiety reduction through child engagement in goal-oriented play (eg, reduction in observable distress behaviors like crying), with parent-child co-play amplifying effects through shared behavioral control.^38^ In contrast, the immersive nature of VR may reduce parental involvement, potentially limiting its psychosocial benefits for caregivers. Midazolam, through its rapid sedative effects (eg, drowsiness), provided immediate reassurance via observable physiological changes.^39,40^ This reflects a decision-making bias wherein caregivers prioritized immediate pharmacological certainty over behavioral modalities’ delayed cognitive benefits.^41,42,43^ Parental postoperative satisfaction was highest with 2D videos, correlating with their efficacy in reducing youth’s preoperative anxiety during day surgeries or outpatient procedures. Their simplicity, immediacy, and low-risk, low-cost design align with parental expectations and enhance perioperative confidence.

### Limitations

Despite the demonstrated efficacy of digital health interventions in pediatric perioperative care, our study has several limitations. First, although we conducted a comprehensive review and network meta-analysis, heterogeneity across studies—particularly in control conditions and limited reporting of routine care—may have affected the consistency of results. Second, unmeasured factors, such as developmental stage, preintervention preparation quality, and parent-child interactions, could have influenced intervention effectiveness, warranting further exploration. Third, risks of selection and attrition bias, along with sociocultural differences, health care disparities, and uneven age representation, may limit the robustness and generalizability of the findings. Fourth, digital health classifications may differ depending on use context.^44^ Additionally, practical implementation faces challenges, such as resource limitations (eg, technological infrastructure deficits, staff training gaps); digital divide, where unequal access to technology may hinder equitable adoption, requiring lower-cost alternatives and scalable delivery strategies; patient or family acceptability disparities requiring culturally adapted education frameworks; and heterogeneous outcome metrics necessitating standardized patient-reported outcome measures for postoperative recovery monitoring.^8^

Future research should prioritize age-specific protocols (particularly for toddlers) and pain severity stratification, given the paucity of data on acute pain management in youth. Optimizing VR timing (eg, preinduction vs postoperative) and duration requires systematic evaluation to maximize therapeutic windows.^45^ Collaborations with developers could enhance efficacy through pediatric-tailored VR applications integrating developmental psychology principles.^46^ Emerging digital tools show promise in predicting postoperative pain via machine learning algorithms analyzing preoperative biomarkers and behavioral data, though current evidence remains preliminary.^47,48^ Multisite RCTs with standardized end points are critical to validate these interventions and establish clinical integration pathways.

## Conclusions

In this study, digital health interventions, including VR, 2D videos, and 2D games, demonstrate efficacy in reducing pediatric perioperative anxiety and pain, enhancing induction compliance, and reducing parental anxiety. Despite proven benefits, persistent challenges include study heterogeneity and implementation barriers. Future research should prioritize standardized outcome measures and technology optimization for real-world clinical adoption to enhance the perioperative experience for pediatric patients and caregivers.

## Notes

Supplement 1. eTable 1. The search strategies used in PubMed, Embase, CENTRAL, WOS and CINAHL eTable 2. Summary of included interventions eTable 3. Definition of outcomes eTable 4. References of trials included in the network meta-analysis eTable 5. Characteristics of the included randomized controlled trials eTable 6. Assessment of risk of bias using the Cochrane Risk of Bias Tool eTable 7. Incoherence assessments eTable 8. Heterogeneity (Inconsistency) assessments eTable 9. Certainty of evidence for direct, indirect and network estimates eTable 10. Estimated effect size results for parents eFigure 1. Assessment of the risk of bias in all included studies eFigure 2. Comparison-adjusted funnel plot in relation to the network meta-analysis of primary outcomes eFigure 3. Forest plots eFigure 4. Radar Plots of Interventions Based on P-Scores for Each Outcome eFigure 5. Network Plots of Digital Health Interventions for Secondary Outcomes eFigure 6. League Tables of Parental Preoperative Anxiety and Postoperative Satisfaction eFigure 7. Subgroup Analysis: Network Meta-Analysis of Anxiety in Children Undergoing Day or Outpatient Surgery eFigure 8. Subgroup Analysis: Network Meta-Analysis of Anxiety in Children Undergoing Elective Surgery eFigure 9. Subgroup Analysis: P-Score Comparison of Digital Health Interventions for Reducing Children's Anxiety Across Surgery Types (Day/Outpatient vs. Elective Surgery) eFigure 10. Subgroup Analysis: Network Meta-Analysis of Pain in Children Undergoing Day or Outpatient Surgery eFigure 11. Subgroup Analysis: Network Meta-Analysis of Pain in Children Undergoing Elective Surgery eFigure 12. Subgroup Analysis: P-Score Comparison of Digital Health Interventions for Reducing Children's Pain Across Surgery Types (Day/Outpatient vs. Elective Surgery) eFigure 13. Sensitivity analysis: Exclusion of Studies with High Risk of Bias

Supplement 2. Data sharing statement
