# Supplementary material for: Digital Health Interventions in Pediatric Perioperative Care: A Network Meta-Analysis
Source: JAMA Pediatr. 2025 Sep 15;179(11):1153–61. doi: 10.1001/jamapediatrics.2025.3099 (PMC12439185; doi:10.1001/jamapediatrics.2025.3099)
Supplement: Supplement 1. — eTable 1. The search strategies used in PubMed, Embase, CENTRAL, WOS and CINAHL eTable 2. Summary of included interventions eTable 3. Definition of outcomes eTable 4. References of trials included in the network meta-analysis eTable 5. Characteristics of the included randomized controlled trials eTable 6. Assessment of risk of bias using the Cochrane Risk of Bias Tool eTable 7. Incoherence assessments eTable 8. Heterogeneity (Inconsistency) assessments eTable 9. Certainty of evidence for direct, indirect and network estimates eTable 10. Estimated effect size results for parents eFigure 1. Assessment of the risk of bias in all included studies eFigure 2. Comparison-adjusted funnel plot in relation to the network meta-analysis of primary outcomes eFigure 3. Forest plots eFigure 4. Radar Plots of Interventions Based on P-Scores for Each Outcome eFigure 5. Network Plots of Digital Health Interventions for Secondary Outcomes eFigure 6. League Tables of Parental Preoperative Anxiety and Postoperative Satisfaction eFigure 7. Subgroup Analysis: Network Meta-Analysis of Anxiety in Children Undergoing Day or Outpatient Surgery eFigure 8. Subgroup Analysis: Network Meta-Analysis of Anxiety in Children Undergoing Elective Surgery eFigure 9. Subgroup Analysis: P-Score Comparison of Digital Health Interventions for Reducing Children's Anxiety Across Surgery Types (Day/Outpatient vs. Elective Surgery) eFigure 10. Subgroup Analysis: Network Meta-Analysis of Pain in Children Undergoing Day or Outpatient Surgery eFigure 11. Subgroup Analysis: Network Meta-Analysis of Pain in Children Undergoing Elective Surgery eFigure 12. Subgroup Analysis: P-Score Comparison of Digital Health Interventions for Reducing Children's Pain Across Surgery Types (Day/Outpatient vs. Elective Surgery) eFigure 13. Sensitivity analysis: Exclusion of Studies with High Risk of Bias [file jamapediatr-e253099-s001.pdf]

Supplemental Online Content

Luo Z, Zhou R, Nong K, et al. Digital health interventions in pediatric perioperative care: a network meta-analysis. *JAMA Pediatr*. Published online September 15, 2025. doi:10.1001/jamapediatrics.2025.3099

eTable 1. The search strategies used in PubMed, Embase, CENTRAL, WOS and CINAHL

eTable 2. Summary of included interventions

eTable 3. Definition of outcomes

eTable 4. References of trials included in the network meta-analysis

eTable 5. Characteristics of the included randomized controlled trials

eTable 6. Assessment of risk of bias using the Cochrane Risk of Bias Tool

eTable 7. Incoherence assessments

eTable 8. Heterogeneity (Inconsistency) assessments

eTable 9. Certainty of evidence for direct, indirect and network estimates

© 2025 Luo Z et al. *JAMA Pediatrics*.

eTable 10. Estimated effect size results for parents

eFigure 1. Assessment of the risk of bias in all included studies

eFigure 2. Comparison-adjusted funnel plot in relation to the network meta-analysis of primary outcomes

eFigure 3. Forest plots

eFigure 4. Radar Plots of Interventions Based on P-Scores for Each Outcome

eFigure 5. Network Plots of Digital Health Interventions for Secondary Outcomes

eFigure 6. League Tables of Parental Preoperative Anxiety and Postoperative Satisfaction

eFigure 7. Subgroup Analysis: Network Meta-Analysis of Anxiety in Children Undergoing Day or Outpatient Surgery

eFigure 8. Subgroup Analysis: Network Meta-Analysis of Anxiety in Children Undergoing Elective Surgery

eFigure 9. Subgroup Analysis: P-Score Comparison of Digital Health Interventions for Reducing Children's Anxiety Across Surgery Types (Day/Outpatient vs. Elective Surgery)

eFigure 10. Subgroup Analysis: Network Meta-Analysis of Pain in Children Undergoing Day or Outpatient Surgery

eFigure 11. Subgroup Analysis: Network Meta-Analysis of Pain in Children Undergoing Elective Surgery

eFigure 12. Subgroup Analysis: P-Score Comparison of Digital Health Interventions for Reducing Children's Pain Across Surgery Types (Day/Outpatient vs. Elective Surgery)

© 2025 Luo Z et al. *JAMA Pediatrics*.

eFigure 13. Sensitivity analysis: Exclusion of Studies with High Risk of Bias

This supplemental material has been provided by the authors to give readers additional information about their work.

© 2025 Luo Z et al. *JAMA Pediatrics*.

eTable 1. The search strategies used in PubMed, Embase, CENTRAL, WOS and CINAHL.

**PubMed**	
#1	General Surgery[MeSH Terms] OR Surgery, General[Title/Abstract] OR Surgery[Title/Abstract] OR Surgical Procedures, Operative[Title/Abstract] OR Operative Procedures[Title/Abstract] OR Operative Procedure[Title/Abstract] OR Procedure, Operative[Title/Abstract] OR Procedures, Operative[Title/Abstract] OR Surgical Procedure, Operative[Title/Abstract] OR Operative Surgical Procedures[Title/Abstract] OR Procedure, Operative Surgical[Title/Abstract] OR Procedures, Operative Surgical[Title/Abstract] OR Surgical Procedures[Title/Abstract] OR Procedure, Surgical[Title/Abstract] OR Procedures, Surgical[Title/Abstract] OR Surgical Procedure[Title/Abstract] OR Operative Surgical Procedure[Title/Abstract] OR Surgery, Ghost[Title/Abstract] OR Ghost Surgery[Title/Abstract] OR Perioperative Period[MeSH Terms] OR Period, Perioperative[Title/Abstract] OR Periods, Perioperative[Title/Abstract] OR Perioperative Periods[Title/Abstract]
#2	Child[MeSH Terms] OR Children[Title/Abstract] OR Boy[Title/Abstract] OR childhood[Title/Abstract] OR girl[Title/Abstract] OR infant[Title/Abstract] OR kid[Title/Abstract] OR pediatrics[Title/Abstract] OR preschool[Title/Abstract] OR school[Title/Abstract] OR toddler[Title/Abstract] OR Adolescents[Title/Abstract] OR Adolescence[Title/Abstract] OR adolescent[Title/Abstract] OR high school[Title/Abstract] OR juvenile[Title/Abstract] OR minor[Title/Abstract] OR prepubescent[Title/Abstract] OR prepuberty[Title/Abstract] OR pubescent[Title/Abstract] OR puberty[Title/Abstract] OR teen[Title/Abstract] OR teenager[Title/Abstract] OR underaged[Title/Abstract] OR youth[Title/Abstract]
#3	Cell Phone[MeSH Terms] OR Cell Phones[Title/Abstract] OR Phone, Cell[Title/Abstract] OR Phones, Cell[Title/Abstract] OR Cell Phones[Title/Abstract] OR Cellular Phone[Title/Abstract] OR Cellular Phones[Title/Abstract] OR Phone, Cellular[Title/Abstract] OR Phones, Cellular[Title/Abstract] OR Telephone, Cellular[Title/Abstract] OR Cellular Telephone[Title/Abstract] OR Cellular Telephones[Title/Abstract] OR Telephones, Cellular[Title/Abstract] OR Portable Cellular Phone[Title/Abstract] OR Cellular Phone, Portable[Title/Abstract] OR Cellular Phones, Portable[Title/Abstract] OR Portable Cellular Phones[Title/Abstract] OR Transportable Cellular Phone[Title/Abstract] OR Cellular Phone, Transportable[Title/Abstract] OR Cellular Phones, Transportable[Title/Abstract] OR Transportable Cellular Phones[Title/Abstract] OR Mobile Phone[Title/Abstract] OR Mobile Phones[Title/Abstract] OR Phone, Mobile[Title/Abstract] OR Phones, Mobile[Title/Abstract] OR Mobile Telephone[Title/Abstract] OR Mobile Telephones[Title/Abstract] OR Telephone, Mobile[Title/Abstract] OR Telephones, Mobile[Title/Abstract] OR Car Phone[Title/Abstract] OR Car Phones[Title/Abstract] OR Phone, Car[Title/Abstract] OR Phones,

© 2025 Luo Z et al. *JAMA Pediatrics*.

	Car[Title/Abstract] OR Computers[MeSH Terms] OR Computer[Title/Abstract] OR Calculators, Programmable[Title/Abstract] OR Calculator, Programmable[Title/Abstract] OR Programmable Calculator[Title/Abstract] OR Programmable Calculators[Title/Abstract] OR Hardware, Computer[Title/Abstract] OR Computer Hardware[Title/Abstract] OR Computers, Digital[Title/Abstract] OR Computer, Digital[Title/Abstract] OR Digital Computer[Title/Abstract] OR Digital Computers[Title/Abstract] OR Internet[MeSH Terms] OR World Wide Web[Title/Abstract] OR Web, World Wide[Title/Abstract] OR Wide Web, World[Title/Abstract] OR Cyberspace[Title/Abstract] OR Cyber Space[Title/Abstract] OR Microcomputers[MeSH Terms] OR Microcomputer[Title/Abstract] OR Microprocessors[Title/Abstract] OR Microprocessor[Title/Abstract] OR Computers, Personal[Title/Abstract] OR Computer, Personal[Title/Abstract] OR Personal Computer[Title/Abstract] OR Personal Computers[Title/Abstract] OR Mobile Applications[MeSH Terms] OR Application, Mobile[Title/Abstract] OR Applications, Mobile[Title/Abstract] OR Mobile Application[Title/Abstract] OR Mobile Apps[Title/Abstract] OR App, Mobile[Title/Abstract] OR Apps, Mobile[Title/Abstract] OR Mobile App[Title/Abstract] OR Portable Software Apps[Title/Abstract] OR App, Portable Software[Title/Abstract] OR Portable Software App[Title/Abstract] OR Software App, Portable[Title/Abstract] OR Portable Software Applications[Title/Abstract] OR Application, Portable Software[Title/Abstract] OR Portable Software Application[Title/Abstract] OR Software Application, Portable[Title/Abstract] OR Smartphone Apps[Title/Abstract] OR App, Smartphone[Title/Abstract] OR Apps, Smartphone[Title/Abstract] OR Smartphone App[Title/Abstract] OR Portable Electronic Apps[Title/Abstract] OR App, Portable Electronic[Title/Abstract] OR Electronic App, Portable[Title/Abstract] OR Portable Electronic App[Title/Abstract] OR Portable Electronic Applications[Title/Abstract] OR Application, Portable Electronic[Title/Abstract] OR Electronic Application, Portable[Title/Abstract] OR Portable Electronic Application[Title/Abstract] OR Motion Pictures[MeSH Terms] OR Motion Picture[Title/Abstract] OR Picture, Motion[Title/Abstract] OR Pictures, Motion[Title/Abstract] OR Films as Topic[Title/Abstract] OR Films as Topics[Title/Abstract] OR Film[Title/Abstract] OR Films[Title/Abstract] OR Cinema[Title/Abstract] OR Cinemas[Title/Abstract] OR Movies[Title/Abstract] OR Movie[Title/Abstract] OR Multimedia[Title/Abstract] OR Multimedium[Title/Abstract] OR Robotics[MeSH Terms] OR Companion Robots[Title/Abstract] OR Companion Robot[Title/Abstract] OR Robot, Companion[Title/Abstract] OR Robots, Companion[Title/Abstract] OR Telerobotics[Title/Abstract] OR Socially Assistive Robots[Title/Abstract] OR Assistive Robot, Socially[Title/Abstract] OR Assistive Robots, Socially[Title/Abstract] OR Robot, Socially Assistive[Title/Abstract] OR Robots, Socially Assistive[Title/Abstract] OR Socially Assistive Robot[Title/Abstract] OR Social Robots[Title/Abstract] OR Robot, Social[Title/Abstract] OR Robots, Social[Title/Abstract] OR Social Robot[Title/Abstract] OR Remote Operations (Robotics[Title/Abstract]) OR Operation, Remote (Robotics[Title/Abstract]) OR Operations, Remote (Robotics[Title/Abstract]) OR Remote Operation (Robotics[Title/Abstract]) OR Soft Robotics[Title/Abstract] OR Robotic,

© 2025 Luo Z et al. *JAMA Pediatrics*.

	Soft[Title/Abstract] OR Robotics, Soft[Title/Abstract] OR Soft Robotic[Title/Abstract] OR Television[MeSH Terms] OR Televisions[Title/Abstract] OR Therapy, Computer-Assisted[MeSH Terms] OR Therapy, Computer Assisted[Title/Abstract] OR Computer-Assisted Therapy[Title/Abstract] OR Computer Assisted Therapy[Title/Abstract] OR Computer-Assisted Therapies[Title/Abstract] OR Therapies, Computer-Assisted[Title/Abstract] OR Protocol-Directed Therapy, Computer-Assisted[Title/Abstract] OR Protocol Directed Therapy, Computer Assisted[Title/Abstract] OR Computer-Assisted Protocol-Directed Therapy[Title/Abstract] OR Computer Assisted Protocol Directed Therapy[Title/Abstract] OR Computer-Assisted Protocol-Directed Therapies[Title/Abstract] OR Protocol-Directed Therapies, Computer-Assisted[Title/Abstract] OR Therapies, Computer-Assisted Protocol-Directed[Title/Abstract] OR Therapy, Computer-Assisted Protocol-Directed[Title/Abstract] OR Therapy, Computer Assisted Protocol Directed[Title/Abstract] OR Video Games[MeSH Terms] OR Game, Video[Title/Abstract] OR Games, Video[Title/Abstract] OR Video Game[Title/Abstract] OR Computer Games[Title/Abstract] OR Computer Game[Title/Abstract] OR Game, Computer[Title/Abstract] OR Games, Computer[Title/Abstract] OR Virtual Reality Exposure Therapy[MeSH Terms] OR Virtual Reality Immersion Therapy[Title/Abstract] OR Virtual Reality Therapy[Title/Abstract] OR Reality Therapies, Virtual[Title/Abstract] OR Reality Therapy, Virtual[Title/Abstract] OR Therapies, Virtual Reality[Title/Abstract] OR Therapy, Virtual Reality[Title/Abstract] OR Virtual Reality Therapies[Title/Abstract] OR virtual[Title/Abstract] OR virtual reality[Title/Abstract] OR android[Title/Abstract] OR apps[Title/Abstract] OR app[Title/Abstract] OR iPad[Title/Abstract] OR iPhone[Title/Abstract] OR iPod[Title/Abstract] OR smart phone[Title/Abstract] OR smartphone[Title/Abstract] OR phone[Title/Abstract] OR device[Title/Abstract] OR tablet[Title/Abstract] OR digital[Title/Abstract] OR electronic[Title/Abstract] OR handheld[Title/Abstract] OR mobile[Title/Abstract] OR portable[Title/Abstract] OR web-based[Title/Abstract] OR artificial intelligence[Title/Abstract] OR DVD[Title/Abstract] OR lap top[Title/Abstract] OR laptop[Title/Abstract]
#4	Randomized Controlled Trial[Publication Type] OR randomized controlled trial[Title/Abstract] OR randomized[Title/Abstract] OR Clinical Trials, Randomized[Title/Abstract] OR Trials, Randomized Clinical[Title/Abstract] OR Controlled Clinical Trials, Randomized[Title/Abstract]
#5	#1 AND #2 AND #3 AND #4

**Embase**

© 2025 Luo Z et al. *JAMA Pediatrics*.

#1	children:ab,ti OR boy:ab,ti OR child:ab,ti OR childhood:ab,ti OR girl:ab,ti OR infant:ab,ti OR kid:ab,ti OR pediatrics:ab,ti OR preschool:ab,ti OR school:ab,ti OR toddler:ab,ti OR adolescents:ab,ti OR adolescence:ab,ti OR adolescent:ab,ti OR 'high school':ab,ti OR juvenile:ab,ti OR minor:ab,ti OR prepubescent:ab,ti OR prepuberty:ab,ti OR pubescent:ab,ti OR puberty:ab,ti OR teen:ab,ti OR teenager:ab,ti OR underaged:ab,ti OR youth:ab,ti
#2	'surgery, general':ab,ti OR surgery:ab,ti OR 'surgical procedures, operative':ab,ti OR 'operative procedures':ab,ti OR 'operative procedure':ab,ti OR 'procedure, operative':ab,ti OR 'procedures, operative':ab,ti OR 'surgical procedure, operative':ab,ti OR 'operative surgical procedures':ab,ti OR 'procedure, operative surgical':ab,ti OR 'procedures, operative surgical':ab,ti OR 'surgical procedures':ab,ti OR 'procedure, surgical':ab,ti OR 'procedures, surgical':ab,ti OR 'surgical procedure':ab,ti OR 'operative surgical procedure':ab,ti OR 'surgery, ghost':ab,ti OR 'ghost surgery':ab,ti OR 'perioperative period':ab,ti OR 'period, perioperative':ab,ti OR 'periods, perioperative':ab,ti OR 'perioperative periods':ab,ti
#3	'phone, cell':ab,ti OR 'phones, cell':ab,ti OR 'cell phones':ab,ti OR 'cellular phone':ab,ti OR 'cellular phones':ab,ti OR 'phone, cellular':ab,ti OR 'phones, cellular':ab,ti OR 'telephone, cellular':ab,ti OR 'cellular telephone':ab,ti OR 'cellular telephones':ab,ti OR 'telephones, cellular':ab,ti OR 'portable cellular phone':ab,ti OR 'cellular phone, portable':ab,ti OR 'cellular phones, portable':ab,ti OR 'portable cellular phones':ab,ti OR 'transportable cellular phone':ab,ti OR 'cellular phone, transportable':ab,ti OR 'cellular phones, transportable':ab,ti OR 'transportable cellular phones':ab,ti OR 'mobile phone':ab,ti OR 'mobile phones':ab,ti OR 'phone, mobile':ab,ti OR 'phones, mobile':ab,ti OR 'mobile telephone':ab,ti OR 'mobile telephones':ab,ti OR 'telephone, mobile':ab,ti OR 'telephones, mobile':ab,ti OR 'car phone':ab,ti OR 'car phones':ab,ti OR 'phone, car':ab,ti OR 'phones, car':ab,ti OR computers:ab,ti OR computer:ab,ti OR 'calculators, programmable':ab,ti OR 'calculator, programmable':ab,ti OR 'programmable calculator':ab,ti OR 'programmable calculators':ab,ti OR 'hardware, computer':ab,ti OR 'computer hardware':ab,ti OR 'computers, digital':ab,ti OR 'computer, digital':ab,ti OR 'digital computer':ab,ti OR 'digital computers':ab,ti OR internet:ab,ti OR 'world wide web':ab,ti OR 'web, world wide':ab,ti OR 'wide web, world':ab,ti OR cyberspace:ab,ti OR 'cyber space':ab,ti OR microcomputers:ab,ti OR microcomputer:ab,ti OR microprocessors:ab,ti OR microprocessor:ab,ti OR 'computers, personal':ab,ti OR 'computer, personal':ab,ti OR 'personal computer':ab,ti OR 'personal computers':ab,ti OR 'mobile applications':ab,ti OR 'application, mobile':ab,ti OR 'applications, mobile':ab,ti OR 'mobile application':ab,ti OR 'mobile apps':ab,ti OR 'app, mobile':ab,ti OR 'apps, mobile':ab,ti OR 'mobile app':ab,ti OR 'portable software apps':ab,ti OR 'app, portable software':ab,ti OR 'portable software app':ab,ti OR 'software app, portable':ab,ti OR 'portable software applications':ab,ti OR 'application, portable software':ab,ti OR 'portable software application':ab,ti OR 'software application, portable':ab,ti OR 'smartphone apps':ab,ti OR 'app, smartphone':ab,ti OR 'apps, smartphone':ab,ti OR 'smartphone app':ab,ti OR 'portable electronic apps':ab,ti OR 'app, portable electronic':ab,ti OR 'electronic app, portable':ab,ti OR 'portable electronic app':ab,ti OR 'portable

© 2025 Luo Z et al. *JAMA Pediatrics*.

	electronic applications':ab,ti OR 'application, portable electronic':ab,ti OR 'electronic application, portable':ab,ti OR 'portable electronic application':ab,ti OR 'motion picture':ab,ti OR 'picture, motion':ab,ti OR 'pictures, motion':ab,ti OR 'films as topic':ab,ti OR 'films as topics':ab,ti OR film:ab,ti OR films:ab,ti OR cinema:ab,ti OR cinemas:ab,ti OR movies:ab,ti OR movie:ab,ti OR multimedia:ab,ti OR multimedium:ab,ti OR robotics:ab,ti OR 'companion robots':ab,ti OR 'companion robot':ab,ti OR 'robot, companion':ab,ti OR 'robots, companion':ab,ti OR telerobotics:ab,ti OR 'socially assistive robots':ab,ti OR 'assistive robot, socially':ab,ti OR 'assistive robots, socially':ab,ti OR 'robot, socially assistive':ab,ti OR 'robots, socially assistive':ab,ti OR 'socially assistive robot':ab,ti OR 'social robots':ab,ti OR 'robot, social':ab,ti OR 'robots, social':ab,ti OR 'social robot':ab,ti OR 'remote operations':ab,ti OR 'soft robotics':ab,ti OR 'robotic, soft':ab,ti OR 'robotics, soft':ab,ti OR 'soft robotic':ab,ti OR televisions:ab,ti OR 'therapy, computer-assisted':ab,ti OR 'therapy, computer assisted':ab,ti OR 'computer-assisted therapy':ab,ti OR 'computer assisted therapy':ab,ti OR 'computer-assisted therapies':ab,ti OR 'therapies, computer-assisted':ab,ti OR 'protocol-directed therapy, computer-assisted':ab,ti OR 'protocol directed therapy, computer assisted':ab,ti OR 'computer-assisted protocol-directed therapy':ab,ti OR 'computer assisted protocol directed therapy':ab,ti OR 'computer-assisted protocol-directed therapies':ab,ti OR 'protocol-directed therapies, computer-assisted':ab,ti OR 'therapies, computer-assisted protocol-directed':ab,ti OR 'therapy, computer-assisted protocol-directed':ab,ti OR 'therapy, computer assisted protocol directed':ab,ti OR 'video games':ab,ti OR 'game, video':ab,ti OR 'games, video':ab,ti OR 'computer games':ab,ti OR 'computer game':ab,ti OR 'game, computer':ab,ti OR 'games, computer':ab,ti OR 'virtual reality exposure therapy':ab,ti OR 'virtual reality immersion therapy':ab,ti OR 'virtual reality therapy':ab,ti OR 'reality therapies, virtual':ab,ti OR 'reality therapy, virtual':ab,ti OR 'therapies, virtual reality':ab,ti OR 'therapy, virtual reality':ab,ti OR 'virtual reality therapies':ab,ti OR virtual:ab,ti OR 'virtual reality':ab,ti OR android:ab,ti OR apps:ab,ti OR ipad:ab,ti OR iphone:ab,ti OR ipod:ab,ti OR 'smart phone':ab,ti OR smartphone:ab,ti OR app:ab,ti OR device:ab,ti OR phone:ab,ti OR tablet:ab,ti OR digital:ab,ti OR electronic:ab,ti OR handheld:ab,ti OR mobile:ab,ti OR portable:ab,ti OR 'web based':ab,ti OR 'artificial intelligence':ab,ti OR dvd:ab,ti OR 'lap top':ab,ti OR laptop:ab,ti
#4	'crossover procedure':de OR 'double-blind procedure':de OR 'randomized controlled trial':de OR 'single-blind procedure':de OR random*:de,ab,ti OR factorial*:de,ab,ti OR crossover*:de,ab,ti OR ((cross NEXT/1 over*):de,ab,ti) OR placebo*:de,ab,ti OR ((doubl* NEAR/1 blind*):de,ab,ti) OR ((singl* NEAR/1 blind*):de,ab,ti) OR assign*:de,ab,ti OR allocat*:de,ab,ti OR volunteer*:de,ab,ti
#5	#1 AND #2 AND #3 AND #4

**CENTRAL**	
#1	(children or boy or child or childhood or girl or infant or kid or pediatrics or preschool or school or toddler or adolescents or adolescence or

© 2025 Luo Z et al. *JAMA Pediatrics*.

	adolescent or high school or juvenile or minor or prepubescent or prepuberty or pubescent or puberty or teen or teenager or underaged or youth):ti,ab,kw
#2	(Surgery, General or Surgery or Surgical Procedures, Operative or Operative Procedures or Operative Procedure or Procedure, Operative or Procedures, Operative or Surgical Procedure, Operative or Operative Surgical Procedures or Procedure, Operative Surgical or Procedures, Operative Surgical or Surgical Procedures or Procedure, Surgical or Procedures, Surgical or Surgical Procedure or Operative Surgical Procedure or Surgery, Ghost or Ghost Surgery or Perioperative Period or Period, Perioperative or Periods, Perioperative or Perioperative Periods):ti,ab,kw
#3	(Cell Phones or Phone, Cell or Phones, Cell or Cell Phones or Cellular Phone or Cellular Phones or Phone, Cellular or Phones, Cellular or Telephone, Cellular or Cellular Telephone or Cellular Telephones or Telephones, Cellular or Portable Cellular Phone or Cellular Phone, Portable or Cellular Phones, Portable or Portable Cellular Phones or Transportable Cellular Phone or Cellular Phone, Transportable or Cellular Phones, Transportable or Transportable Cellular Phones or Mobile Phone or Mobile Phones or Phone, Mobile or Phones, Mobile or Mobile Telephone or Mobile Telephones or Telephone, Mobile or Telephones, Mobile or Car Phone or Car Phones or Phone, Car or Phones, Car or Computers or Computer or Calculators, Programmable or Calculator, Programmable or Programmable Calculator or Programmable Calculators or Hardware, Computer or Computer Hardware or Computers, Digital or Computer, Digital or Digital Computer or Digital Computers or Internet or World Wide Web or Web, World Wide or Wide Web, World or Cyberspace or Cyber Space or Microcomputers or Microcomputer or Microprocessors or Microprocessor or Computers, Personal or Computer, Personal or Personal Computer or Personal Computers or Microprocessors or Microprocessor or Computers, Personal or Computer, Personal or Personal Computer or Personal Computers or Mobile Applications or Application, Mobile or Applications, Mobile or Mobile Application or Mobile Apps or App, Mobile or Apps, Mobile or Mobile App or Portable Software Apps or App, Portable Software or Portable Software App or Software App, Portable or Portable Software Applications or Application, Portable Software or Portable Software Application or Software Application, Portable or Smartphone Apps or App, Smartphone or Apps, Smartphone or Smartphone App or Portable Electronic Apps or App, Portable Electronic or Electronic App, Portable or Portable Electronic App or Portable Electronic Applications or Application, Portable Electronic or Electronic Application, Portable or Portable Electronic Application or Motion Picture or Picture, Motion or Pictures, Motion or Films as Topic or Films as Topics or Film or Films or Cinema or Cinemas or Movies or Movie or Multimedia or Multimedium or Robotics or Companion Robots or Companion Robot or Robot, Companion or Robots, Companion or Telerobotics or Socially Assistive Robots or Assistive Robot, Socially or Assistive Robots, Socially or Robot, Socially Assistive or Robots, Socially Assistive or Socially Assistive Robot or Social Robots or Robot, Social or Robots, Social or Social Robot or Remote Operations (Robotics) or Operation, Remote (Robotics) or Operations, Remote (Robotics) or Remote Operation (Robotics) or Soft

© 2025 Luo Z et al. *JAMA Pediatrics*.

	Robotics or Robotic, Soft or Robotics, Soft or Soft Robotic or Television or Televisions or Therapy, Computer-Assisted or Therapy, Computer Assisted or Computer-Assisted Therapy or Computer Assisted Therapy or Computer-Assisted Therapies or Therapies, Computer-Assisted or Protocol-Directed Therapy, Computer-Assisted or Protocol Directed Therapy, Computer Assisted or Computer-Assisted Protocol-Directed Therapy or Computer Assisted Protocol Directed Therapy or Computer-Assisted Protocol-Directed Therapies or Protocol-Directed Therapies, Computer-Assisted or Therapies, Computer-Assisted Protocol-Directed or Therapy, Computer-Assisted Protocol-Directed or Therapy, Computer Assisted Protocol Directed or Video Games or Game, Video or Games, Video or Video Game or Computer Games or Computer Game or Game, Computer or Games, Computer or Virtual Reality Exposure Therapy or Virtual Reality Immersion Therapy or Virtual Reality Therapy or Reality Therapies, Virtual or Reality Therapy, Virtual or Therapies, Virtual Reality or Therapy, Virtual Reality or Virtual Reality Therapies or virtual or virtual reality or android or apps or iPad or iPhone or iPod or smart phone or smartphone or app or device or phone or tablet or digital or electronic or handheld or mobile or portable or web-based or artificial intelligence or DVD or lap top or laptop):ti,ab,kw
#4	("randomized controlled trial"):pt
#5	#1 AND #2 AND #3 AND #4

**Web of Science**	
#1	TS=(Children or Boy or child or childhood or girl or infant or kid or pediatrics or preschool or school or toddler or Adolescents or Adolescence or adolescent or high school or juvenile or minor or prepubescent or prepuberty or pubescent or puberty or teen or teenager or underaged or youth)
#2	TS=(Surgery, General or Surgery or Surgical Procedures, Operative or Operative Procedures or Operative Procedure or Procedure, Operative or Procedures, Operative or Surgical Procedure, Operative or Operative Surgical Procedures or Procedure, Operative Surgical or Procedures, Operative Surgical or Surgical Procedures or Procedure, Surgical or Procedures, Surgical or Surgical Procedure or Operative Surgical Procedure or Surgery, Ghost or Ghost Surgery or Perioperative Period or Period, Perioperative or Periods, Perioperative or Perioperative Periods)
#3	TS=(Cell Phones or Phone, Cell or Phones, Cell or Cell Phones or Cellular Phone or Cellular Phones or Phone, Cellular or Phones, Cellular or Telephone, Cellular or Cellular Telephone or Cellular Telephones or Telephones, Cellular or Portable Cellular Phone or Cellular Phone, Portable or Cellular Phones, Portable or Portable Cellular Phones or Transportable Cellular Phone or Cellular Phone, Transportable or Cellular Phones, Transportable or Transportable Cellular Phones or Mobile Phone or Mobile Phones or Phone, Mobile or Phones, Mobile or Mobile Telephone

© 2025 Luo Z et al. *JAMA Pediatrics*.

	or Mobile Telephones or Telephone, Mobile or Telephones, Mobile or Car Phone or Car Phones or Phone, Car or Phones, Car or Computers or Computer or Calculators, Programmable or Calculator, Programmable or Programmable Calculator or Programmable Calculators or Hardware, Computer or Computer Hardware or Computers, Digital or Computer, Digital or Digital Computer or Digital Computers or Internet or World Wide Web or Web, World Wide or Wide Web, World or Cyberspace or Cyber Space or Microcomputers or Microcomputer or Microprocessors or Microprocessor or Computers, Personal or Computer, Personal or Personal Computer or Personal Computers or Microprocessors or Microprocessor or Computers, Personal or Computer, Personal or Personal Computer or Personal Computers or Mobile Applications or Application, Mobile or Applications, Mobile or Mobile Application or Mobile Apps or App, Mobile or Apps, Mobile or Mobile App or Portable Software Apps or App, Portable Software or Portable Software App or Software App, Portable or Portable Software Applications or Application, Portable Software or Portable Software Application or Software Application, Portable or Smartphone Apps or App, Smartphone or Apps, Smartphone or Smartphone App or Portable Electronic Apps or App, Portable Electronic or Electronic App, Portable or Portable Electronic App or Portable Electronic Applications or Application, Portable Electronic or Electronic Application, Portable or Portable Electronic Application or Motion Picture or Picture, Motion or Pictures, Motion or Films as Topic or Films as Topics or Film or Films or Cinema or Cinemas or Movies or Movie or Multimedia or Multimedium or Robotics or Companion Robots or Companion Robot or Robot, Companion or Robots, Companion or Telerobotics or Socially Assistive Robots or Assistive Robot, Socially or Assistive Robots, Socially or Robot, Socially Assistive or Robots, Socially Assistive or Socially Assistive Robot or Social Robots or Robot, Social or Robots, Social or Social Robot or Remote Operations (Robotics) or Operation, Remote (Robotics) or Operations, Remote (Robotics) or Remote Operation (Robotics) or Soft Robotics or Robotic, Soft or Robotics, Soft or Soft Robotic or Television or Televisions or Therapy, Computer-Assisted or Therapy, Computer Assisted or Computer-Assisted Therapy or Computer Assisted Therapy or Computer-Assisted Therapies or Therapies, Computer-Assisted or Protocol-Directed Therapy, Computer-Assisted or Protocol Directed Therapy, Computer Assisted or Computer-Assisted Protocol-Directed Therapy or Computer Assisted Protocol Directed Therapy or Computer-Assisted Protocol-Directed Therapies or Protocol-Directed Therapies, Computer-Assisted or Therapies, Computer-Assisted Protocol-Directed or Therapy, Computer-Assisted Protocol-Directed or Therapy, Computer Assisted Protocol Directed or Video Games or Game, Video or Games, Video or Video Game or Computer Games or Computer Game or Game, Computer or Games, Computer or Virtual Reality Exposure Therapy or Virtual Reality Immersion Therapy or Virtual Reality Therapy or Reality Therapies, Virtual or Reality Therapy, Virtual or Therapies, Virtual Reality or Therapy, Virtual Reality or Virtual Reality Therapies or virtual or virtual reality or android or apps or iPad or iPhone or iPod or smart phone or smartphone or app or device or phone or tablet or digital or electronic or handheld or mobile or portable or web-based or artificial intelligence or DVD or lap top or laptop)

© 2025 Luo Z et al. *JAMA Pediatrics*.

#4	((TS=(randomized controlled trial)) OR TS=(randomized controlled)) OR TS=(randomized control)
#5	#1 AND #2 AND #3 AND #4

**CINAHL**	
S1	AB (Children or Boy or child or childhood or girl or infant or kid or pediatrics or preschool or school or toddler or Adolescents or Adolescence or adolescent or high school or juvenile or minor or prepubescent or prepuberty or pubescent or puberty or teen or teenager or underaged or youth)
S2	AB (Surgery, General or Surgery or Surgical Procedures, Operative or Operative Procedures or Operative Procedure or Procedure, Operative or Procedures, Operative or Surgical Procedure, Operative or Operative Surgical Procedures or Procedure, Operative Surgical or Procedures, Operative Surgical or Surgical Procedures or Procedure, Surgical or Procedures, Surgical or Surgical Procedure or Operative Surgical Procedure or Surgery, Ghost or Ghost Surgery or Perioperative Period or Period, Perioperative or Periods, Perioperative or Perioperative Periods)
S3	AB (Cell Phones or Phone, Cell or Phones, Cell or Cell Phones or Cellular Phone or Cellular Phones or Phone, Cellular or Phones, Cellular or Telephone, Cellular or Cellular Telephone or Cellular Telephones or Telephones, Cellular or Portable Cellular Phone or Cellular Phone, Portable or Cellular Phones, Portable or Portable Cellular Phones or Transportable Cellular Phone or Cellular Phone, Transportable or Cellular Phones, Transportable or Transportable Cellular Phones or Mobile Phone or Mobile Phones or Phone, Mobile or Phones, Mobile or Mobile Telephone or Mobile Telephones or Telephone, Mobile or Telephones, Mobile or Car Phone or Car Phones or Phone, Car or Phones, Car or Computers or Computer or Calculators, Programmable or Calculator, Programmable or Programmable Calculator or Programmable Calculators or Hardware, Computer or Computer Hardware or Computers, Digital or Computer, Digital or Digital Computer or Digital Computers or Internet or World Wide Web or Web, World Wide or Wide Web, World or Cyberspace or Cyber Space or Microcomputers or Microcomputer or Microprocessors or Microprocessor or Computers, Personal or Computer, Personal or Personal Computer or Personal Computers or Microprocessors or Microprocessor or Computers, Personal or Computer, Personal or Personal Computer or Personal Computers or Mobile Applications or Application, Mobile or Applications, Mobile or Mobile Application or Mobile Apps or App, Mobile or Apps, Mobile or Mobile App or Portable Software Apps or App, Portable Software or Portable Software App or Software App, Portable or Portable Software Applications or Application, Portable Software or Portable Software Application or Software Application, Portable or Smartphone Apps or App, Smartphone or Apps, Smartphone or Smartphone App or Portable Electronic Apps or App, Portable Electronic or Electronic App, Portable or Portable Electronic App

© 2025 Luo Z et al. *JAMA Pediatrics*.

	or Portable Electronic Applications or Application, Portable Electronic or Electronic Application, Portable or Portable Electronic Application or Motion Picture or Picture, Motion or Pictures, Motion or Films as Topic or Films as Topics or Film or Films or Cinema or Cinemas or Movies or Movie or Multimedia or Multimedium or Robotics or Companion Robots or Companion Robot or Robot, Companion or Robots, Companion or Telerobotics or Socially Assistive Robots or Assistive Robot, Socially or Assistive Robots, Socially or Robot, Socially Assistive or Robots, Socially Assistive or Socially Assistive Robot or Social Robots or Robot, Social or Robots, Social or Social Robot or Remote Operations (Robotics) or Operation, Remote (Robotics) or Operations, Remote (Robotics) or Remote Operation (Robotics) or Soft Robotics or Robotic, Soft or Robotics, Soft or Soft Robotic or Television or Televisions or Therapy, Computer-Assisted or Therapy, Computer Assisted or Computer-Assisted Therapy or Computer Assisted Therapy or Computer-Assisted Therapies or Therapies, Computer-Assisted or Protocol-Directed Therapy, Computer-Assisted or Protocol Directed Therapy, Computer Assisted or Computer-Assisted Protocol-Directed Therapy or Computer Assisted Protocol Directed Therapy or Computer-Assisted Protocol-Directed Therapies or Protocol-Directed Therapies, Computer-Assisted or Therapies, Computer-Assisted Protocol-Directed or Therapy, Computer-Assisted Protocol-Directed or Therapy, Computer Assisted Protocol Directed or Video Games or Game, Video or Games, Video or Video Game or Computer Games or Computer Game or Game, Computer or Games, Computer or Virtual Reality Exposure Therapy or Virtual Reality Immersion Therapy or Virtual Reality Therapy or Reality Therapies, Virtual or Reality Therapy, Virtual or Therapies, Virtual Reality or Therapy, Virtual Reality or Virtual Reality Therapies or virtual or virtual reality or android or apps or iPad or iPhone or iPod or smart phone or smartphone or app or device or phone or tablet or digital or electronic or handheld or mobile or portable or web-based or artificial intelligence or DVD or lap top or laptop)
S4	TX allocat* random* OR (MH "Quantitative Studies") OR (MH "Placebos") OR TX placebo* OR TX random* allocat* OR (MH "Random Assignment") OR TX randomi* control* trial* OR TX ((singl* n1 blind*) OR (singl* n1 mask*)) OR TX ((doubl* n1 blind*) OR (doubl* n1 mask*)) OR TX ((tripl* n1 blind*) OR (tripl* n1 mask*)) OR TX ((trebl* n1 blind*) OR (trebl* n1 mask*)) OR TX clinic* n1 trial* OR PT Clinical trial OR (MH "Clinical Trials+")
S5	S1 AND S2 AND S3 AND S4

© 2025 Luo Z et al. *JAMA Pediatrics*.

eTable 2. Summary of included interventions.

Interventions of interest	Definition
Virtual reality	A computer-generated simulation that allows users to interact with a 3D environment through specialized headsets, providing immersive distraction.
Video(2D)	A non-interactive audiovisual intervention that involves watching content on a screen for entertainment or distraction. This may include watching a favorite cartoon video, educational content, or other engaging videos.
Game(2D)	A digital, interactive intervention using age-appropriate tablet games designed to engage children through entertainment and cognitive distraction. This includes educational games, video games, serious games, and other interactive digital games.
An interactive robot	A robotic system programmed to interact with patients, often using speech, gestures, and facial expressions to provide distraction, comfort, and engagement.

Comparison interventions	Definition
Midazolam	A short-acting benzodiazepine commonly used for preoperative sedation and anxiolysis, acting on GABA receptors to induce relaxation and reduce anxiety.
Enhanced control	A control intervention that includes structured text messages providing information on pre- and post-operative care, puppet play scenarios to help children understand medical procedures in an interactive way, and educational booklets or pamphlets that provide additional information to parents and children about the surgery and recovery process.
Control	Standard of care, including verbal information, routine preparations, and explanations of the planned surgical procedure. Additional methods may include storytelling, toy-playing, nonprocedural talk, humor, or quiet rest. No additional structured interventions are provided.

© 2025 Luo Z et al. *JAMA Pediatrics*.

eTable 3. Definition of outcomes.

Outcome	Definition	Effect measure
Children - preoperative anxiety	Change of anxiety score from baseline to follow-up. Anxiety data recorded closest to the time of anesthesia induction preparation were prioritized, with the measurement that best reflected the patient's condition immediately prior to induction selected if multiple time points were available. Different studies measured anxiety using different questionnaires, including mYPAS (Modified Yale Preoperative Anxiety Scale), CmYPAS (Chinese version of the Modified Yale Preoperative Anxiety Scale), STAI-C (State-Trait Anxiety Inventory - Child version), CSA (Children's State Anxiety), CAS-S (Child Anxiety Scale-State), CAM-S (Children's Anxiety Meter Scale), and VAS (Visual Analog Scale).	Standardized mean difference
Children - postoperative pain	Change of pain score from baseline to follow-up. For painful procedures, data from the first procedure or the closest time point thereafter were used. In crossover trials, data from the first period were preferentially included. When multiple time points or observers were available, a composite score was generated. The sensory component of pain was prioritized for inclusion in the network meta-analysis over cognitive and affective components. Different studies measured pain using different questionnaires, including WBFPRS (Wong-Baker Faces Pain Rating Scale), FPS-r (Faces Pain Scale Revised), mCHEOPS (Modified Children's Hospital of Eastern Ontario Pain Score), VAS (Visual Analog Scale), FLACC (Face, Legs, Activity, Cry, and Consolability), and NRS (Numeric Rating Scale).	Standardized mean difference
Children - emergence delirium	Emergence delirium refers to the period of confusion or agitation that may occur after anesthesia in children. The PAED (Pediatric Anesthesia Emergence Delirium Scale) is used to measure the severity of delirium.	Mean difference
Children - induction compliance	Induction compliance refers to the child's cooperation during anesthesia induction. Measured by the ICC (Induction Compliance Checklist), this scale assesses the child's	Mean difference

© 2025 Luo Z et al. *JAMA Pediatrics*.

	response to the anesthesia induction procedure.	
Parents - preoperative anxiety	Anxiety data recorded closest to the time of anesthesia induction preparation were prioritized, with the measurement that best reflected the parent's condition immediately prior to induction selected if multiple time points were available. Different studies measured anxiety using different questionnaires, including STAI (State-Trait Anxiety Inventory), STOA (State-Trait Operation Anxiety Inventory), SAS (Self-rating Anxiety Scale), and BAI (Beck Anxiety Inventory).	Standardized mean difference
Parents - postoperative satisfaction	Postoperative satisfaction is measured by assessing how satisfied parents are with the care their child received post-surgery. Different studies measured satisfaction using different methods, including verbal score from 0 (not satisfied) to 10 (highly satisfied), VAS (Visual Analog Scale), and NRS (Numeric Rating Scale).	Standardized mean difference

© 2025 Luo Z et al. *JAMA Pediatrics*.

eTable 4. References of trials included in the network meta-analysis.

1. Jung MJ, Libaw JS, Ma K, Whitlock EL, Feiner JR, Sinskey JL. Pediatric Distraction on Induction of Anesthesia With Virtual Reality and Perioperative Anxiolysis: A Randomized Controlled Trial. *Anesth Analg*. 2021;132(3):798-806. doi:10.1213/ANE.0000000000005004

2. Bozkul G, Karakul A, Duzkaya DS, Dilsen S. Effect of short film video and video-based education on fear, pain, and satisfaction of children undergoing day surgery. *J Pediatr Nurs*. 2024;75((Bozkul G.; Karakul A.; Düzkaya D.S.) Tarsus University, Faculty of Health Sciences, Department of Nursing,):49-56. doi:10.1016/j.pedn.2023.11.029

3. Baghele A, Dave N, DIas R, Shah H. Effect of preoperative education on anxiety in children undergoing day-care surgery. *Indian journal of anaesthesia*. 2019;63(7):565-570. doi:10.4103/ija.IJA_37_19

4. Buffel C, van Aalst J, Bangels A, et al. A Web-Based Serious Game for Health to Reduce Perioperative Anxiety and Pain in Children (CliniPup): Pilot Randomized Controlled Trial. *JMIR serious games*. 2019;7(2):e12431. doi:10.2196/12431

5. Buyuk ET, Odabasoglu E, Uzsen H, Koyun M. The effect of virtual reality on Children's anxiety, fear, and pain levels before circumcision. *J Pediatr Urol*. 2021;17(4):567.e1-567.e8. doi:10.1016/j.jpurol.2021.04.008

6. Chartrand J, Tourigny J, MacCormick J. The effect of an educational pre-operative DVD on parents' and children's outcomes after a same-day surgery: a randomized controlled trial. *Journal of Advanced Nursing (John Wiley & Sons, Inc)*. 2017;73(3):599-611. doi:10.1111/jan.13161

7. Clausen NG, Madsen D, Rosenkilde C, Hasfeldt-Hansen D, Larsen LG, Hansen TG. The Use of Tablet Computers to Reduce Preoperative Anxiety in Children Before Anesthesia: A Randomized Controlled Study. *Journal of perianesthesia nursing : official journal of the american society of perianesthesia nurses*. 2021;36(3):275-278. doi:10.1016/j.jopan.2020.09.012

8. Cumino DO, Vieira JE, Lima LC, Stievano LP, Silva RAP, Mathias LAST. Smartphone-based behavioural intervention alleviates children's anxiety during anaesthesia induction: A randomised controlled trial. *European journal of anaesthesiology*. 2017;34(3):169-175. doi:10.1097/EJA.0000000000000589

9. Dost B, Komurcu O, Bilgin S, et al. Is Preoperative Anxiety Affected by Watching Short Videos on Social Media? A Prospective Randomized Study.

© 2025 Luo Z et al. *JAMA Pediatrics*.

Journal of perianesthesia nursing : official journal of the american society of perianesthesia nurses. 2023;((Dost B., burhandost@hotmail.com; Komurcu O.; Bilgin S.; Turunc E.; Ozden G.G.; Baris S.) Department of Anesthesiology and Reanimation, Faculty of Medicine, Ondokuz Mayis University, Samsun, Turkey). doi:10.1016/j.jopan.2023.01.006

10. Eijlers R, Dierckx B, Staals LM, et al. Virtual reality exposure before elective day care surgery to reduce anxiety and pain in children: A randomised controlled trial. *Eur J Anaesthesiol*. 2019;36(10):728-737. doi:10.1097/EJA.0000000000001059

11. Evren Sahin K, Karkiner A. The effect of using tablet computer on surgical stress: A single-blinded randomized controlled trial. *Journal of pediatric urology*. 2022;18(3):340.e1-340.e9. doi:10.1016/j.jpurol.2022.03.008

12. Franco Castanys T, Jiménez Carrión A, Ródenas Gómez F, et al. Effects of virtual tour on perioperative pediatric anxiety. *Paediatric anaesthesia*. 2023;33(5):377-386. doi:10.1111/pan.14639

13. Härter V, Barkmann C, Wiessner C, Rupprecht M, Reinshagen K, Trah J. Effects of Educational Video on Pre-operative Anxiety in Children - A Randomized Controlled Trial. *Frontiers in pediatrics*. 2021;9((Härter V.; Reinshagen K.; Trah J., jtrah@uke.de) Department of Pediatric Surgery, University Medical Center Hamburg-Eppendorf, Hamburg, Germany). doi:10.3389/fped.2021.640236

14. Hou H, Li X, Song Y, et al. Effect of interactive, multimedia-based home-initiated education on preoperative anxiety inchildren and their parents: a single-center randomized controlled trial. *BMC anesthesiology*. 2023;23(1):95. doi:10.1186/s12871-023-02055-7

15. Huang YL, Lei YQ, Liu JF, Cao H, Yu XR, Chen Q. The music video therapy in postoperative analgesia in preschool children after cardiothoracic surgery. *Journal of Cardiac Surgery*. 2021;36(7):2308-2313. doi:10.1111/jocs.15551

16. Huntington C, Liossi C, Donaldson AN, et al. On-line preparatory information for children and their families undergoing dental extractions under general anesthesia: A phase III randomized controlled trial. *Paediatric anaesthesia*. 2018;28(2):157-166. doi:10.1111/pan.13307

17. Kerimaa H, Hakala M, Haapea M, et al. Effectiveness of a Mobile App Intervention for Preparing Preschool Children and Parents for Day Surgery: Randomized Controlled Trial. *J Med Internet Res*. 2023;25(1). doi:10.2196/46989

© 2025 Luo Z et al. *JAMA Pediatrics*.

18. Kerimoglu B, Neuman A, Paul J, Stefanov DG, Twersky R. Anesthesia induction using video glasses as a distraction tool for the management of preoperative anxiety in children. *Anesthesia and analgesia*. 2013;117(6):1373-1379. doi:10.1213/ANE.0b013e3182a8c18f

19. Kumar A, Das S, Chauhan S, Kiran U, Satapathy S. Perioperative Anxiety and Stress in Children Undergoing Congenital Cardiac Surgery and Their Parents: Effect of Brief Intervention—A Randomized Control Trial. *Journal of cardiothoracic and vascular anesthesia*. 2019;33(5):1244-1250. doi:10.1053/j.jvca.2018.08.187

20. Lee JH, Jung HK, Lee GG, Kim HY, Park SG, Woo SC. Effect of behavioral intervention using smartphone application for preoperative anxiety in pediatric patients. *Korean journal of anesthesiology*. 2014;65(6):508-518. doi:10.4097/kjae.2013.65.6.508

21. Levay MM, Sumser MK, Vargo KM, et al. The effect of active distraction compared to midazolam in preschool children in the perioperative setting: A randomized controlled trial. *Journal of pediatric nursing*. 2023;68((Levay M.M., levaym@ccf.org; Sumser M.K., sumserm@ccf.org; Danford C.A., danfordc@ccf.org) Office of Nursing Research&Innovation, USA; Cleveland Clinic, 9500 Euclid Avenue, Cleveland, OH 44195, USA):35-43. doi:10.1016/j.pedn.2022.09.021

22. Liguori S, Stacchini M, Ciofi D, Olivini N, Bisogni S, Festini F. Effectiveness of an App for Reducing Preoperative Anxiety in Children: A Randomized Clinical Trial. *JAMA pediatrics 170 (8) (no pagination), 2016 Date of publication: august 2016*. 2016;170(8):e160533. doi:10.1001/jamapediatrics.2016.0533

23. Luo W, Chen C, Zhou W, et al. Biophilic virtual reality on children's anxiety and pain during circumcision: A randomized controlled study. *J Pediatr Urol*. 2023;19(2):201-210. doi:10.1016/j.jpurol.2022.10.023

24. Marechal C, Berthiller J, Tosetti S, et al. Children and parental anxiolysis in paediatric ambulatory surgery: a randomized controlled study comparing 0.3 mg kg-1 midazolam to tablet computer based interactive distraction. *BJA: The British Journal of Anaesthesia*. 2017;118(2):247-253. doi:10.1093/bja/aew436

25. Matthyssens L, Vanhulle A, Seldenslach L, Vander Stichele G, Coppens M, Van Hoecke E. A pilot study of the effectiveness of a serious game CliniPup® on perioperative anxiety and pain in children. *Journal of pediatric surgery*. 2020;55(2):304-311. doi:10.1016/j.jpedsurg.2019.10.031

© 2025 Luo Z et al. *JAMA Pediatrics*.

26. Mete İzci S, Çetinkaya B. The impact of digital storytelling for children during paediatric day surgery on anxiety and negative emotional behaviors: Randomized controlled trial. *J Pediatr Nurs*. 2024;((Mete İzci S., selvermete50@gmail.com) Pediatric Nursing, Institute for Health Sciences, Pamukkale University, Denizli, Turkey). doi:10.1016/j.pedn.2024.04.034

27. Mifflin KA, Hackmann T, Chorney JM. Streamed video clips to reduce anxiety in children during inhaled induction of anesthesia. *Anesthesia and analgesia*. 2012;115(5):1162-1167. doi:10.1213/ANE.0b013e31824d5224

28. Mihandoust S, Joseph A, Browning MHEM, Cha JS, Gonzales A, Markowitz J. Can pre-visit exposure to virtual tours of healthcare facilities help reduce child and parent anxiety during outpatient surgical procedures? *Appl Ergon*. 2024;119((Mihandoust S.; Joseph A., Anjalij@clemson.edu) Center for Health Facilities Design and Testing, School of Architecture, Clemson University, Clemson, SC, United States). doi:10.1016/j.apergo.2024.104308

29. Nair T, Choo CSC, Abdullah NS, et al. Home-Initiated-Programme-to-Prepare-for-Operation: evaluating the effect of an animation video on peri-operative anxiety in children: A randomised controlled trial. *European journal of anaesthesiology*. 2021;38(8):880-887. doi:10.1097/EJA.0000000000001385

30. Patel A, Schieble T, Davidson M, et al. Distraction with a hand-held video game reduces pediatric preoperative anxiety. *Paediatric anaesthesia*. 2006;16(10):1019-1027. doi:10.1111/j.1460-9592.2006.01914.x

31. Ryu JH, Ko D, Han JW, et al. The proper timing of virtual reality experience for reducing preoperative anxiety of pediatric patients: A randomized clinical trial. *Frontiers in pediatrics*. 2022;10((Ryu J.-H.; Han S.-H., noninvasive@hanmail.net) Medical Virtual Reality Research Group, Department of Anesthesiology and Pain Medicine, Seoul National University College of Medicine, Seoul, South Korea). doi:10.3389/fped.2022.899152

32. Ryu JH, Park JW, Nahm FS, et al. The effect of gamification through a virtual reality on preoperative anxiety in pediatric patients undergoing general anesthesia: A prospective, randomized, and controlled trial. *J Clin Med*. 2018;7(9). doi:10.3390/jcm7090284

33. Ryu JH, Park SJ, Park JW, et al. Randomized clinical trial of immersive virtual reality tour of the operating theatre in children before anaesthesia. *British Journal of Surgery*. 2017;104(12):1628-1633. doi:10.1002/bjs.10684

© 2025 Luo Z et al. *JAMA Pediatrics*.

34. Ryu J, Oh A, Yoo H, Kim J, Park J, Han S. The effect of an immersive virtual reality tour of the operating theater on emergence delirium in children undergoing general anesthesia: A randomized controlled trial. *Pediatric Anesthesia*. 2019;29(1):98-105. doi:10.1111/pan.13535

35. Seiden SC, McMullan S, Sequera-Ramos L, et al. Tablet-based Interactive Distraction (TBID) vs oral midazolam to minimize perioperative anxiety in pediatric patients: a noninferiority randomized trial. *Paediatric anaesthesia*. 2014;24(12):1217-1223. doi:10.1111/pan.12475

36. Specht B, Buse C, Phelps J, et al. Virtual Reality after Surgery—A Method to Decrease Pain After Surgery in Pediatric Patients. *American surgeon*. 2023;89(4):31348211032204. doi:10.1177/00031348211032204

37. Stewart B, Cazzell MA, Pearcy T. Single-Blinded Randomized Controlled Study on Use of Interactive Distraction Versus Oral Midazolam to Reduce Pediatric Preoperative Anxiety, Emergence Delirium, and Postanesthesia Length of Stay. *Journal of perianesthesia nursing : official journal of the american society of perianesthesia nurses*. 2019;34(3):567-575. doi:10.1016/j.jopan.2018.08.004

38. Tang X, Zhang M, Yang L, et al. Individual cartoon video for alleviating perioperative anxiety and reducing emergence delirium in children: a prospective randomised trial. *BMJ paediatrics open*. 2023;7(1). doi:10.1136/bmjpo-2023-001854

39. Topçu SY, Semerci R, Kostak MA, Güray Ö, Sert S, Yavuz G. The effect of an immersive virtual reality tour of the operating theater on emergence delirium in children undergoing general anesthesia: A randomized controlled trial. *Journal of pediatric nursing*. 2023;68((Topçu S.Y.) Trakya University, Faculty of Health Science, Department of Surgical Nursing, Edirne, Turkey):e50-e57. doi:10.1016/j.pedn.2022.11.009

40. Tuncay S, Tüfekci FG. The effect of nursing interventions with therapeutic play and video animations prepared with psychodrama technique in reducing fear, anxiety, and pain of children at male circumcision: A randomized controlled study. *International journal of urology*. 2023;30(7):592-599. doi:10.1111/iju.15184

41. Uysal G, Düzkaya DS, Bozkurt G, Akdağ MY, Akça SÖ. The effect of watching videos using virtual reality during operating room transfer on the fear andanxiety of children aged 6-12 undergoing inguinal hernia surgery: A randomized controlled trial. *Journal of pediatric nursing*. 2023;105((Uysal G., gulzadeuysal@subu.edu.tr) Sakarya University of Applied Sciences, Faculty of Health Sciences, Sakarya, Turkey):N.PAG-N.PAG. doi:10.1016/j.pedn.2023.06.022

© 2025 Luo Z et al. *JAMA Pediatrics*.

42. Wang Y, Chu LY, Wang SS, et al. [Preoperative video distraction alleviates separation anxiety and improves induction compliance of preschool children: a randomized controlled clinical trial]. *Zhonghua Yi Xue Za Zhi*. 2021;101(26):2066-2070. doi:10.3760/cma.j.cn112137-20201224-03458

43. Wu Y, Chen J, Ma W, Guo L, Feng H. Virtual reality in preoperative preparation of children undergoing general anesthesia: a randomized controlled study. *Anaesthesiologie*. 2022;71((Wu Y.) Department of Anaesthesiology, First Affiliated Hospital, School of Medicine, Zhejiang University, Hangzhou, China):S204-S211. doi:10.1007/s00101-022-01177-w

44. Yang JY, Lee H, Zhang Y, Lee JU, Park JH, Yun EK. The Effects of Tonsillectomy Education Using Smartphone Text Message for Mothers and Children Undergoing Tonsillectomy: A Randomized Controlled Trial. *Telemedicine journal and e-health*. 2016;22(11):921-928. doi:10.1089/tmj.2016.0019

45. Yaz S, Yilmaz H. The effect of watching videos using virtual reality during operating room transfer on the fear andanxiety of children aged 6-12 undergoing inguinal hernia surgery: A randomized controlled trial. *J Pediatr Nurs*. 2022;37((Uysal G., gulzadeuysal@subu.edu.tr) Sakarya University of Applied Sciences, Faculty of Health Sciences, Sakarya, Turkey):E152-E157. doi:10.1016/j.jopan.2021.04.015

46. Carbó A, Tresandí D, Tril C, Fernández-Rodríguez D, Carrero E. Usefulness of a virtual reality educational program for reducing preoperative anxiety in children: A randomised, single-centre clinical trial. *Eur J Anaesthesiol*. 2024;41(9):657-667. doi:10.1097/EJA.0000000000002032

47. Chen H, Chen L, Zhu C, Li S, Zhou J, Liu C. Immersive Virtual Reality Versus Video Distraction for the Management of Emergence Delirium in Children: A Randomized Controlled Study. *J Perianesth Nurs*. 2025;40(2):318-325. doi:10.1016/j.jopan.2024.05.006

48. Turgut A, Özcan İlçe A, Öztürk H. The Effect of Immersive Virtual Reality Application on Anxiety, Pain, and Parental Satisfaction in the Perioperative Process of Children: A Randomized Controlled Trial. *Pain Manag Nurs*. 2024;25(6):584-590. doi:10.1016/j.pmn.2024.06.002

49. Vinay AP, Karna ST, Ahmad Z, Waindeskar V, Ahmed R, Kuttan KA. Utility of interactive videogame in allaying preoperative anxiety in pediatric surgical patients - A randomized controlled study. *J Postgrad Med*. 2024;70(4):198-203. doi:10.4103/jpgm.jpgm_465_24

© 2025 Luo Z et al. *JAMA Pediatrics*.

eTable 5. Characteristics of the included randomized controlled trials.

Author (Year)	Participants		Country	Procedures	Intervention and Control Group (Number of Patients)	Moment of digital technology	Instruments and time points of outcome assessment
	N	Age					
Buffel et al(2019)	20	6-10 years	Belgium	Ambulatory surgery	Game: a web-based serious game (12)<br>Standard of care (8)	Preoperative	**Anxiety:**<br>Child: mYPAS (preoperative)<br>Parent: STAI (preoperative)<br>**Pain:** WBFPRS (postoperative)
Kerimoglu et al(2013)	64	4-9 years	America	Ambulatory surgery	Virtual reality: video glasses (Vuzix®, Vuzix Corporation, Rochester, NY) (32)<br>Midazolam, 0.3 mg/kg, PO (32)	Preoperative	**Anxiety:** mYPAS at baseline (T1), at time of transport to the operating room (OR) 20 minutes later (T2), and during mask induction in the OR (T3).
Luo et al(2023)	106	7-18 years	China	Circumcision surgery	Virtual reality：VR glasses (weigh 350 g, Unity3D 2018.3.10f1, Shu Rui Medical) (70)<br>Standard medical care without wearing VR glasses (36)	Throughout the procedure	**Anxiety**：CmYPAS and VAS at the end of the surgery<br>**Pain**：FPS-r at the end of the surgery
Marechal et al(2017)	118	4-10 years	France	Ambulatory surgery	Game: an age-appropriate tablet game (60)<br>Midazolam,0.3 mg/kg, PO or PR (58)	Preoperative and Postoperation	**Anxiety**：<br>Child: mYPAS at admission surgical ward (T1), parent separation (T2), anaesthesia induction (T3) and when back to surgical ward (T4)<br>Parent: STAI at T1, T2 and T4<br>**Parental satisfaction:** verbal score

© 2025 Luo Z et al. *JAMA Pediatrics*.

							from 0 (not satisfied) to 10 (highly satisfied) at T4
Patel et al(2006)	76	4-12 years	America	Ambulatory surgery	Game: a hand-held video game (38)<br>Midazolam, 0.5 mg/kg, PO (38)	Preoperative	**Anxiety：** mYPAS at baseline (T1) and induction of anaesthesia (T2)
Hou et al(2023)	78	4-9 years	China	Strabismus surgery	Video: preoperative educational information (40)<br>Enhanced control: Question-and-answer introduction (38)	Preoperative	**Anxiety:**<br>Child: mYPAS-SF at baseline (T0), in the preoperative waiting area (T1), during separation from parents and moving to the operating room (T2), and at anaesthesia induction (T3)<br>Parent: SAS and VAS at T0 and T2<br>**Compliance:** ICC at T3
Baghele et al(2019)	94	7-12 years	Indian	Day-care procedures	Video: an informative video about the anaesthetic technique (47)<br>Standard of care (47)	Preoperative	**Anxiety:** VAS at the preanaesthetic check
Kerimaa et al(2023)	70	2-6 years	Finland	Elective day surgery	Video: informing users about the phases of the surgery (36)<br>Standard of care(34)	Preoperative	**Pain:** VAS and WBS at home (T1), before the procedure (T2), postsurgery (T3), and during the three days at home after discharge (T4)<br>**Anxiety (Parent):** STAI at T1, T2, T3, T4
Liguori et al(2016)	40	6-11 years	Italian	A surgical intervention (eg, phimosis,	Video: explaining to each other what is in the OR (20)<br>Standard of care (20)	Preoperative	**Anxiety:** mYPAS at baseline and during transfer to the OR

© 2025 Luo Z et al. *JAMA Pediatrics*.

				abdominal hernia, or orthopedic corrections)			
Härter et al(2021)	90	6-17 years	Germany	Elective surgery	Video: explaining the perioperative procedures (45)<br>Standard of care (45)	Preoperative	**Anxiety:** STOA at before administering the intervention (T1) and directly after the intervention but before surgery (T2), and 1 day after surgery (T3)
Nair et al(2021)	113	6-10 years	Singapore	Elective surgery	Video: illustrating the events leading up to surgery (59)<br>Standard of care (54)	Preoperative	**Anxiety:** VAS at induction of anaesthesia<br>**Compliance:** ICC at induction of anaesthesia
Tang et al(2023)	80	3-7 years	China	Adenoidectomy and tonsillectomy	Video: watching a favourite cartoon video (40)<br>Standard of care (40)	Preoperative	**Anxiety:** mYPAS at baseline (T0), on entering the operating room (T1), during the induction of anaesthesia (T2) and on leaving the PACU (T6)<br>**Pain:** FLACC at immediately after extubation (T3), on arriving in the PACU (T4), 30 min after arriving in the PACU (T5) and on leaving the PACU (T6)<br>**Compliance:** ICC at T2<br>**Emergence delirium:** PAED at T3, T4, T5 and T6

© 2025 Luo Z et al. *JAMA Pediatrics*.

Dost et al(2023)	69	5-12 years	Turkey	Elective surgery	Video: view short videos on a social media platform of their choice (35)<br>Standard of care (34)	Preoperative	**Anxiety:** mYPAS at arrival in the waiting room (T1), right before being taken to the operating room (OR) (T2), on entering the OR (T3), and during anaesthesia induction (T4)
Huntington et al(2017)	111	5-7 years	America	Tooth extraction (day surgery)	Game: a hand-washing game (55)<br>Standard of care (56)	Preoperative	**Anxiety:** mYPAS at the anaesthetic induction<br>**Parental satisfaction：** VAS immediately prior to discharge and TEI 48 hours later
Jung et al(2021)	70	5-12 years	America	Elective surgery	Virtual reality: Samsung Gear VR headset (Samsung Electronics, Suwon, South Korea) (33)<br>Standard of care (37)	Preoperative	**Anxiety:**<br>Child: mYPAS at baseline (T0), on entering the operating room (T1), and during induction of general anaesthesia (T2)<br>Parent: STAI at T0 and after induction of general anesthesia (T3)<br>**Compliance:** ICC at T2<br>**Parental satisfaction:** 21-item parental and patient Parental satisfaction questionnaire at T3
Kumar et al(2019)	60	5-15 years	Indian	Cardiac surgery	Game: video games (30)<br>Standard of care (30)	Preoperative	**Anxiety:**<br>Child: STAI-C at one day before surgery and on the third postoperative day<br>Parent: STAI, ICS and Ottawa mood

© 2025 Luo Z et al. *JAMA Pediatrics*.

							scale at one day before surgery and on the third postoperative day<br>**Pain:** WBS at transferred to the postoperative ward
Wang et al(2021)	80	3-6 years	China	Strabismus correction surgery	Video：cartoon video (40)<br>Standard of care (40)	Preoperative	**Anxiety:** mYPAS at the time of entering the preoperative waiting area (T1) and during separation from parents (T2)<br>**Compliance:** ICC during general anaesthesia induction<br>**Parental satisfaction:** verbal score from 0 (not satisfied) to 10 (highly satisfied) at the first day after surgery
Ryu et al(2017)	69	4-10 years	Korea	Elective surgery	Virtual reality: smartphones (Galaxy S6®; Samsung, Suwon, Korea) and VR headsets (VR Gear®; Samsung) - a VR-guided tour (34)<br>Standard of care (35)	Preoperative	**Anxiety:** mYPAS in the reception area of the operating theatre 30 min after the intervention<br>**Compliance:** ICC during the induction of anaesthesia
Stewart et al(2019)	102	4-12 years	America	Outpatient surgical procedures	Game: age-appropriate games (51)<br>Midazolam, 0.3 mg/kg, po (51)	Preoperative	**Anxiety:** mYPAS-SF at baseline, at time of parental separation, and at mask induction<br>**Emergence delirium:** PAED in the PACU on emergence and 15 minutes later for signs of emergence delirium<br>**Parental satisfaction:** a seven-point Likert scale from zero (not satisfied) to

© 2025 Luo Z et al. *JAMA Pediatrics*.

							seven (very satisfied) in PACU
Cumino et al(2017)	42	4-8 years	Brazil	Minor-tomoderate elective surgical procedures	Game (21)<br>Standard of care (21)	Preoperative	**Anxiety:** mYPAS: holding area and operating room(when applying the facemask for inhalation anaesthesia induction)
Mifflin et al(2012)	99	2-10 years	Canada	Ambulatory surgery	Video: a list of age-appropriate videos (42)<br>Standard of care (47)	Preoperative	**Anxiety:** mYPAS in the holding area and at induction
Seiden et al(2014)	108	1-11 years	America	Outpatient surgical procedures	Game: age appropriate video games (57)<br>Midazolam: 0.5 mg/kg, PO (51)	Preoperative	**Anxiety:** mYPAS at parental separation and anaesthetic induction time<br>**Emergence delirium:** PAED at emergence and 15 min after PACU admission
Levay et al(2023)	99	3-5 years	America	Elective surgery	Game: tablet with age-appropriate games (52)<br>Midazolam: 0.5 mg/kg, PO (47)	Preoperative	**Anxiety:** mYPAS during mask induction<br>**Emergence delirium:** PAED at emergence from anaesthesia
Topçu et al(2023)	84	5-10 years	Turkish	Elective surgery	An interactive robot: Silverlit Macrobot (42)<br>Standard of care (42)	Preoperative	**Anxiety:** CSA at one minute before the first mobilization
Ryu et al(2018)	70	4-10 years	Korea	Elective day surgery	Virtual reality: VR game (JSC GAMES, Seoul, Korea) (35)<br>Standard of care (35)	Preoperative	**Anxiety:** mYPAS at baseline and anaesthetic induction<br>**Parental satisfaction:** NRS in the

© 2025 Luo Z et al. *JAMA Pediatrics*.

							waiting room right after the induction of anesthesia
Tuncay et al(2023)	90	4-6 years	Turkey	Circumcision surgery	Video: video animation (30)<br>Standard of care (30)<br>Enhanced control: puppet play scenario (30)	Preoperative	**Anxiety:** CAS-S at 30 min before surgery<br>**Pain:** WBS postoperative
Evren Sahin et al(2022)	286	4-10 years	Turkey	Elective surgery	Game: tablet games suitable for 4–10-year-olds (93)<br>Midazolam: 0.5 mg/kg, PO (93)<br>Standard of care (100)	Preoperative	**Anxiety:** mYPAS on the morning of the operation，20 min after administration of oral midazolam or provision of tablet computer
Buyuk et al(2021)	78	5-10 years	Turkey	Circumcision surgery	Virtual reality: "VR BOX 3.0" (40)<br>Enhanced control: Educational mode (38)	Preoperative	**Anxiety:** CAM-S before and after the surgery.<br>**Pain:** WBS postoperative
Uysal et al(2023)	80	6-12 years	Turkey	Abdominal surgery	Virtual reality: VR headset(Piranha virtual reality BOX 3.0) (40)<br>Standard of care (40)	Preoperative	**Anxiety:** STAI-C at pretransfer and posttransfer
Yaz et al(2022)	132	6-12 years	Turkey	General surgery	Virtual reality: educational or documentary animation (with VR) (88)<br>Standard of care (44)	Preoperative	**Pain:** WBS postoperative
Huang et al(2021)	116	3-7 years	China	Cardiothoracic surgery	Video: 5–10 children's favourite and educational videos (58)<br>Standard of care (58)	Postoperation	**Pain:** WBS and FLACC at immediately after the first intervention, 1 day, and 2 days after the intervention

© 2025 Luo Z et al. *JAMA Pediatrics*.

Ryu et al(2022)	67	4-10 years	South Korea	Pediatric surgery—including benign soft mass excision, inguinal hernia repair, central catheter insertion, and frenotomy	Virtual reality: a head-mounted display (34)<br>Standard of care (33)	Preoperative	**Anxiety:**<br>Child: mYPAS in the reception area at the operating theater immediately before entering<br>Parent: BAI after the child entered the OR<br>**Parental satisfaction:** a numeric rating scale (0, very dissatisfied; 50, very satisfied) a week after the operation performed
Clausen et al(2021)	60	3-6 years	Denmark	Elective minor surgery	Game: age-appropriate online games (30)<br>Standard of care (30)	Preoperative	**Anxiety:** mYPAS at baseline and during anaesthesia induction,<br>**Pain:** FLACC in PACU<br>**Emergence delirium**： PAED in PACU
Eijlers et al(2019)	191	4-12 years	Netherlands	Elective day care surgery	Virtual reality: HTC Vive (HTC Corporation, Xindian, New Taipei, Taiwan) (94)<br>Standard of care (97)	Preoperative	**Anxiety:**<br>Child: mYPAS at baseline(T1), the holding area (T2) and during induction of anaesthesia (T3); VSA at T1, T2, recovery room(T4) and at home(T5)<br>Parent: STAI at T1 and T3<br>**Pain:** FPS-r in the recovery room<br>**Emergence delirium:** PAED at T4

© 2025 Luo Z et al. *JAMA Pediatrics*.

Wu et al(2022)	99	4-12 years	China	Elective surgery	Virtual reality: head-mounted display (CV1 PRO, NOLO VR, Beijing, China) (51)<br>Video: common animation (48)	Preoperative	**Anxiety:**<br>Child: mYPAS-SF and VAS: at baseline (T1)， leaving holding area (T2), and before induction of anaesthesia (T3)<br>**Pain:** FLACC in the recovery room (T4)<br>**Compliance:** ICC at T3<br>**Emergence delirium:** PAED at T4<br>**Parental satisfaction:** a self-reported 10-point VAS at home (T5)
Lee et al(2014)	80	1-10 years	Korea	Elective surgery	Game: a behavioral intervention program (40)<br>Midazolam: 0.15 mg/kg, IV (40)	Preoperative	**Anxiety:** mYPAS at holding area, after intervrntion, and in operation room
Matthyssens et al(2020)	50	5-11 years	Belgium	Day-care surgery (general surgery, dentistry, otorhinolaryngology, urology)	Game: CliniPup® serious game (25)<br>Standard of Care (25)	Preoperative	**Anxiety:** VASa and VASa-on-child at baseline (T0), 1 week preoperatively (T1), at hospital admission (T2), before discharge (T3), 1 week postoperatively (T4), 1 month postoperatively (T5)
Mihandoust et al(2024)	16	6-18 years	America	Gastrointestinal procedure	Virtual reality: a nonimmersive interactive virtual tour in 3DVista (9)<br>Standard of Care (7)	Preoperative	**Anxiety:**<br>Child: STAI on 1-15 days before the procedure and the day of the procedure (waiting room, pre-procedure,

© 2025 Luo Z et al. *JAMA Pediatrics*.

							procedure and recovery)<br>Parent: STAI on 1-15 days before the procedure and the day of the procedure (waiting room, pre-procedure, procedure and recovery)
Bozkul et al(2024)	60	7-12 years	Turkey	Day surgery	Video: Video Based Education (30)<br>Standard of Care (30)	Preoperative	**Pain:** a numerical pain scale in the postoperative period
Franco Castanys et al(2023)	125	4-12 years	Spain	Outpatient surgery	Virtual reality: a virtual tour with real images of the OR (61)<br>Standard of Care (64)	Preoperative	**Anxiety:**<br>Child: mYPAS in the waiting room and during parents–child separation<br>Parent: STAI in the waiting room and VAS during parents–child separation<br>**Compliance:** ICC at anesthetic induction<br>**Emergence delirium:** PAED at the URPA<br>**Parental satisfaction:** Parents' Satisfaction Questionnaire
Chartrand et al(2016)	123	3-10 years	Canada	An ENT or dental same-day surgery	Video: the DVD included images of OR equipment and positive nurse–family and parent–child interactions (59)<br>Standard of Care (64)	Preoperative	**Pain:** mCHEOPS in the RR and the DCS unit<br>**Anxiety(Parent):** VAS-A immediately before entering the RR (T1), 5 minutes after entering the RR (T2) and 5 minutes after leaving the RR with their child (T3)

© 2025 Luo Z et al. *JAMA Pediatrics*.

Ryu et al(2019)	80	4-10 years	Korea	Elective surgery	Virtual reality: smartphones (Galaxy S6; Samsung, Suwon, Korea) and VR headsets (VR Gear; Samsung) (41)<br>Standard of Care (39)	Preoperative	**Anxiety:** mYPAS at baseline and at induction<br>**Emergence delirium:** PAED at the PACU
Yang et al(2016)	61	3-7 years	Korea	Tonsillectomy	Enhanced control: information on the pre- and postoperation care of tonsillectomy in the form of 10 text messages (27)<br>Standard of Care (34)	Preoperative	**Anxiety:** a modified version of an eight-item self-report instrument on the day of hospitalization and just before operation
Mete İzci et al(2024)	72	7-10 years	Turkey	Day surgery	Video: Mutlu's One-Day Hospital Adventure digital story creating through Pawton Pro+ software (36)<br>Standard of Care (36)	Preoperative	**Anxiety:** SAI at 5-7 days before Day Surgery (T1), day Surgery (T2) and postoperative 2 Weeks Later (T3)
Specht et al(2021)	106	7-18 yeras	America	General (including urologic and plastic), spine, other orthopedic, or burn (<10% total body surface area)	Virtual reality: the Nature Treks VR application (The Oculus Go headset, Irvine, CA) (33)<br>Game: educational games (iPad 5 ,Cupertino, CA) (40)	Preoperation	**Anxiety:** STAI in the preoperative period and in PACU<br>**Pain:** VAS and FACES in the preoperative period and in PACU and FLACC during device use

© 2025 Luo Z et al. *JAMA Pediatrics*.

Carbó et al(2024)	241	3-13 years	Spain	Elective surgery	Virtual reality: wearing a VR headset (Samsung Gear VR, Samsung Electronics, Seoul, South Korea) (120)<br>Standard of Care (121)	Preoperation	**Anxiety:** mYPAS at baseline and at induction<br>**Pain:** Wong-Baker Faces Pain Rating Scale was applied at entry into the recovery room<br>**Compliance:** ICC at anesthetic induction<br>**Emergence delirium:** PAED at admission to recovery room
Chen et al(2025)	90	4-7 years	China	Elective surgery	Virtual reality: VR technology (PICO 4 Vision Pro) (30)<br>Video: animated cartoon video (30)<br>Standard of Care (30)	Preoperation	**Pain:** FLACC postoperative<br>**Emergence delirium:** PAED at discharge from the PACU
Turgut et al(2024)	70	4-10 years	Turkey	Elective surgery	Virtual reality: a 360-degree video tour of the entire operating theatre area for one-and-a-half-minute using VR glasses (Oculus Go VR) (30)<br>Standard of Care (30)	Preoperation	**Anxiety:** Children's State Anxiety Scale before entering the operating theatre<br>**Pain:** Wong-Baker FACES Pain Rating Scale postoperative<br>**Parental satisfaction:** Parental satisfaction scale postoperative
Vinay et al(2024)	150	4-12 years	India	Elective surgery	Game: an age-appropriate video game (75)<br>Standard of Care (75)	Preoperation	**Anxiety:** m-YPAS scores at parental separation during transfer to the operation theatre

Note: VR: Virtual reality; mYPAS: The modified Yale Preoperative Anxiety Scale; WBFPRS: Wong-Baker Faces Pain Rating Scale; CmYPAS: The Chinese version of the modified Yale Preoperative Anxiety Scale; FPS-r: Faces Pain Scale Revised; mYPAS-SF: The modified Yale Preoperative Anxiety Scale-Short-Form; ICC: Induction

© 2025 Luo Z et al. *JAMA Pediatrics*.

Compliance Checklist; VAS: Visual Analog Scale; WBS: Wong-Baker Faces Pain Rating Scale; FACES: Wong-Baker FACES Pain Rating Scale STOA: State-Trait Operation Anxiety Inventory; PAED: Pediatric Anesthesia Emergence Delirium Scale; FLACC: Face, Legs, Activity, Cry, and Consolability; STAI: The State-Trait Anxiety Inventory; STAI-C: The child version of state-trait anxiety inventory; CSA: Children's State Anxiety; CAS-S: Child Anxiety Scale-State; CAM-S: Children's Anxiety Meter Scale; mCHEOPS: the Modified Children's Hospital of Eastern Ontario Pain Score; SAS: Self-rating Anxiety Scale; BAI: Beck Anxiety Inventory; NRS: Numeric Rating Scale; PACU: Post Anaesthesia Care Unit; DCS: day care surgery; RR: recovery room

© 2025 Luo Z et al. *JAMA Pediatrics*.

eTable S6. Assessment of risk of bias using the Cochrane Risk of Bias Tool.

Unique ID	Study ID	Randomization process	Deviations from intended interventions	Missing outcome data	Measurement of the outcome	Selection of the reported result	Overall Bias
1	Buffel et al(2019)	High	Some concerns	Low	Low	Low	High
2	Kerimoglu et al(2013)	Low	Low	Low	Low	Low	Low
3	Luo et al(2023)	Low	Some concerns	Low	Low	Low	Some concerns
4	Marechal et al(2017)	Low	Low	Low	Low	Low	Low
5	Patel et al(2006)	Some concerns	Some concerns	Low	Some concerns	Low	Some concerns
6	Hou et al(2023)	Low	Some concerns	Low	Low	Low	Some concerns
7	Baghele et al(2019)	Low	Some concerns	Low	Low	Low	Some concerns
8	Kerimaa et al(2023)	Low	Low	Low	Low	Low	Low
9	Liguori et al(2016)	Low	Low	Low	Low	Low	Low
10	Härter et al(2021)	Some concerns	High	Low	Some concerns	Low	High
11	Nair et al(2021)	Low	Low	Low	Low	Low	Low
12	Tang et al(2023)	Low	Some concerns	Low	Low	Low	Some concerns
13	Dost et al(2023)	Low	Some concerns	Low	Low	Low	Some concerns
14	Huntington et al(2017)	Low	Some concerns	Low	Low	Low	Some concerns
15	Jung et al(2021)	Low	Low	Low	Low	Low	Low
16	Kumar et al(2019)	High	Some concerns	Low	Some concerns	Low	High
17	Wang et al(2021)	Some concerns	Some concerns	Low	Some concerns	Low	Some concerns
18	Ryu et al(2017)	Low	Low	Low	Low	Low	Low
19	Stewart et al(2019)	Some concerns	Some concerns	Low	Low	Some concerns	Some concerns
20	Cumino et al(2017)	Some concerns	Some concerns	Low	Some concerns	Low	Some concerns
21	Mifflin et al(2012)	Low	Low	Low	Low	Low	Low

© 2025 Luo Z et al. *JAMA Pediatrics*.

22	Seiden et al(2014)	Low	Some concerns	Low	High	Low	High
23	Levay et al(2023)	Some concerns	High	Low	Low	Low	High
24	Topçu et al(2023)	Some concerns	Low	Low	Some concerns	Low	Some concerns
25	Ryu et al(2018)	Some concerns	Some concerns	Low	Low	Low	Some concerns
26	Tuncay et al(2023)	Some concerns	Some concerns	Low	Low	Low	Some concerns
27	Evren Sahin et al(2022)	High	High	Low	Low	Low	High
28	Buyuk et al(2021)	Low	Some concerns	Some concerns	Low	Some concerns	Some concerns
29	Uysal et al(2023)	Some concerns	Low	Low	Some concerns	Low	Some concerns
30	Yaz et al(2022)	Some concerns	Some concerns	Low	Some concerns	Some concerns	Some concerns
31	Huang et al(2021)	Low	Some concerns	Low	Some concerns	Low	Some concerns
32	Ryu et al(2022)	Some concerns	Low	Low	Low	Low	Some concerns
33	Clausen et al(2021)	Some concerns	Some concerns	Low	Some concerns	Low	Some concerns
34	Eijlers et al(2019)	Low	Low	Low	Low	Low	Low
35	Wu et al(2022)	Some concerns	High	Some concerns	Low	Low	High
36	Lee et al(2014)	Some concerns	Some concerns	Low	Low	Low	Some concerns
37	Carbó et al (2024)	Low	Some concerns	Some concerns	Some concerns	Low	Some concerns
38	Mihandoust et al(2024)	High	Some concerns	High	Low	Low	High
39	Bozkul et al(2024)	Some concerns	Some concerns	Some concerns	Low	Low	Some concerns
40	Franco Castanys et al(2023)	Some concerns	Some concerns	Some concerns	Low	Low	Some concerns
41	Chartrand et al(2016)	Low	Low	Some concerns	Low	Low	Some concerns
42	Ryu et al(2018)	Low	Low	Low	Low	Some concerns	Some concerns
43	Mete İzci et al (2024)	Low	Some concerns	Low	Some concerns	Low	Some concerns
44	Specht et al(2023)	Low	Some concerns	High	Some concerns	Low	High
45	Yang et al(2016)	Low	Some concerns	Some concerns	Some concerns	Low	Some concerns
46	Chen et al(2025)	Low	Some concerns	Low	Low	Low	Some concerns

© 2025 Luo Z et al. *JAMA Pediatrics*.

47	Turgut et al(2024)	Low	Some concerns	Low	Some concerns	Low	Some concerns
48	Carbó et al (2024)	Low	Some concerns	Some concerns	Some concerns	Low	Some concerns
49	Vinay et al(2024)	Low	Some concerns	Low	Some concerns	Low	Some concerns

© 2025 Luo Z et al. *JAMA Pediatrics*.

eTable 7. Incoherence assessments.

Notes: The study assessed the incoherence (local inconsistency for each treatment loop) by the difference in standardized mean difference (SMD) or mean difference (MD) between direct estimates and indirect estimates, and the corresponding statistical tests for the difference.

Outcome	Comparisons	Difference in SMD	Difference in MD	Z statistic	P-value
Children - preoperative anxiety	An interactive robot:Control	NA [NA, NA]	NA [NA, NA]	NA	NA
Children - preoperative anxiety	An interactive robot:Enhanced control	NA [NA, NA]	NA [NA, NA]	NA	NA
Children - preoperative anxiety	An interactive robot:Game(2D)	NA [NA, NA]	NA [NA, NA]	NA	NA
Children - preoperative anxiety	An interactive robot:Midazolam	NA [NA, NA]	NA [NA, NA]	NA	NA
Children - preoperative anxiety	An interactive robot:Video(2D)	NA [NA, NA]	NA [NA, NA]	NA	NA
Children - preoperative anxiety	An interactive robot:Virtual reality	NA [NA, NA]	NA [NA, NA]	NA	NA
Children - preoperative anxiety	Enhanced control:Control	-2.39 [-3.83, -0.95]	NA [NA, NA]	-3.25	0
Children - preoperative anxiety	Game(2D):Control	0.31 [-0.97, 1.6]	NA [NA, NA]	0.48	0.63
Children - preoperative anxiety	Midazolam:Control	-0.87 [-2.53, 0.8]	NA [NA, NA]	-1.02	0.31
Children - preoperative anxiety	Video(2D):Control	-0.29 [-1.57, 0.98]	NA [NA, NA]	-0.45	0.65
Children - preoperative anxiety	Virtual reality:Control	0.92 [-0.01, 1.86]	NA [NA, NA]	1.94	0.05
Children - preoperative anxiety	Enhanced control:Game(2D)	NA [NA, NA]	NA [NA, NA]	NA	NA
Children - preoperative anxiety	Enhanced control:Midazolam	NA [NA, NA]	NA [NA, NA]	NA	NA
Children - preoperative anxiety	Enhanced control:Video(2D)	1.05 [-0.43, 2.53]	NA [NA, NA]	1.39	0.16
Children - preoperative anxiety	Enhanced control:Virtual reality	1.21 [-0.24, 2.66]	NA [NA, NA]	1.63	0.1
Children - preoperative anxiety	Game(2D):Midazolam	-0.25 [-1.88, 1.38]	NA [NA, NA]	-0.3	0.77
Children - preoperative anxiety	Game(2D):Video(2D)	NA [NA, NA]	NA [NA, NA]	NA	NA
Children - preoperative anxiety	Game(2D):Virtual reality	0.05 [-1.61, 1.71]	NA [NA, NA]	0.06	0.95
Children - preoperative anxiety	Midazolam:Video(2D)	NA [NA, NA]	NA [NA, NA]	NA	NA
Children - preoperative anxiety	Midazolam:Virtual reality	0.02 [-1.72, 1.76]	NA [NA, NA]	0.02	0.98
Children - preoperative anxiety	Video(2D):Virtual reality	1.21 [-0.41, 2.83]	NA [NA, NA]	1.47	0.14

Children - postoperative pain	Enhanced control:Control	-0.79 [-2.75, 1.17]	NA [NA, NA]	-0.79	0.43
Children - postoperative pain	Game(2D):Control	-0.19 [-1.92, 1.55]	NA [NA, NA]	-0.21	0.83
Children - postoperative pain	Video(2D):Control	-0.71 [-2.18, 0.76]	NA [NA, NA]	-0.95	0.34
Children - postoperative pain	Virtual reality:Control	0.2 [-0.92, 1.31]	NA [NA, NA]	0.34	0.73
Children - postoperative pain	Enhanced control:Game(2D)	NA [NA, NA]	NA [NA, NA]	NA	NA
Children - postoperative pain	Enhanced control:Video(2D)	-0.41 [-2.43, 1.6]	NA [NA, NA]	-0.4	0.69
Children - postoperative pain	Enhanced control:Virtual reality	0.79 [-1.17, 2.74]	NA [NA, NA]	0.79	0.43
Children - postoperative pain	Game(2D):Video(2D)	NA [NA, NA]	NA [NA, NA]	NA	NA
Children - postoperative pain	Game(2D):Virtual reality	0.19 [-1.55, 1.92]	NA [NA, NA]	0.21	0.83
Children - postoperative pain	Video(2D):Virtual reality	0.64 [-0.63, 1.92]	NA [NA, NA]	0.99	0.32
Children - emergence delirium	Game(2D):Control	NA [NA, NA]	NA [NA, NA]	NA	NA
Children - emergence delirium	Midazolam:Control	NA [NA, NA]	NA [NA, NA]	NA	NA
Children - emergence delirium	Video(2D):Control	NA [NA, NA]	-6.54 [-13.76, 0.68]	-1.78	0.08
Children - emergence delirium	Virtual reality:Control	NA [NA, NA]	0.14 [-8.25, 8.53]	0.03	0.97
Children - emergence delirium	Game(2D):Midazolam	NA [NA, NA]	NA [NA, NA]	NA	NA
Children - emergence delirium	Game(2D):Video(2D)	NA [NA, NA]	NA [NA, NA]	NA	NA
Children - emergence delirium	Game(2D):Virtual reality	NA [NA, NA]	NA [NA, NA]	NA	NA
Children - emergence delirium	Midazolam:Video(2D)	NA [NA, NA]	NA [NA, NA]	NA	NA
Children - emergence delirium	Midazolam:Virtual reality	NA [NA, NA]	NA [NA, NA]	NA	NA
Children - emergence delirium	Video(2D):Virtual reality	NA [NA, NA]	6.35 [-0.96, 13.66]	1.7	0.09
Children - induction compliance	Video(2D):Control	NA [NA, NA]	-0.45 [-2.2, 1.29]	-0.51	0.61
Children - induction compliance	Virtual reality:Control	NA [NA, NA]	0.45 [-1.29, 2.2]	0.51	0.61
Children - induction compliance	Video(2D):Virtual reality	NA [NA, NA]	0.45 [-1.29, 2.2]	0.51	0.61
Parents - preoperative anxiety	Enhanced control:Control	NA [NA, NA]	NA [NA, NA]	NA	NA
Parents - preoperative anxiety	Game(2D):Control	NA [NA, NA]	NA [NA, NA]	NA	NA

© 2025 Luo Z et al. *JAMA Pediatrics*.

Parents - preoperative anxiety	Midazolam:Control	NA [NA, NA]	NA [NA, NA]	NA	NA
Parents - preoperative anxiety	Video(2D):Control	NA [NA, NA]	NA [NA, NA]	NA	NA
Parents - preoperative anxiety	Virtual reality:Control	NA [NA, NA]	NA [NA, NA]	NA	NA
Parents - preoperative anxiety	Enhanced control:Game(2D)	NA [NA, NA]	NA [NA, NA]	NA	NA
Parents - preoperative anxiety	Enhanced control:Midazolam	NA [NA, NA]	NA [NA, NA]	NA	NA
Parents - preoperative anxiety	Enhanced control:Video(2D)	NA [NA, NA]	NA [NA, NA]	NA	NA
Parents - preoperative anxiety	Enhanced control:Virtual reality	NA [NA, NA]	NA [NA, NA]	NA	NA
Parents - preoperative anxiety	Game(2D):Midazolam	NA [NA, NA]	NA [NA, NA]	NA	NA
Parents - preoperative anxiety	Game(2D):Video(2D)	NA [NA, NA]	NA [NA, NA]	NA	NA
Parents - preoperative anxiety	Game(2D):Virtual reality	NA [NA, NA]	NA [NA, NA]	NA	NA
Parents - preoperative anxiety	Midazolam:Video(2D)	NA [NA, NA]	NA [NA, NA]	NA	NA
Parents - preoperative anxiety	Midazolam:Virtual reality	NA [NA, NA]	NA [NA, NA]	NA	NA
Parents - preoperative anxiety	Video(2D):Virtual reality	NA [NA, NA]	NA [NA, NA]	NA	NA
Parents - postoperative satisfaction	Enhanced control:Control	NA [NA, NA]	NA [NA, NA]	NA	NA
Parents - postoperative satisfaction	Game(2D):Control	NA [NA, NA]	NA [NA, NA]	NA	NA
Parents - postoperative satisfaction	Midazolam:Control	NA [NA, NA]	NA [NA, NA]	NA	NA
Parents - postoperative satisfaction	Video(2D):Control	0.27 [-0.77, 1.31]	NA [NA, NA]	0.51	0.61
Parents - postoperative satisfaction	Virtual reality:Control	-0.27 [-1.31, 0.77]	NA [NA, NA]	-0.51	0.61
Parents - postoperative satisfaction	Enhanced control:Game(2D)	NA [NA, NA]	NA [NA, NA]	NA	NA
Parents - postoperative satisfaction	Enhanced control:Midazolam	NA [NA, NA]	NA [NA, NA]	NA	NA
Parents - postoperative satisfaction	Enhanced control:Video(2D)	NA [NA, NA]	NA [NA, NA]	NA	NA
Parents - postoperative satisfaction	Enhanced control:Virtual reality	NA [NA, NA]	NA [NA, NA]	NA	NA
Parents - postoperative satisfaction	Game(2D):Midazolam	NA [NA, NA]	NA [NA, NA]	NA	NA
Parents - postoperative satisfaction	Game(2D):Video(2D)	NA [NA, NA]	NA [NA, NA]	NA	NA
Parents - postoperative satisfaction	Game(2D):Virtual reality	NA [NA, NA]	NA [NA, NA]	NA	NA

© 2025 Luo Z et al. *JAMA Pediatrics*.

Parents - postoperative satisfaction	Midazolam:Video(2D)	NA [NA, NA]	NA [NA, NA]	NA	NA
Parents - postoperative satisfaction	Midazolam:Virtual reality	NA [NA, NA]	NA [NA, NA]	NA	NA
Parents - postoperative satisfaction	Video(2D):Virtual reality	-0.27 [-1.31, 0.77]	NA [NA, NA]	-0.51	0.61
Subgroup Analysis					
Children - Anxiety (Day or Outpatient Surgery)	Enhanced control:Control	NA [NA, NA]	NA [NA, NA]	NA	NA
Children - Anxiety (Day or Outpatient Surgery)	Game(2D):Control	-0.01 [-1.7, 1.69]	NA [NA, NA]	-0.01	0.99
Children - Anxiety (Day or Outpatient Surgery)	Midazolam:Control	NA [NA, NA]	NA [NA, NA]	NA	NA
Children - Anxiety (Day or Outpatient Surgery)	Video(2D):Control	-1.18 [-3.11, 0.76]	NA [NA, NA]	-1.19	0.23
Children - Anxiety (Day or Outpatient Surgery)	Virtual reality:Control	0.64 [-0.78, 2.05]	NA [NA, NA]	0.88	0.38
Children - Anxiety (Day or Outpatient Surgery)	Enhanced control:Game(2D)	NA [NA, NA]	NA [NA, NA]	NA	NA
Children - Anxiety (Day or Outpatient Surgery)	Enhanced control:Midazolam	NA [NA, NA]	NA [NA, NA]	NA	NA
Children - Anxiety (Day or Outpatient Surgery)	Enhanced control:Video(2D)	-1.18 [-3.11, 0.76]	NA [NA, NA]	-1.19	0.23
Children - Anxiety (Day or Outpatient Surgery)	Enhanced control:Virtual reality	1.18 [-0.76, 3.11]	NA [NA, NA]	1.19	0.23
Children - Anxiety (Day or Outpatient Surgery)	Game(2D):Midazolam	0.01 [-1.69, 1.7]	NA [NA, NA]	0.01	0.99
Children - Anxiety (Day or Outpatient Surgery)	Game(2D):Video(2D)	NA [NA, NA]	NA [NA, NA]	NA	NA
Children - Anxiety (Day or Outpatient Surgery)	Game(2D):Virtual reality	NA [NA, NA]	NA [NA, NA]	NA	NA
Children - Anxiety (Day or Outpatient Surgery)	Midazolam:Video(2D)	NA [NA, NA]	NA [NA, NA]	NA	NA
Children - Anxiety (Day or Outpatient Surgery)	Midazolam:Virtual reality	0.01 [-1.69, 1.7]	NA [NA, NA]	0.01	0.99
Children - Anxiety (Day or Outpatient Surgery)	Video(2D):Virtual reality	NA [NA, NA]	NA [NA, NA]	NA	NA
Children - Anxiety (Elective Surgery)	An interactive robot:Control	NA [NA, NA]	NA [NA, NA]	NA	NA
Children - Anxiety (Elective Surgery)	An interactive robot:Enhanced control	NA [NA, NA]	NA [NA, NA]	NA	NA
Children - Anxiety (Elective Surgery)	An interactive robot:Game(2D)	NA [NA, NA]	NA [NA, NA]	NA	NA
Children - Anxiety (Elective Surgery)	An interactive robot:Midazolam	NA [NA, NA]	NA [NA, NA]	NA	NA
Children - Anxiety (Elective Surgery)	An interactive robot:Video(2D)	NA [NA, NA]	NA [NA, NA]	NA	NA
Children - Anxiety (Elective Surgery)	An interactive robot:Virtual reality	NA [NA, NA]	NA [NA, NA]	NA	NA

© 2025 Luo Z et al. *JAMA Pediatrics*.

Children - Anxiety (Elective Surgery)	Enhanced control:Control	-2.45 [-4.09, -0.8]	NA [NA, NA]	-2.91	0
Children - Anxiety (Elective Surgery)	Game(2D):Control	0.17 [-1.33, 1.67]	NA [NA, NA]	0.22	0.82
Children - Anxiety (Elective Surgery)	Midazolam:Control	-0.55 [-2.37, 1.26]	NA [NA, NA]	-0.59	0.55
Children - Anxiety (Elective Surgery)	Video(2D):Control	-0.47 [-1.91, 0.97]	NA [NA, NA]	-0.64	0.52
Children - Anxiety (Elective Surgery)	Virtual reality:Control	0.96 [-0.07, 1.99]	NA [NA, NA]	1.83	0.07
Children - Anxiety (Elective Surgery)	Enhanced control:Game(2D)	NA [NA, NA]	NA [NA, NA]	NA	NA
Children - Anxiety (Elective Surgery)	Enhanced control:Midazolam	NA [NA, NA]	NA [NA, NA]	NA	NA
Children - Anxiety (Elective Surgery)	Enhanced control:Video(2D)	1.62 [-0.18, 3.43]	NA [NA, NA]	1.77	0.08
Children - Anxiety (Elective Surgery)	Enhanced control:Virtual reality	1.48 [-0.26, 3.23]	NA [NA, NA]	1.66	0.1
Children - Anxiety (Elective Surgery)	Game(2D):Midazolam	0.02 [-2.86, 2.9]	NA [NA, NA]	0.02	0.99
Children - Anxiety (Elective Surgery)	Game(2D):Video(2D)	NA [NA, NA]	NA [NA, NA]	NA	NA
Children - Anxiety (Elective Surgery)	Game(2D):Virtual reality	0.25 [-1.37, 1.87]	NA [NA, NA]	0.3	0.77
Children - Anxiety (Elective Surgery)	Midazolam:Video(2D)	NA [NA, NA]	NA [NA, NA]	NA	NA
Children - Anxiety (Elective Surgery)	Midazolam:Virtual reality	NA [NA, NA]	NA [NA, NA]	NA	NA
Children - Anxiety (Elective Surgery)	Video(2D):Virtual reality	0.77 [-0.77, 2.31]	NA [NA, NA]	0.98	0.33
Children - Pain (Day or Outpatient Surgery)	Game(2D):Control	NA [NA, NA]	NA [NA, NA]	NA	NA
Children - Pain (Day or Outpatient Surgery)	Video(2D):Control	NA [NA, NA]	NA [NA, NA]	NA	NA
Children - Pain (Day or Outpatient Surgery)	Virtual reality:Control	NA [NA, NA]	NA [NA, NA]	NA	NA
Children - Pain (Day or Outpatient Surgery)	Game(2D):Video(2D)	NA [NA, NA]	NA [NA, NA]	NA	NA
Children - Pain (Day or Outpatient Surgery)	Game(2D):Virtual reality	NA [NA, NA]	NA [NA, NA]	NA	NA
Children - Pain (Day or Outpatient Surgery)	Video(2D):Virtual reality	NA [NA, NA]	NA [NA, NA]	NA	NA
Children - Pain (Elective Surgery)	Enhanced control:Control	-0.68 [-2.54, 1.19]	NA [NA, NA]	-0.71	0.48
Children - Pain (Elective Surgery)	Game(2D):Control	-0.27 [-2, 1.46]	NA [NA, NA]	-0.31	0.76
Children - Pain (Elective Surgery)	Video(2D):Control	-0.28 [-1.75, 1.2]	NA [NA, NA]	-0.37	0.72
Children - Pain (Elective Surgery)	Virtual reality:Control	-0.07 [-1.21, 1.07]	NA [NA, NA]	-0.12	0.91

© 2025 Luo Z et al. *JAMA Pediatrics*.

Children - Pain (Elective Surgery)	Enhanced control:Game(2D)	NA [NA, NA]	NA [NA, NA]	NA	NA
Children - Pain (Elective Surgery)	Enhanced control:Video(2D)	-0.21 [-2.15, 1.73]	NA [NA, NA]	-0.21	0.83
Children - Pain (Elective Surgery)	Enhanced control:Virtual reality	0.57 [-1.28, 2.42]	NA [NA, NA]	0.6	0.55
Children - Pain (Elective Surgery)	Game(2D):Video(2D)	NA [NA, NA]	NA [NA, NA]	NA	NA
Children - Pain (Elective Surgery)	Game(2D):Virtual reality	0.27 [-1.46, 2]	NA [NA, NA]	0.31	0.76
Children - Pain (Elective Surgery)	Video(2D):Virtual reality	0.32 [-0.99, 1.63]	NA [NA, NA]	0.48	0.63

© 2025 Luo Z et al. *JAMA Pediatrics*.

eTable 8. Heterogeneity (Inconsistency) assessments.

Notes: The study assess the between-study heterogeneity for all comparisons in each outcome by several pairwise random-effect meta-analyses. We evaluated the potential heterogeneity if the P-value for Q-statistic is less than 0.05, or the I-squared is more than 50%. We then judged whether there is a clinical heterogeneity supervised by the clinical experts.

Abbreviations: k, number of studies providing direct evidence; I, I statistic; tau, standard deviation of the random-effect distribution.

Outcome	Comparisons	k	Q statistic	P-value	tau^2	I^2
Children - preoperative anxiety	An interactive robot:Control	1	0	NA	NA	NA
Children - preoperative anxiety	Enhanced control:Control	2	5.097	0.038	0.441	0.804
Children - preoperative anxiety	Game(2D):Control	7	79.836	0.001	0.818	0.925
Children - preoperative anxiety	Midazolam:Control	1	0	NA	NA	NA
Children - preoperative anxiety	Video(2D):Control	11	123.242	0.001	0.631	0.919
Children - preoperative anxiety	Virtual reality:Control	11	92.755	0.001	0.388	0.892
Children - preoperative anxiety	Enhanced control:Video(2D)	2	3.312	0.091	0.147	0.698
Children - preoperative anxiety	Enhanced control:Virtual reality	2	0.09	0.764	0	0
Children - preoperative anxiety	Game(2D):Midazolam	7	50.047	0.001	0.287	0.88
Children - preoperative anxiety	Game(2D):Virtual reality	1	0	NA	NA	NA
Children - preoperative anxiety	Midazolam:Virtual reality	1	0	NA	NA	NA
Children - preoperative anxiety	Video(2D):Virtual reality	1	0	NA	NA	NA
Children - postoperative pain	Enhanced control:Control	1	0	NA	NA	NA
Children - postoperative pain	Game(2D):Control	3	15.408	0.001	0.764	0.87
Children - postoperative pain	Video(2D):Control	7	13.96	0.035	0.074	0.57
Children - postoperative pain	Virtual reality:Control	6	115.589	0.001	0.824	0.957
Children - postoperative pain	Enhanced control:Video(2D)	1	0	NA	NA	NA
Children - postoperative pain	Enhanced control:Virtual reality	1	0	NA	NA	NA
Children - postoperative pain	Game(2D):Virtual reality	1	0	NA	NA	NA

© 2025 Luo Z et al. *JAMA Pediatrics*.

Children - postoperative pain	Video(2D):Virtual reality	2	29.662	0.001	1.838	0.966
Children - emergence delirium	Game(2D):Control	1	0	NA	NA	NA
Children - emergence delirium	Video(2D):Control	2	9.345	0.101	7.669	0.893
Children - emergence delirium	Virtual reality:Control	5	124.653	0.001	11.354	0.968
Children - emergence delirium	Game(2D):Midazolam	3	12.396	0.002	4.367	0.839
Children - emergence delirium	Video(2D):Virtual reality	2	21.808	0.001	7.352	0.954
Children - induction compliance	Video(2D):Control	3	17.76	0.001	1.653	0.887
Children - induction compliance	Virtual reality:Control	3	24.74	0.001	0.492	0.919
Children - induction compliance	Video(2D):Virtual reality	1	0	NA	NA	NA
Parents - preoperative anxiety	Game(2D):Control	2	4.462	0.035	0.574	0.776
Parents - preoperative anxiety	Video(2D):Control	2	0.113	0.737	0	0
Parents - preoperative anxiety	Virtual reality:Control	5	1.193	0.879	0	0
Parents - preoperative anxiety	Enhanced control:Video(2D)	1	0	NA	NA	NA
Parents - preoperative anxiety	Game(2D):Midazolam	1	0	NA	NA	NA
Parents - postoperative satisfaction	Game(2D):Control	1	0	NA	NA	NA
Parents - postoperative satisfaction	Video(2D):Control	1	0	NA	NA	NA
Parents - postoperative satisfaction	Virtual reality:Control	4	11.228	0.011	0.14	0.733
Parents - postoperative satisfaction	Enhanced control:Virtual reality	1	0	NA	NA	NA
Parents - postoperative satisfaction	Game(2D):Midazolam	2	1.045	0.307	0.002	0.043
Parents - postoperative satisfaction	Video(2D):Virtual reality	1	0	NA	NA	NA
Subgroup Analysis						
Children - Anxiety (Day or Outpatient Surgery)	Game(2D):Control	3	11.108	0.004	0.41	0.82
Children - Anxiety (Day or Outpatient Surgery)	Video(2D):Control	4	25.669	0.001	0.483	0.883
Children - Anxiety (Day or Outpatient Surgery)	Virtual reality:Control	2	9.412	0.002	0.229	0.894
Children - Anxiety (Day or Outpatient Surgery)	Enhanced control:Video(2D)	1	0	NA	NA	NA

© 2025 Luo Z et al. *JAMA Pediatrics*.

Children - Anxiety (Day or Outpatient Surgery)	Enhanced control:Virtual reality	1	0	NA	NA	NA
Children - Anxiety (Day or Outpatient Surgery)	Game(2D):Midazolam	4	21.637	0.001	0.266	0.861
Children - Anxiety (Day or Outpatient Surgery)	Midazolam:Virtual reality	1	0	NA	NA	NA
Children - Anxiety (Elective Surgery)	An interactive robot:Control	1	0	NA	NA	NA
Children - Anxiety (Elective Surgery)	Enhanced control:Control	2	5.097	0.038	0.441	0.804
Children - Anxiety (Elective Surgery)	Game(2D):Control	4	49.301	0.001	1.077	0.939
Children - Anxiety (Elective Surgery)	Midazolam:Control	1	0	NA	NA	NA
Children - Anxiety (Elective Surgery)	Video(2D):Control	7	64.692	0.001	0.515	0.907
Children - Anxiety (Elective Surgery)	Virtual reality:Control	9	34.058	0.001	0.186	0.765
Children - Anxiety (Elective Surgery)	Enhanced control:Video(2D)	1	0	NA	NA	NA
Children - Anxiety (Elective Surgery)	Enhanced control:Virtual reality	1	0	NA	NA	NA
Children - Anxiety (Elective Surgery)	Game(2D):Midazolam	3	21.898	0.001	0.369	0.909
Children - Anxiety (Elective Surgery)	Game(2D):Virtual reality	1	0	NA	NA	NA
Children - Anxiety (Elective Surgery)	Video(2D):Virtual reality	1	0	NA	NA	NA
Children - Pain (Day or Outpatient Surgery)	Game(2D):Control	1	0	NA	NA	NA
Children - Pain (Day or Outpatient Surgery)	Video(2D):Control	3	8.18	0.017	0.191	0.756
Children - Pain (Day or Outpatient Surgery)	Virtual reality:Control	1	0	NA	NA	NA
Children - Pain (Elective Surgery)	Enhanced control:Control	1	0	NA	NA	NA
Children - Pain (Elective Surgery)	Game(2D):Control	2	12.598	0.001	1.002	0.921
Children - Pain (Elective Surgery)	Video(2D):Control	4	5.03	0.231	0.036	0.404
Children - Pain (Elective Surgery)	Virtual reality:Control	5	65.911	0.001	0.691	0.939
Children - Pain (Elective Surgery)	Enhanced control:Video(2D)	1	0	NA	NA	NA
Children - Pain (Elective Surgery)	Enhanced control:Virtual reality	1	0	NA	NA	NA
Children - Pain (Elective Surgery)	Game(2D):Virtual reality	1	0	NA	NA	NA
Children - Pain (Elective Surgery)	Video(2D):Virtual reality	2	29.662	0.001	1.838	0.966

© 2025 Luo Z et al. *JAMA Pediatrics*.

eTable 9. Certainty of evidence for direct, indirect and network estimates.

Notes: The study followed GRADE guidance to assess the certainty of evidence. The assessment starts from high certainty for direct evidence and then potentially is rated down for risk of bias (D1), indirectness (D2), heterogeneity (D3) and local publication bias (D4). The evidence from indirect comparisons starts as a certainty that consists of the certainty from direct evidence that contributes to the indirect evidence, and then is rated down for intransitivity (D5) if necessary. The evidence from network comparisons starts as a certainty that consists of the certainty of direct evidence and indirect evidence weighted by the proportion of contribution (Prop), and then is rated down for incoherence (D6) and imprecision (D7).

Abbreviations: D1, risk of bias; D2, indirectness; D3, heterogeneity (inconsistency); D4, publication bias; D5, intransitivity; D6, incoherence; D7, imprecision; k, no. of studies; NMA, network meta-analysis; Prop, proportion of contribution of direct estimates to network estimates

**Children - preoperative anxiety**																
Comparisons	k	Direct estimates	D1	D2	D3	D4	Direct Certainty	Indirect estimates	Initial Indirect Certainty	D5	NMA estimates	Prop	Initial NMA Certainty	D6	D7	NMA Certainty
An interactive robot:Control	1	-0.63 [-2.14, 0.88]	0	0	0	0	High	NA [NA, NA]	NA	0	-0.63 [-2.14, 0.88]	1	High	0	-1	Moderate
An interactive robot:Enhanced control	0	NA [NA, NA]	NA	NA	NA	NA	NA	0.2 [-1.47, 1.87]	Low	0	0.2 [-1.47, 1.87]	0	Low	0	-1	Very low
An interactive robot:Game(2D)	0	NA [NA, NA]	NA	NA	NA	NA	NA	0.38 [-1.22, 1.99]	Low	0	0.38 [-1.22, 1.99]	0	Low	0	-1	Very low
An interactive robot:Midazolam	0	NA [NA, NA]	NA	NA	NA	NA	NA	-0.05 [-1.7, 1.61]	Low	0	-0.05 [-1.7, 1.61]	0	Low	0	-1	Very low
An interactive robot:Video(2D)	0	NA [NA, NA]	NA	NA	NA	NA	NA	0.44 [-1.13, 2.02]	Moderate	0	0.44 [-1.13, 2.02]	0	Moderate	0	-1	Low
An interactive robot:Virtual reality	0	NA [NA, NA]	NA	NA	NA	NA	NA	0.51 [-1.06, 2.07]	Moderate	0	0.51 [-1.06, 2.07]	0	Moderate	0	-1	Low
Enhanced control:Control	2	-2.27 [-3.39,	0	0	-1	0	Moderate	0.11 [-0.79,	Moderate	0	-0.83 [-1.53,	0.4	Moderate	-1	0	Low

© 2025 Luo Z et al. *JAMA Pediatrics*.

		-1.15]						1.02]			-0.13]					
Game(2D):Control	7	-0.95 [-1.55, -0.35]	-1	0	-1	0	Low	-1.26 [-2.4, -0.12]	Low	0	-1.02 [-1.54, -0.49]	0.78	Low	0	0	Low
Midazolam:Control	1	-1.27 [-2.75, 0.21]	-1	0	0	0	Moderate	-0.4 [-1.17, 0.36]	Low	0	-0.59 [-1.26, 0.09]	0.21	Moderate	0	-1	Low
Video(2D):Control	11	-1.11 [-1.57, -0.65]	0	0	-1	0	Moderate	-0.82 [-2.01, 0.37]	Moderate	0	-1.08 [-1.51, -0.65]	0.87	Moderate	0	0	Moderate
Virtual reality:Control	11	-0.91 [-1.38, -0.45]	0	0	-1	0	Moderate	-1.84 [-2.65, -1.03]	Moderate	0	-1.14 [-1.54, -0.74]	0.75	Moderate	0	0	Moderate
Enhanced control:Game(2D)	0	NA [NA, NA]	NA	NA	NA	NA	NA	0.19 [-0.68, 1.05]	Low	0	0.19 [-0.68, 1.05]	0	Low	0	-1	Very low
Enhanced control:Midazolam	0	NA [NA, NA]	NA	NA	NA	NA	NA	-0.24 [-1.2, 0.71]	Low	0	-0.24 [-1.2, 0.71]	0	Low	0	-1	Very low
Enhanced control:Video(2D)	2	0.81 [-0.28, 1.89]	0	0	0	0	High	-0.24 [-1.25, 0.77]	Moderate	0	0.25 [-0.49, 0.98]	0.47	High	0	-1	Moderate
Enhanced control:Virtual reality	2	0.98 [-0.1, 2.06]	0	0	0	0	High	-0.23 [-1.19, 0.74]	Moderate	0	0.31 [-0.41, 1.03]	0.45	High	0	-1	Moderate
Game(2D):Midazolam	7	-0.46 [-1.03, 0.11]	-1	0	-1	0	Low	-0.21 [-1.74, 1.32]	Low	0	-0.43 [-0.96, 0.1]	0.88	Low	0	-1	Very low
Game(2D):Video(2D)	0	NA [NA, NA]	NA	NA	NA	NA	NA	0.06 [-0.62, 0.73]	Moderate	0	0.06 [-0.62, 0.73]	0	Moderate	0	-1	Low
Game(2D):Virtual reality	1	0.17 [-1.35, 1.69]	-1	0	0	0	Moderate	0.11 [-0.55, 0.78]	Moderate	0	0.12 [-0.49, 0.73]	0.16	Moderate	0	-1	Low
Midazolam:Video(2D)	0	NA [NA, NA]	NA	NA	NA	NA	NA	0.49 [-0.31, 1.28]	Moderate	0	0.49 [-0.31, 1.28]	0	Moderate	0	-1	Low
Midazolam:Virtual reality	1	0.57 [-0.96,	0	0	0	0	High	0.55 [-0.28,	Moderate	0	0.55 [-0.17,	0.23	High	0	-1	Moderate

© 2025 Luo Z et al. *JAMA Pediatrics*.

		2.1]						1.38]			1.28]					
Video(2D):Virtual reality	1	1.12 [-0.39, 2.62]	-1	0	0	0	Moderate	-0.1 [-0.69, 0.49]	Moderate	0	0.06 [-0.49, 0.62]	0.13	Moderate	0	-1	Low
**Children - postoperative pain**																
Comparisons	k	Direct	D1	D2	D3	D4	Direct Certainty	Indirect estimates	Initial Indirect Certainty	D5	NMA estimates	Prop	Initial NMA Certainty	D6	D7	NMA Certainty
Enhanced control:Control	1	-0.68 [-2.11, 0.76]	0	0	0	0	High	0.12 [-1.22, 1.45]	Moderate	0	-0.25 [-1.23, 0.72]	0.46	High	0	-1	Moderate
Game(2D):Control	3	-0.92 [-1.79, -0.05]	-1	0	-1	0	Low	-0.73 [-2.24, 0.77]	Low	0	-0.87 [-1.62, -0.12]	0.75	Low	0	0	Low
Video(2D):Control	7	-0.65 [-1.19, -0.11]	0	0	-1	0	Moderate	0.06 [-1.31, 1.43]	Moderate	0	-0.56 [-1.06, -0.06]	0.87	Moderate	0	0	Moderate
Virtual reality:Control	6	-1.04 [-1.61, -0.46]	0	0	-1	0	Moderate	-1.23 [-2.19, -0.27]	Moderate	0	-1.09 [-1.58, -0.59]	0.74	Moderate	0	0	Moderate
Enhanced control:Game(2D)	0	NA [NA, NA]	NA	NA	NA	NA	NA	0.62 [-0.58, 1.83]	Low	0	0.62 [-0.58, 1.83]	0	Low	0	-1	Very low
Enhanced control:Video(2D)	1	0.09 [-1.33, 1.52]	0	0	0	0	High	0.51 [-0.9, 1.92]	Moderate	0	0.3 [-0.7, 1.31]	0.49	High	0	-1	Moderate
Enhanced control:Virtual reality	1	1.25 [-0.17, 2.68]	0	0	0	0	High	0.46 [-0.88, 1.81]	Moderate	0	0.84 [-0.14, 1.81]	0.47	High	0	-1	Moderate
Game(2D):Video(2D)	0	NA [NA,	NA	NA	NA	NA	NA	-0.32 [-1.2,	Moderate	0	-0.32 [-1.2,	0	Moderate	0	-1	Low

© 2025 Luo Z et al. *JAMA Pediatrics*.

		NA]						0.57]			0.57]					
Game(2D):Virtual reality	1	0.34 [-1.08, 1.75]	-1	0	0	0	Moderate	0.15 [-0.86, 1.16]	Moderate	0	0.21 [-0.61, 1.04]	0.34	Moderate	0	-1	Low
Video(2D):Virtual reality	2	0.93 [-0.08, 1.94]	-1	0	-1	0	Low	0.29 [-0.49, 1.07]	Low	0	0.53 [-0.09, 1.15]	0.37	Low	0	-1	Very low

Children - emergence delirium

Comparisons	k	Direct	D1	D2	D3	D4	Direct Certainty	Indirect estimates	Initial Indirect Certainty	D5	NMA estimates	Prop	Initial NMA Certainty	D6	D7	NMA Certainty
Game(2D):Control	1	0.4 [-5.15, 5.95]	0	0	0	0	High	NA [NA, NA]	NA	0	0.4 [-5.15, 5.95]	1	High	0	-1	Moderate
Midazolam:Control	0	NA [NA, NA]	NA	NA	NA	NA	NA	2.67 [-3.82, 9.16]	Moderate	0	2.67 [-3.82, 9.16]	0	Moderate	0	-1	Low
Video(2D):Control	2	-3.5 [-7.62, 0.62]	0	0	0	0	High	3.05 [-2.88, 8.97]	Moderate	0	-1.37 [-4.75, 2.02]	0.67	High	0	-1	Moderate
Virtual reality:Control	5	-2.37 [-4.94, 0.2]	0	0	-1	0	Moderate	-2.51 [-10.49, 5.48]	Moderate	0	-2.38 [-4.83, 0.07]	0.91	Moderate	0	-1	Low
Game(2D):Midazolam	3	-2.27 [-5.63, 1.09]	-1	0	-1	0	Low	NA [NA, NA]	NA	0	-2.27 [-5.63, 1.09]	1	Low	0	0	Low
Game(2D):Video(2D)	0	NA [NA, NA]	NA	NA	NA	NA	NA	1.77 [-4.73, 8.26]	Moderate	0	1.77 [-4.73, 8.26]	0	Moderate	0	-1	Low
Game(2D):Virtual reality	0	NA [NA, NA]	NA	NA	NA	NA	NA	2.78 [-3.28, 8.85]	Moderate	0	2.78 [-3.28, 8.85]	0	Moderate	0	-1	Low
Midazolam:Video(2D)	0	NA [NA,	NA	NA	NA	NA	NA	4.03 [-3.28,	Moderate	0	4.03 [-3.28,	0	Moderate	0	-1	Low

© 2025 Luo Z et al. *JAMA Pediatrics*.

Comparisons	k	Direct	D1	D2	D3	D4	Direct Certainty	Indirect estimates	Initial Indirect Certainty	D5	NMA estimates	Prop	Initial NMA Certainty	D6	D7	NMA Certainty
		NA]						11.35]			11.35]					
Midazolam:Virtual reality	0	NA [NA, NA]	NA	NA	NA	NA	NA	5.05 [-1.88, 11.98]	Moderate	0	5.05 [-1.88, 11.98]	0	Moderate	0	-1	Low
Video(2D):Virtual reality	2	2.91 [-1.08, 6.9]	-1	0	-1	0	Low	-3.44 [-9.57, 2.68]	Low	0	1.02 [-2.33, 4.36]	0.7	Low	0	-1	Very low
**Children - induction compliance**																
Comparisons	k	Direct	D1	D2	D3	D4	Direct Certainty	Indirect estimates	Initial Indirect Certainty	D5	NMA estimates	Prop	Initial NMA Certainty	D6	D7	NMA Certainty
Video(2D):Control	3	-0.65 [-1.54, 0.25]	0	0	-1	0	Moderate	-0.19 [-1.69, 1.3]	Moderate	0	-0.53 [-1.3, 0.24]	0.73	Moderate	0	-1	Low
Virtual reality:Control	3	-0.84 [-1.61, -0.07]	0	0	-1	0	Moderate	-1.29 [-2.86, 0.27]	Moderate	0	-0.93 [-1.62, -0.24]	0.8	Moderate	0	0	Moderate
Video(2D):Virtual reality	1	0.65 [-0.64, 1.93]	-1	0	0	0	Moderate	0.19 [-0.99, 1.38]	Moderate	0	0.4 [-0.47, 1.27]	0.46	Moderate	0	-1	Low
**Parents - preoperative anxiety**																
Comparisons	k	Direct	D1	D2	D3	D4	Direct Certainty	Indirect estimates	Initial Indirect Certainty	D5	NMA estimates	Prop	Initial NMA Certainty	D6	D7	NMA Certainty
Enhanced control:Control	0	NA	NA	NA	NA	NA	NA	0.34 [-0.22, 0.89]	Moderate	0	0.34 [-0.22, 0.89]	0	Moderate	0	-1	Low
Game(2D):Control	2	-1.59 [-2.12, -1.06]	-1	0	0	0	Moderate	NA	Moderate	0	-1.59 [-2.12, -1.06]	1	Moderate	0	0	Moderate
Midazolam:Control	0	NA	NA	NA	NA	NA	NA	-1.46 [-2.1, -0.82]	Moderate	0	-1.46 [-2.1, -0.82]	0	Moderate	0	0	Moderate

© 2025 Luo Z et al. *JAMA Pediatrics*.

Video(2D):Control	2	-0.21 [-0.54, 0.12]	0	0	0	0	High	NA	NA	0	-0.21 [-0.54, 0.12]	1	High	0	-1	Moderate
Virtual reality:Control	5	-0.03 [-0.21, 0.15]	0	0	0	0	High	NA	NA	0	-0.03 [-0.21, 0.15]	1	High	0	-1	Moderate
Enhanced control:Game(2D)	0	NA	NA	NA	NA	NA	NA	1.92 [1.15, 2.69]	Moderate	0	1.92 [1.15, 2.69]	0	Moderate	0	-1	Low
Enhanced control:Midazolam	0	NA	NA	NA	NA	NA	NA	1.79 [0.94, 2.65]	Moderate	0	1.79 [0.94, 2.65]	0	Moderate	0	-1	Low
Enhanced control:Video(2D)	1	0.55 [0.09, 1]	0	0	0	0	High	NA	NA	0	0.55 [0.09, 1]	1	High	0	0	High
Enhanced control:Virtual reality	0	NA	NA	NA	NA	NA	NA	0.37 [-0.22, 0.95]	Moderate	0	0.37 [-0.22, 0.95]	0	Moderate	0	-1	Low
Game(2D):Midazolam	1	-0.13 [-0.49, 0.24]	0	0	0	0	High	NA	NA	0	-0.13 [-0.49, 0.24]	1	High	0	-1	Moderate
Game(2D):Video(2D)	0	NA	NA	NA	NA	NA	NA	-1.38 [-2, -0.75]	Moderate	0	-1.38 [-2, -0.75]	0	Moderate	0	0	Moderate
Game(2D):Virtual reality	0	NA	NA	NA	NA	NA	NA	-1.55 [-2.11, -0.99]	Moderate	0	-1.55 [-2.11, -0.99]	0	Moderate	0	0	Moderate
Midazolam:Video(2D)	0	NA	NA	NA	NA	NA	NA	-1.25 [-1.97, -0.53]	Moderate	0	-1.25 [-1.97, -0.53]	0	Moderate	0	0	Moderate
Midazolam:Virtual reality	0	NA	NA	NA	NA	NA	NA	-1.43 [-2.1, -0.76]	Moderate	0	-1.43 [-2.1, -0.76]	0	Moderate	0	0	Moderate
Video(2D):Virtual reality	0	NA	NA	NA	NA	NA	NA	-0.18 [-0.55,	Moderate	0	-0.18 [-0.55,	0	Moderate	0	-1	Low

© 2025 Luo Z et al. *JAMA Pediatrics*.

Comparisons	k	Direct	D1	D2	D3	D4	Direct Certainty	Indirect estimates	Initial Indirect Certainty	D5	NMA estimates	Prop	Initial NMA Certainty	D6	D7	NMA Certainty
								0.2]			0.2]					
**Parents - postoperative satisfaction**																
Comparisons	k	Direct	D1	D2	D3	D4	Direct Certainty	Indirect estimates	Initial Indirect Certainty	D5	NMA estimates	Prop	Initial NMA Certainty	D6	D7	NMA Certainty
Enhanced control:Control	0	NA [NA, NA]	NA	NA	NA	NA	NA	0.32 [-0.47, 1.11]	Moderate	0	0.32 [-0.47, 1.11]	0	Moderate	0	-1	Low
Game(2D):Control	1	-0.31 [-0.97, 0.36]	0	0	0	0	High	NA [NA, NA]	NA	0	-0.31 [-0.97, 0.36]	1	High	0	-1	Moderate
Midazolam:Control	0	NA [NA, NA]	NA	NA	NA	NA	NA	-0.6 [-1.41, 0.21]	Moderate	0	-0.6 [-1.41, 0.21]	0	Moderate	0	-1	Low
Video(2D):Control	1	0.56 [-0.15, 1.26]	0	0	0	0	High	0.29 [-0.47, 1.05]	Moderate	0	0.43 [-0.08, 0.95]	0.54	High	0	-1	Moderate
Virtual reality:Control	4	0.29 [-0.06, 0.64]	0	0	-1	0	Moderate	0.56 [-0.42, 1.53]	Moderate	0	0.32 [-0.01, 0.65]	0.88	Moderate	0	-1	Low
Enhanced control:Game(2D)	0	NA [NA, NA]	NA	NA	NA	NA	NA	0.63 [-0.41, 1.66]	Moderate	0	0.63 [-0.41, 1.66]	0	Moderate	0	-1	Low
Enhanced control:Midazolam	0	NA [NA, NA]	NA	NA	NA	NA	NA	0.92 [-0.21, 2.06]	Moderate	0	0.92 [-0.21, 2.06]	0	Moderate	0	-1	Low
Enhanced control:Video(2D)	0	NA [NA, NA]	NA	NA	NA	NA	NA	-0.11 [-1, 0.77]	Low	0	-0.11 [-1, 0.77]	0	Low	0	-1	Very low
Enhanced control:Virtual reality	1	0 [-0.72, 0.72]	0	0	0	0	High	NA [NA, NA]	NA	0	0 [-0.72, 0.72]	1	High	0	-1	Moderate
Game(2D):Midazolam	2	0.3 [-0.17, 0.77]	0	0	0	0	High	NA [NA, NA]	NA	0	0.3 [-0.17, 0.77]	1	High	0	-1	Moderate

© 2025 Luo Z et al. *JAMA Pediatrics*.

Game(2D):Video(2D)	0	NA [NA, NA]	NA	NA	NA	NA	NA	-0.74 [-1.58, 0.1]	Moderate	0	-0.74 [-1.58, 0.1]	0	Moderate	0	0	Moderate
Game(2D):Virtual reality	0	NA [NA, NA]	NA	NA	NA	NA	NA	-0.63 [-1.37, 0.11]	Moderate	0	-0.63 [-1.37, 0.11]	0	Moderate	0	-1	Low
Midazolam:Video(2D)	0	NA [NA, NA]	NA	NA	NA	NA	NA	-1.04 [-2, -0.07]	Moderate	0	-1.04 [-2, -0.07]	0	Moderate	0	0	Moderate
Midazolam:Virtual reality	0	NA [NA, NA]	NA	NA	NA	NA	NA	-0.92 [-1.8, -0.04]	Moderate	0	-0.92 [-1.8, -0.04]	0	Moderate	0	0	Moderate
Video(2D):Virtual reality	1	0 [-0.67, 0.67]	-1	0	0	0	Moderate	0.27 [-0.52, 1.06]	Moderate	0	0.11 [-0.4, 0.63]	0.58	Moderate	0	-1	Low

© 2025 Luo Z et al. *JAMA Pediatrics*.

eTable 10. Estimated effect size results for parents.

Outcome	SMD or MD (95% CI)	SMD Reexpressed on a Common Scale, MD (95% CI)[a]	P score	Certainty of Evidence
**Parents - preoperative anxiety**				
Game(2D)	SMD -1.59 (-2.12,-1.06)	The State-Trait Anxiety Inventory (20-80)<br>MD -11.00 (-14.67, -7.34)	0.95	⊕⊕⊕⊖Moderate[b]
Midazolam	SMD -1.46 (-2.10,-0.82)	The State-Trait Anxiety Inventory (20-80)<br>MD -10.10 (-14.53, -5.67)	0.85	⊕⊕⊕⊖Moderate[c]
Video(2D)	SMD -0.21 (-0.54,0.12)	The State-Trait Anxiety Inventory (20-80)<br>MD -1.45 (-3.74, 0.83)	0.54	⊕⊕⊕⊖Moderate[d]
Virtual reality	SMD -0.03 (-0.21,0.15)	The State-Trait Anxiety Inventory (20-80)<br>MD -0.21 (-1.45, 1.04)	0.34	⊕⊕⊕⊖Moderate[d]
Control	-	-	0.27	-
Enhanced control	SMD 0.17 (-0.48, 0.82)	The State-Trait Anxiety Inventory (20-80)<br>MD 1.18 (-3.32, 5.67)	0.05	⊕⊕⊖⊖Low[c,d]
**Parents - postoperative satisfaction**				
Video(2D)	SMD 0.43 (-0.08; 0.95)	Verbal Score (0-10)<br>MD 0.52 (−0.10, 1.15)	0.83	⊕⊕⊕⊖Moderate[d]
Virtual reality	SMD 0.32 (-0.01; 0.65)	Verbal Score (0-10)<br>MD 0.39 (−0.01, 0.79)	0.75	⊕⊕⊖⊖Low[d,e]
Enhanced control	SMD 0.32 (-0.47; 1.11)	Verbal Score (0-10)<br>MD 0.39 (−0.57, 1.34)	0.70	⊕⊕⊖⊖Low[c,d]
Control	-	-	0.41	-
Game(2D)	SMD -0.31 (-0.97; 0.36)	Verbal Score (0-10)<br>MD −0.38 (−1.17, 0.44)	0.26	⊕⊕⊕⊖Moderate[d]
Midazolam	SMD -0.60 (-1.41; 0.21)	Verbal Score (0-10)	0.05	⊕⊕⊖⊖Low[c,d]

© 2025 Luo Z et al. *JAMA Pediatrics*.

MD −0.73 (−1.71, 0.25)

Abbreviations: SMD: Standardized mean difference; CI: confidence interval; GRADE: The Grading of Recommendations Assessment, Development, and Evaluation

[a] MDs should be interpreted with caution because the results are based on the weighted control group SD for a subset of studies in the analysis that used the most commonly reported scale.

[b] Downgraded by 1 level due to concerns about methodological considerations, including a lack of blinding and, in some cases, other sources of bias.

[c] Downgraded by 1 level due to concerns about the indirect certainty being derived from other direct comparison evidence, which is of moderate quality.

[d] Downgraded by 1 level due to concerns about imprecision; the confidence interval suggests the possibility of a null effect or benefit for either intervention.

[e] Downgraded by 1 level due to concerns about heterogeneity of effect estimates across trials.

© 2025 Luo Z et al. *JAMA Pediatrics*.

eFigure 1. Assessment of the risk of bias in all included studies. (A) Graph of Risk of Bias; (B) Summary of Risk of Bias.

A

tudy ID						
Buffel et al (2019)	-	?	+	+	+	-
Kerimoglu et al (2013)	+	+	+	+	+	+
Luo et al (2023)	+	?	+	+	+	?
Marechal et al (2017)	+	+	+	+	+	+
Patel et al (2006)	?	?	+	?	+	?
Hou et al (2023)	+	?	+	+	+	?
Baghele et al (2019)	+	?	+	+	+	?
Kerimaa et al (2023)	+	+	+	+	+	+
Liguori et al (2016)	+	+	+	+	+	+
Härter et al (2021)	?	-	+	?	+	-
Nair et al (2021)	+	+	+	+	+	+
Tang et al (2023)	+	?	+	+	+	?
Dost et al (2023)	+	?	+	+	+	?
Huntington et al (2017)	+	?	+	+	+	?
Jung et al (2021)	+	+	+	+	+	+
Kumar et al (2019)	-	?	+	?	+	-
Wang et al (2021)	?	?	+	?	+	?
Ryu et al (2017)	+	+	+	+	+	+
Stewart et al (2019)	?	?	+	+	?	?
Cumino et al (2017)	?	?	+	?	+	?
Mifflin et al (2012)	+	+	+	+	+	+
Seiden et al (2014)	+	?	+	-	+	-
Levay et al (2023)	?	-	+	+	+	-
Topçu et al (2023)	?	+	+	?	+	?
Ryu et al (2018)	?	?	+	+	+	?
Tuncay et al (2023)	?	?	+	+	+	?
Evren Sahin et al (2022)	-	-	+	+	+	-
Buyuk et al (2021)	+	?	?	+	?	?
Uysal et al (2023)	?	+	+	?	+	?
Yaz et al (2022)	?	?	+	?	?	!
Huang et al (2021)	+	?	+	?	+	!
Ryu et al (2022)	?	+	+	+	+	?
Clausen et al (2021)	?	?	+	?	+	?
Eijlers et al (2019)	+	+	+	+	+	+
Wu et al (2022)	?	-	?	+	+	-
Lee et al (2014)	?	?	+	+	+	?
Carbó et al (2024)	+	?	?	?	+	?
Mihandoust et al (2024)	-	!	-	+	+	-
Bozkul et al (2024)	?	?	?	+	+	?
Franco Castanys et al (2023)	?	?	?	+	+	?
Chartrand et al (2016)	+	+	?	+	+	?
Ryu et al (2018)	+	+	+	+	?	?
Mete İzci et al (2024)	+	?	+	?	+	?
Specht et al (2023)	+	?	-	?	+	-
Yang et al (2016)	+	?	?	?	+	?
Chen et al (2025)	+	?	+	+	+	?
Turgut et al (2024)	+	?	+	?	+	?
Carbó et al (2024)	+	?	?	?	+	?
Vinay et al (2024)	+	?	+	?	+	?

\+ Low risk
? Some concerns
\- High risk

D1 Randomisation process
D2 Deviations from the intended interventions
D3 Missing outcome data
D4 Measurement of the outcome
D5 Selection of the reported result

© 2025 Luo Z et al. *JAMA Pediatrics*.

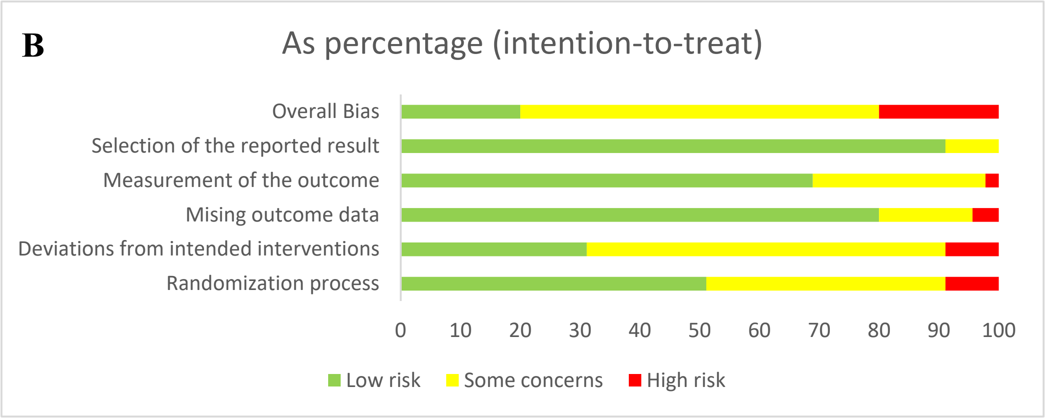

© 2025 Luo Z et al. *JAMA Pediatrics*.

eFigure 2. Comparison-adjusted funnel plot in relation to the network meta-analysis of primary outcomes. (A) Children - Preoperative Anxiety, (B) Children - Postoperative Pain, (C) Children - Emergence Delirium, (D) Parent - Preoperative Anxiety, and (E) Parent - Postoperative Satisfaction. The red line indicates the null hypothesis that the comparison-specific pooled effect estimates do not differ from the respective study-specific effect sizes.

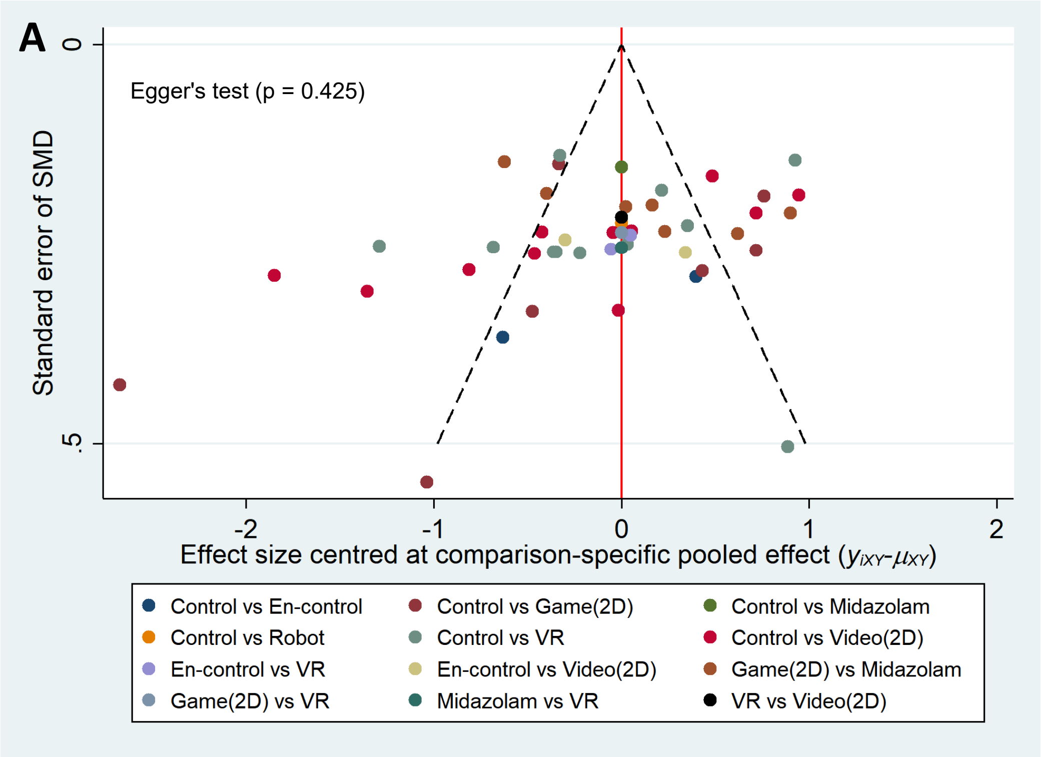

© 2025 Luo Z et al. *JAMA Pediatrics*.

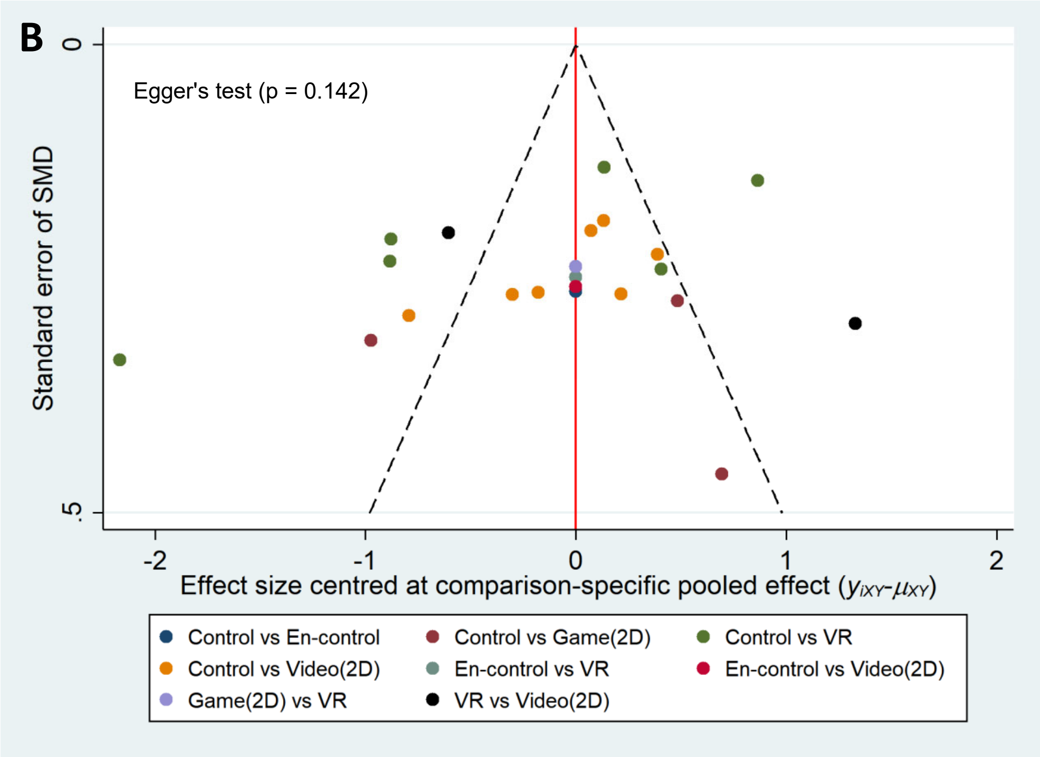

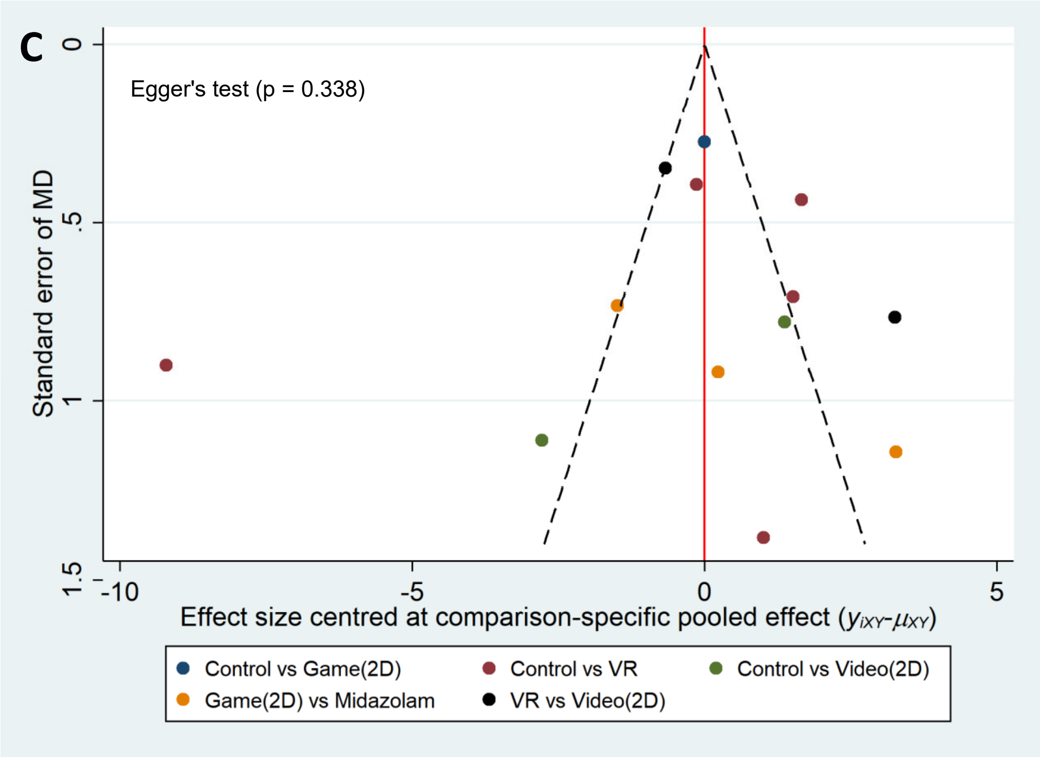

© 2025 Luo Z et al. *JAMA Pediatrics*.

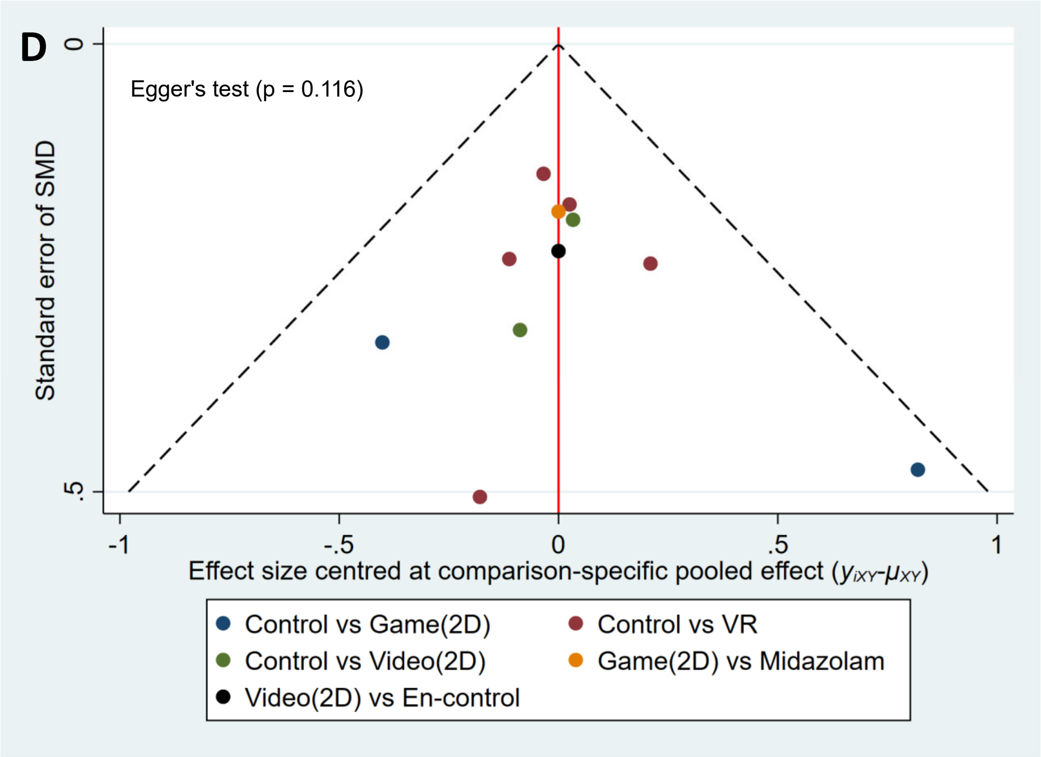

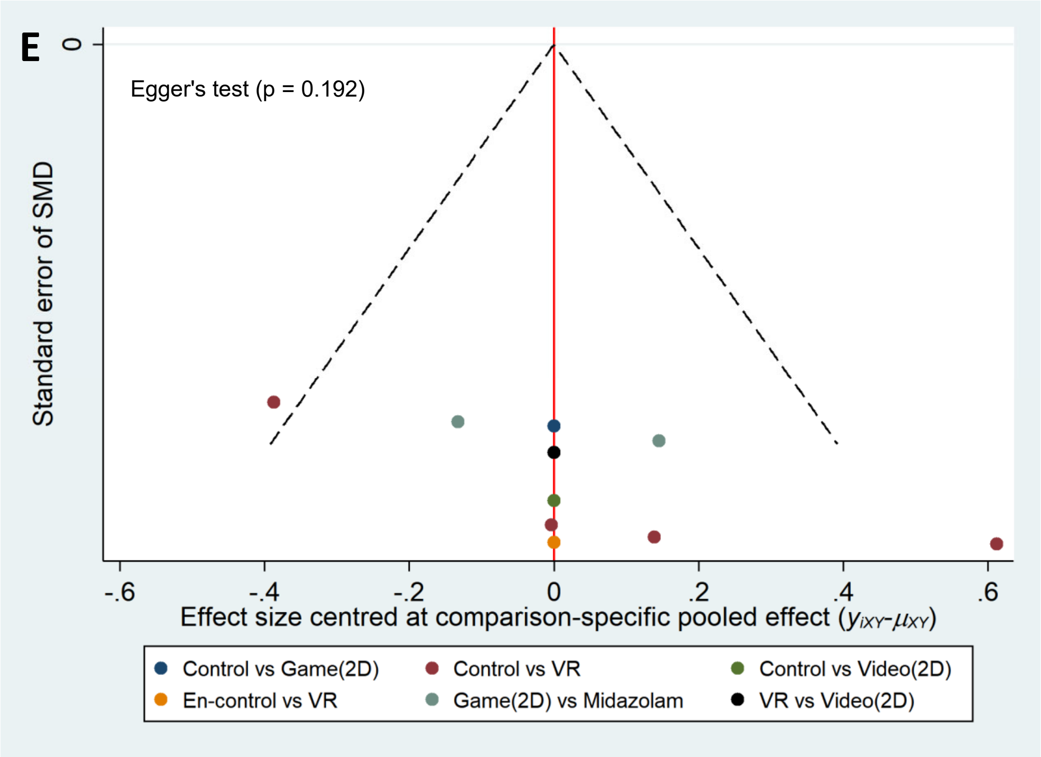

© 2025 Luo Z et al. *JAMA Pediatrics*.

eFigure 3. Forest plots

(A) Children - preoperative anxiety

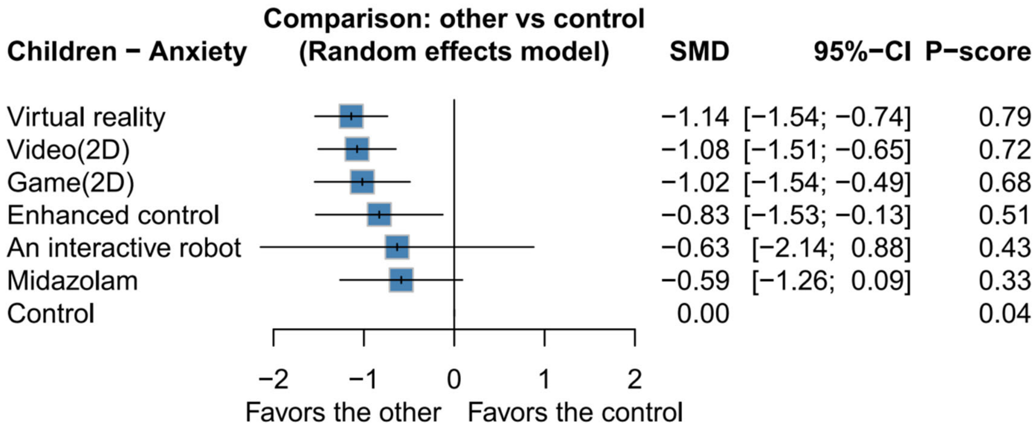

(B) Children - postoperative pain

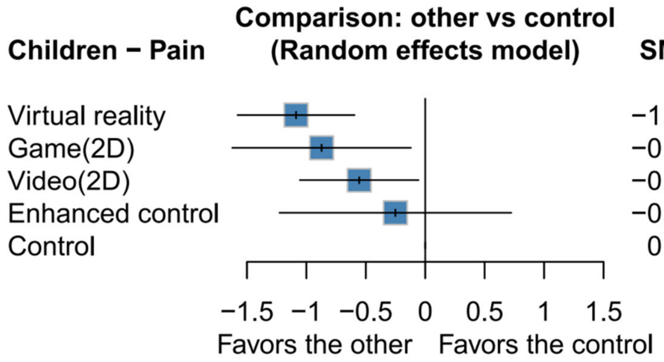

© 2025 Luo Z et al. *JAMA Pediatrics*.

(C) Children - emergence delirium

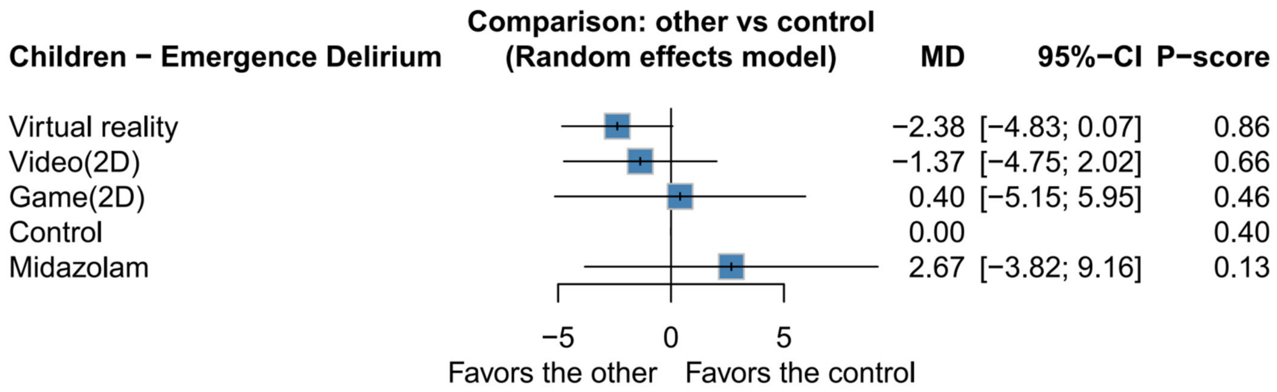

(D) Children - induction compliance

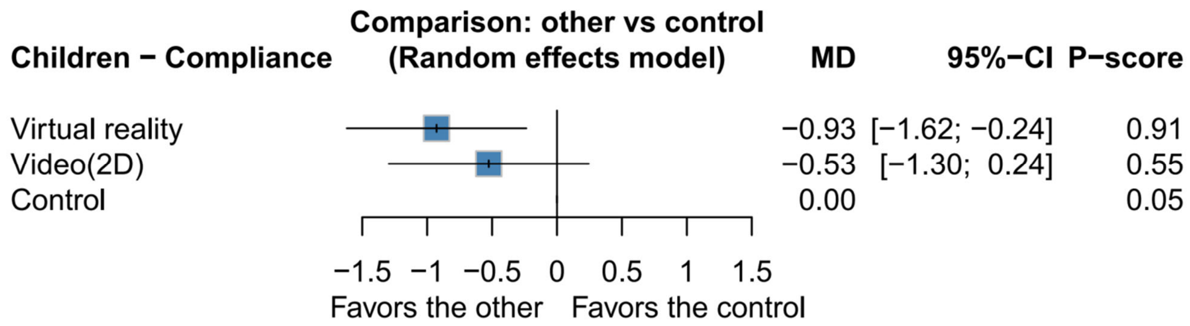

© 2025 Luo Z et al. *JAMA Pediatrics*.

(E) Parents - preoperative anxiety

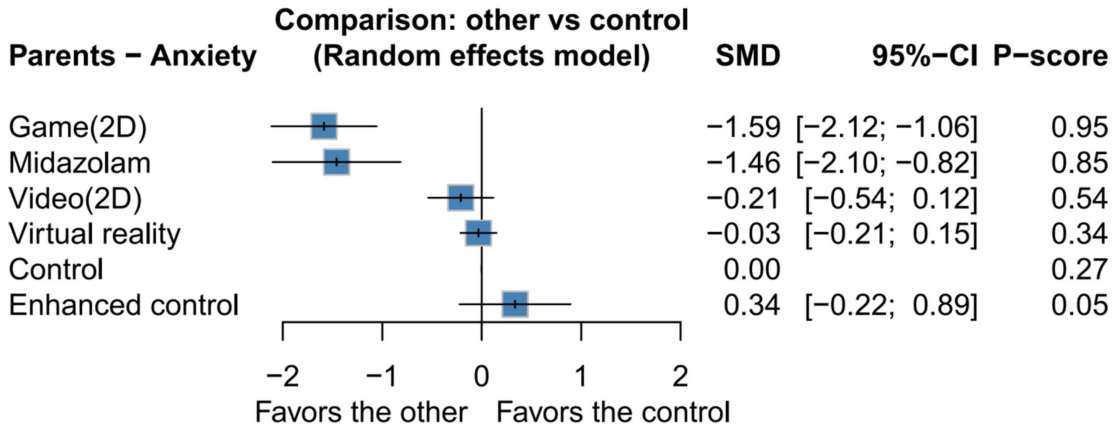

(F) Parents - postoperative satisfaction

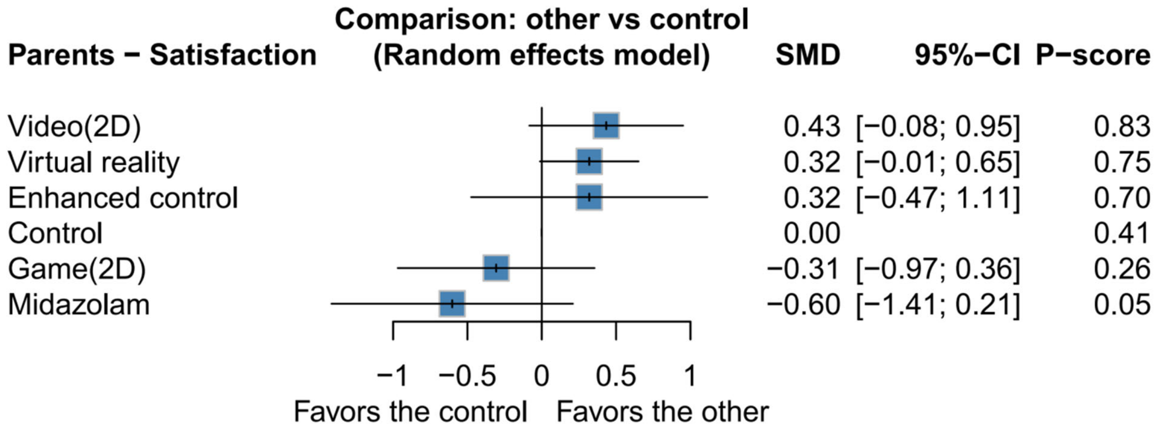

© 2025 Luo Z et al. *JAMA Pediatrics*.

eFigure 4. Radar Plots of Interventions Based on P-Scores for Each Outcome. (A) Virtual Reality, (B) Video (2D), (C) Game (2D), (D) Enhanced Control, (E) Midazolam, and (F) Control. The six axes of the radar plot represent different outcomes. Each point on the radar plot indicates the ranking probability, with points closer to the outer edge representing a higher ranking probability.

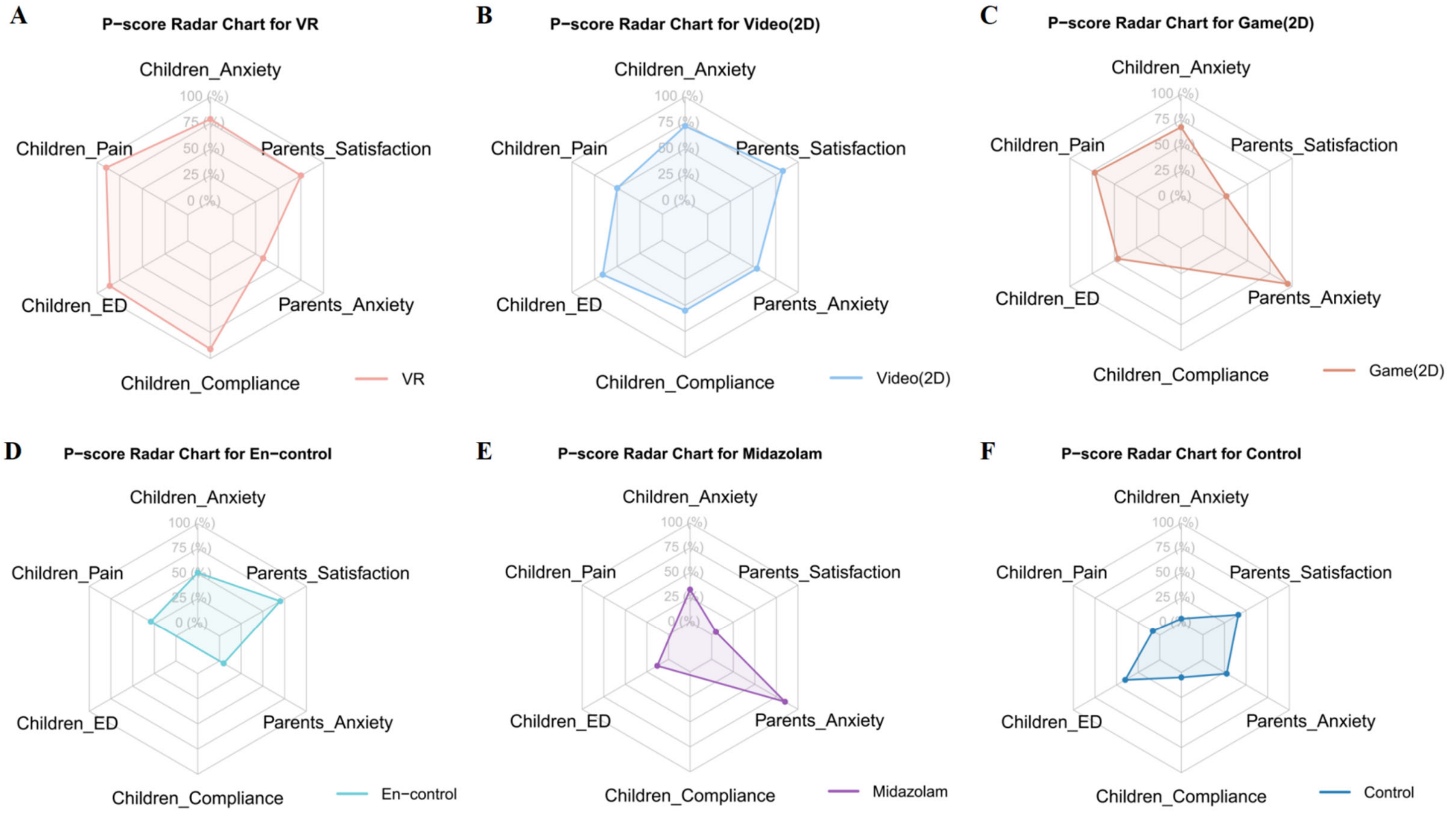

© 2025 Luo Z et al. *JAMA Pediatrics*.

eFigure 5. Network Plots of Digital Health Interventions for Secondary Outcomes. (A) Parent Preoperative Anxiety, (B) Parent Postoperative Satisfaction. Each circular node represents a type of intervention. The circle size is proportional to the total number of patients. Connecting lines indicate direct comparisons of interventions, and their width is proportional to the number of pairwise comparisons.

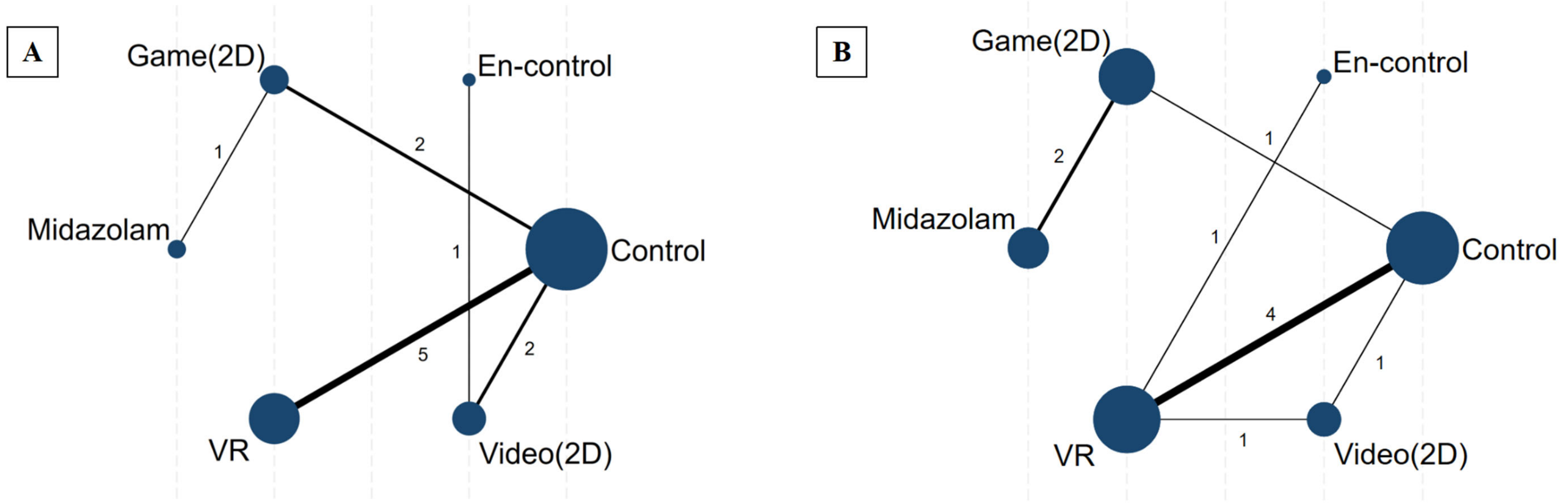

© 2025 Luo Z et al. *JAMA Pediatrics*.

eFigure 6. League Tables of Parental Preoperative Anxiety and Postoperative Satisfaction. The league tables show the absolute effects of each intervention and usual care (the column's treatment vs. the row's treatment). The absolute effects are measured as a standardized mean difference for outcomes along with 95% confidence intervals. Bold indicates statistical significance. The colour of each cell indicates the certainty of evidence according to the Grading of Recommendations, Assessment, Development, and Evaluations (GRADE).

High **Moderate** **Low** **Very Low**

Parents - Preoperative Anxiety (SMD, 95%CI)

**Virtual reality**					
**1.43 (0.76; 2.10)**	**Midazolam**				
**1.55 (0.99; 2.11)**	0.13 (-0.24; 0.49)	**Game (2D)**			
0.18 (-0.20; 0.55)	**-1.25 (-1.97; -0.53)**	**-1.38 (-2.00; -0.75)**	**Video (2D)**		
-0.03 (-0.21; 0.15)	**-1.46 (-2.10; -0.82)**	**-1.59 (-2.12; -1.06)**	-0.21 (-0.54; 0.12)	**Control**	
-0.37 (-0.95; 0.22)	**-1.79 (-2.65; -0.94)**	**-1.92 (-2.69; -1.15)**	**-0.55 (-1.00; -0.09)**	-0.34 (-0.89; 0.22)	**Enhanced control**

Parents - Postoperative Satisfaction (SMD, 95%CI)

**Virtual reality**					
**0.92 (0.04; 1.80)**	**Midazolam**				
0.63 (-0.11; 1.37)	-0.30 (-0.77; 0.17)	**Game (2D)**			
-0.11 (-0.63; 0.40)	**-1.04 (-2.00; -0.07)**	-0.74 (-1.58; 0.10)	**Video (2D)**		
0.32 (-0.01; 0.65)	-0.60 (-1.41; 0.21)	-0.31 (-0.97; 0.36)	0.43 (-0.08; 0.95)	**Control**	
0.00 (-0.72; 0.72)	-0.92 (-2.06; 0.21)	-0.63 (-1.66; 0.41)	0.11 (-0.77; 1.00)	-0.32 (-1.11; 0.47)	**Enhanced control**

© 2025 Luo Z et al. *JAMA Pediatrics*.

eFigure 7. Subgroup Analysis: Network Meta-Analysis of Anxiety in Children Undergoing Day or Outpatient Surgery. (A) Network plot of included studies evaluating digital health interventions. (B) Forest plot comparing the effect of each digital health intervention to the control. (C) League table presenting a summary of all pairwise comparisons among digital health interventions. Bold text represents statistical significance.

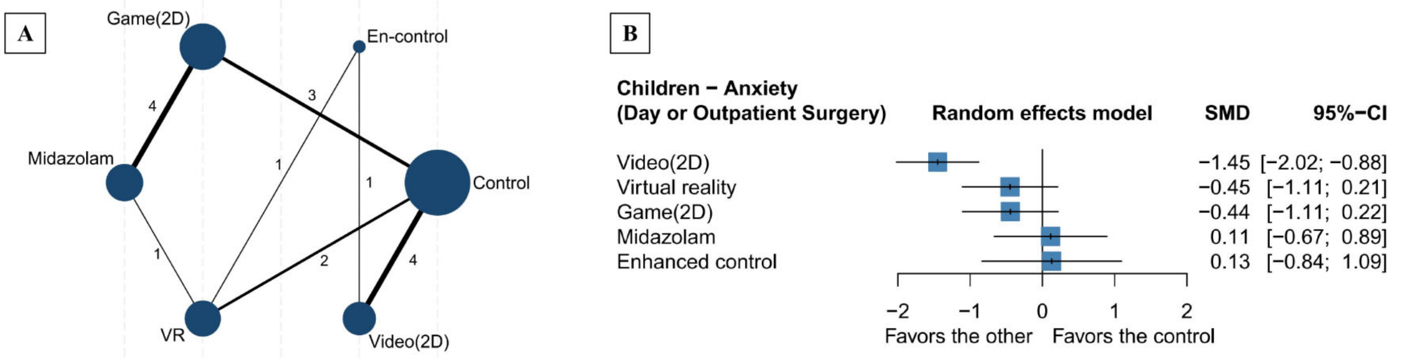

C

**Virtual reality**					
-0.56 (-1.41; 0.28)	**Midazolam**				
-0.00 (-0.83; 0.82)	**0.56 (0.01; 1.11)**	**Game (2D)**			
**1.00 (0.18; 1.82)**	**1.56 (0.62; 2.51)**	**1.01 (0.14; 1.87)**	**Video (2D)**		
-0.45 (-1.11; 0.21)	0.11 (-0.67; 0.89)	-0.44 (-1.11; 0.22)	**-1.45 (-2.02; -0.88)**	**Control**	
-0.58 (-1.52; 0.37)	-0.01 (-1.18; 1.15)	-0.57 (-1.69; 0.55)	**-1.58 (-2.52; -0.64)**	-0.13 (-1.09; 0.84)	**Enhanced control**

Children - Anxiety (Day or Outpatient Surgery)

© 2025 Luo Z et al. *JAMA Pediatrics*.

eFigure 8. Subgroup Analysis: Network Meta-Analysis of Anxiety in Children Undergoing Elective Surgery. (A) Network plot of included studies evaluating digital health interventions. (B) Forest plot comparing the effect of each digital health intervention to the control. (C) League table presenting a summary of all pairwise comparisons among digital health interventions. Bold text represents statistical significance.

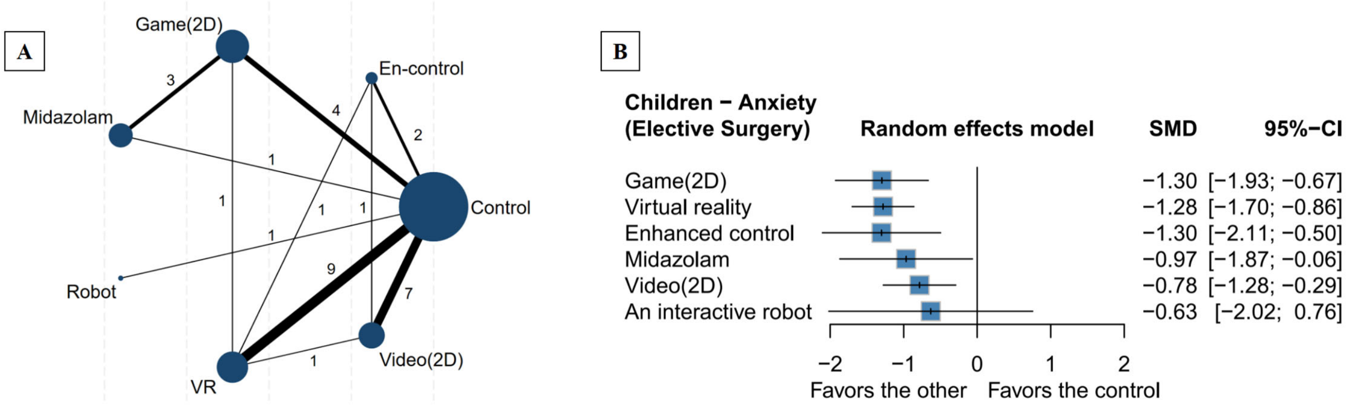

C

**Virtual reality**						
-0.31 (-1.28; 0.65)	**Midazolam**					
0.02 (-0.69; 0.73)	0.33 (-0.43; 1.09)	**Game (2D)**				
-0.50 (-1.10; 0.11)	-0.18 (-1.21; 0.84)	-0.51 (-1.31; 0.28)	**Video (2D)**			
**-1.28 (-1.70; -0.86)**	**-0.97 (-1.87; -0.06)**	**-1.30 (-1.93; -0.67)**	**-0.78 (-1.28; -0.29)**	**Control**		
0.02 (-0.82; 0.86)	0.33 (-0.87; 1.54)	0.00 (-1.01; 1.02)	0.52 (-0.36; 1.39)	**1.30 (0.50; 2.11)**	**Enhanced control**	
-0.65 (-2.10; 0.80)	-0.33 (-1.99; 1.32)	-0.66 (-2.19; 0.86)	-0.15 (-1.63; 1.32)	0.63 (-0.76; 2.02)	-0.67 (-2.27; 0.93)	**Robot**

Children - Anxiety (Elective Surgery)

© 2025 Luo Z et al. *JAMA Pediatrics*.

eFigure 9. Subgroup Analysis: P-Score Comparison of Digital Health Interventions for Reducing Children's Anxiety Across Surgery Types (Day/Outpatient vs. Elective Surgery).

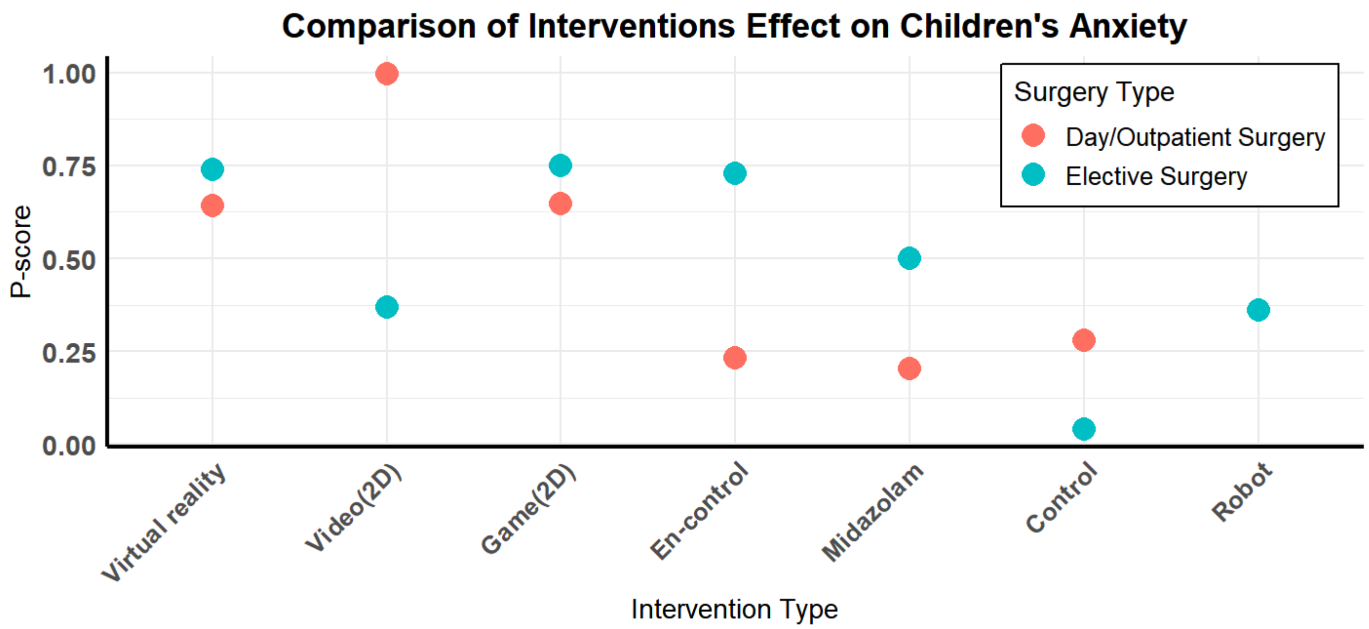

© 2025 Luo Z et al. *JAMA Pediatrics*.

eFigure 10. Subgroup Analysis: Network Meta-Analysis of Pain in Children Undergoing Day or Outpatient Surgery. (A) Network plot of included studies evaluating digital health interventions. (B) Forest plot comparing the effect of each digital health intervention to the control. (C) League table presenting a summary of all pairwise comparisons among digital health interventions. Bold text represents statistical significance.

A

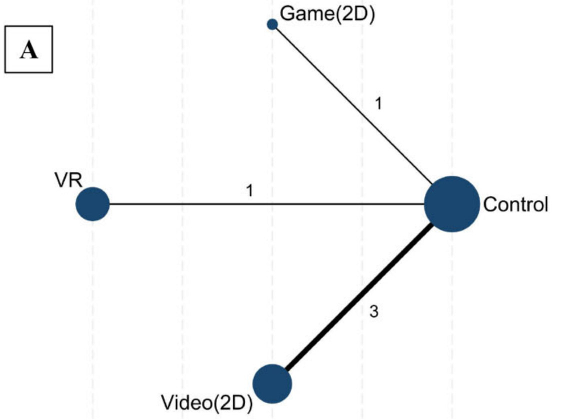

B

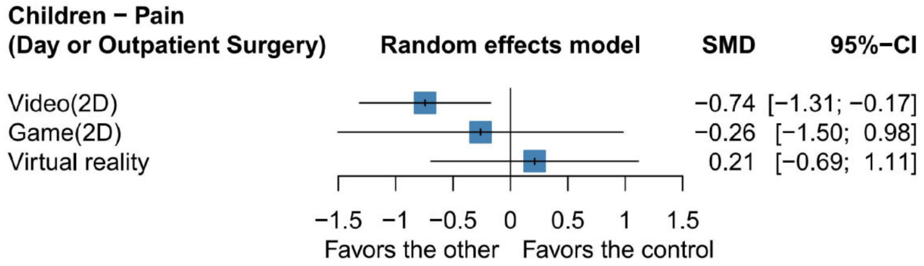

C

**Virtual reality**			
0.47 (-1.06; 2.01)	**Game (2D)**		
0.95 (-0.11; 2.02)	0.48 (-0.88; 1.85)	**Video (2D)**	
0.21 (-0.69; 1.11)	-0.26 (-1.50; 0.98)	**-0.74 (-1.31; -0.17)**	**Control**

Children - Pain (Day or Outpatient Surgery)

© 2025 Luo Z et al. *JAMA Pediatrics*.

eFigure 11. Subgroup Analysis: Network Meta-Analysis of Pain in Children Undergoing Elective Surgery. (A) Network plot of included studies evaluating digital health interventions. (B) Forest plot comparing the effect of each digital health intervention to the control. (C) League table presenting a summary of all pairwise comparisons among digital health interventions. Bold text represents statistical significance.

A

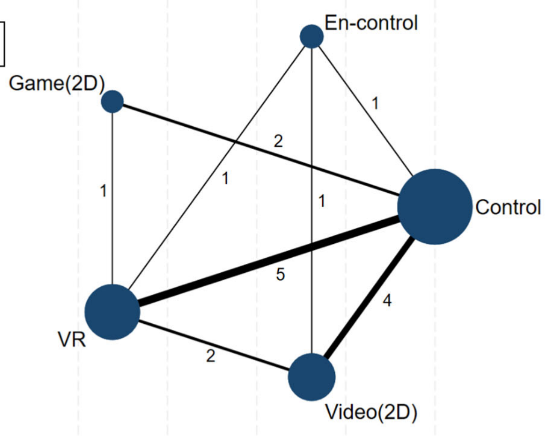

B

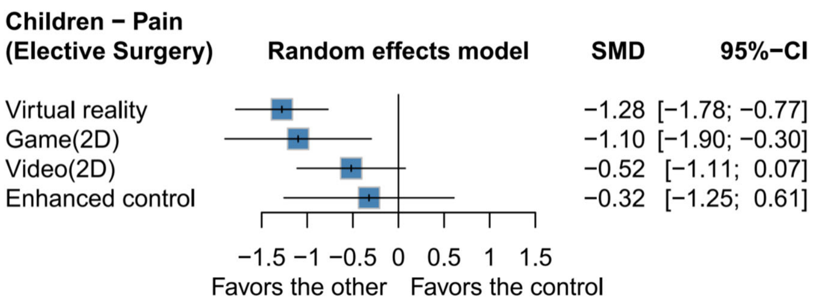

C

**Virtual reality**				
-0.18 (-1.03; 0.67)	**Game (2D)**			
**-0.76 (-1.41; -0.11)**	-0.58 (-1.55; 0.38)	**Video (2D)**		
**-1.28 (-1.78; -0.77)**	**-1.10 (-1.90; -0.30)**	-0.52 (-1.11; 0.07)	**Control**	
**-0.96 (-1.88; -0.03)**	-0.78 (-1.97; 0.41)	-0.20 (-1.17; 0.77)	0.32 (-0.61; 1.25)	**Enhanced control**

Children - Pain (Elective Surgery)

© 2025 Luo Z et al. *JAMA Pediatrics*.

eFigure 12. Subgroup Analysis: P-Score Comparison of Digital Health Interventions for Reducing Children's Pain Across Surgery Types (Day/Outpatient vs. Elective Surgery).

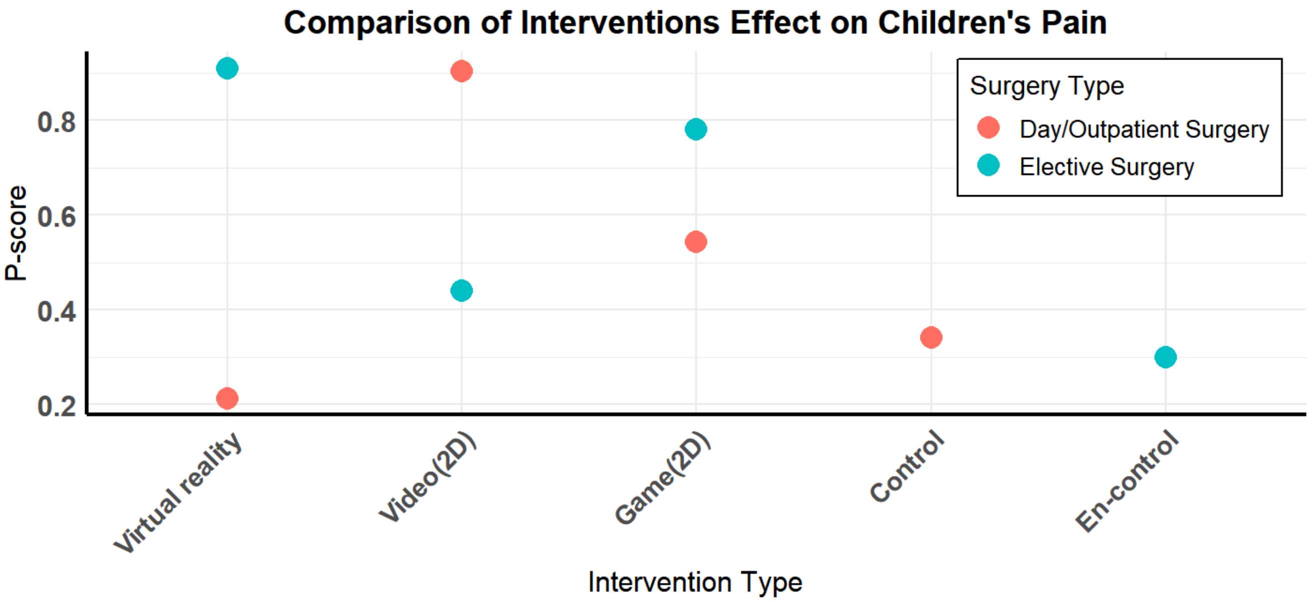

© 2025 Luo Z et al. *JAMA Pediatrics*.

eFigure 13. Sensitivity analysis: Exclusion of Studies with High Risk of Bias.

A. Sensitivity analysis for Anxiety in Children

Forest plot

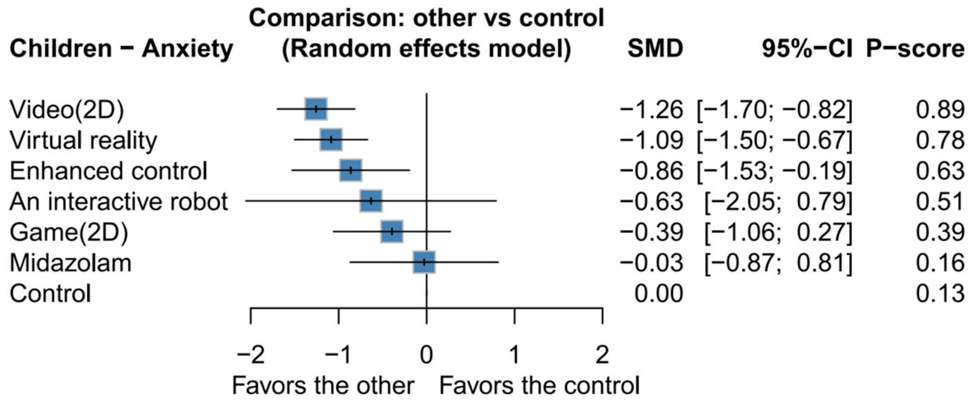

(I^2 = 90.1% (87.0%–92.5%); P < .001 at Q test)

League table

Children - Anxiety (SMD,95%CI)

**Virtual reality**						
**-1.06 (-1.93; -0.18)**	**Midazolam**					
-0.69 (-1.44; 0.05)	0.37 (-0.29; 1.02)	**Game (2D)**				
0.17 (-0.42; 0.76)	1.23 (0.28; 2.18)	**0.86 (0.07; 1.66)**	**Video (2D)**			
**-1.09 (-1.50; -0.67)**	-0.03 (-0.87; 0.81)	-0.39 (-1.06; 0.27)	**-1.26 (-1.70; -0.82)**	**Control**		
-0.22 (-0.92; 0.47)	0.83 (-0.22; 1.89)	0.47 (-0.46; 1.40)	-0.39 (-1.11; 0.32)	**0.86 (0.19; 1.53)**	**Enhanced control**	
-0.45 (-1.94; 1.03)	0.60 (-1.05; 2.26)	0.24 (-1.33; 1.81)	-0.63 (-2.11; 0.86)	0.63 (-0.79; 2.05)	-0.23 (-1.80; 1.34)	**Robot**

© 2025 Luo Z et al. *JAMA Pediatrics*.

B. Sensitivity analysis for Pain in Children

Forest plot

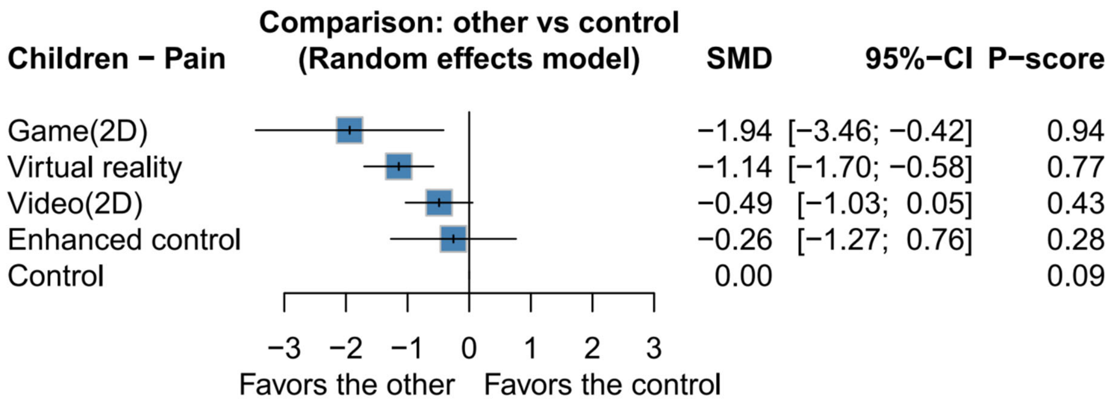

(I^2 = 91.6% (87.5%–94.4%); P < .001 at Q test)

League table

Children - Pain (SMD,95%CI)

**Virtual reality**				
0.80 (-0.83; 2.42)	**Game (2D)**			
-0.65 (-1.38; 0.08)	-1.45 (-3.07; 0.17)	**Video (2D)**		
**-1.14 (-1.70; -0.58)**	**-1.94 (-3.46; -0.42)**	-0.49 (-1.03; 0.05)	**Control**	
-0.89 (-1.91; 0.14)	-1.68 (-3.52; 0.15)	-0.23 (-1.29; 0.83)	0.26 (-0.76; 1.27)	**Enhanced control**

© 2025 Luo Z et al. *JAMA Pediatrics*.

C. Sensitivity analysis for Emergence Delirium in Children

Forest plot

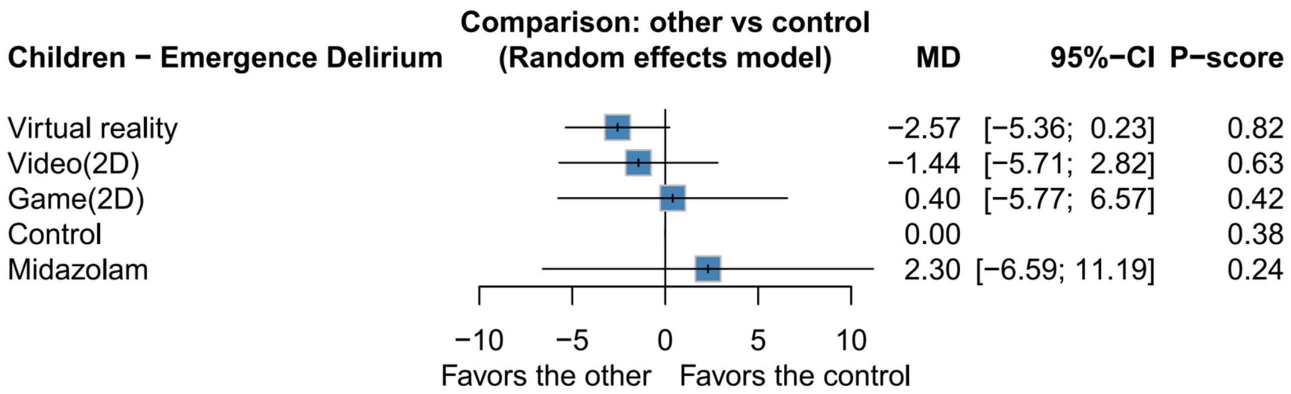

(I^2 = 96% (93.5%–97.6%); $P < .001$ at Q test)

League table

Children - Emergence Delirium (MD,95%CI)

**Virtual reality**				
-4.87 (-14.18; 4.45)	**Midazolam**			
-2.97 (-9.74; 3.80)	1.90 (-4.50; 8.30)	**Game (2D)**		
-1.12 (-5.74; 3.49)	3.74 (-6.11; 13.60)	1.84 (-5.65; 9.34)	**Video (2D)**	
-2.57 (-5.36; 0.23)	2.30 (-6.59; 11.19)	0.40 (-5.77; 6.57)	-1.44 (-5.71; 2.82)	**Control**

© 2025 Luo Z et al. *JAMA Pediatrics*.

D. Sensitivity analysis for Compliance in Children

Forest plot

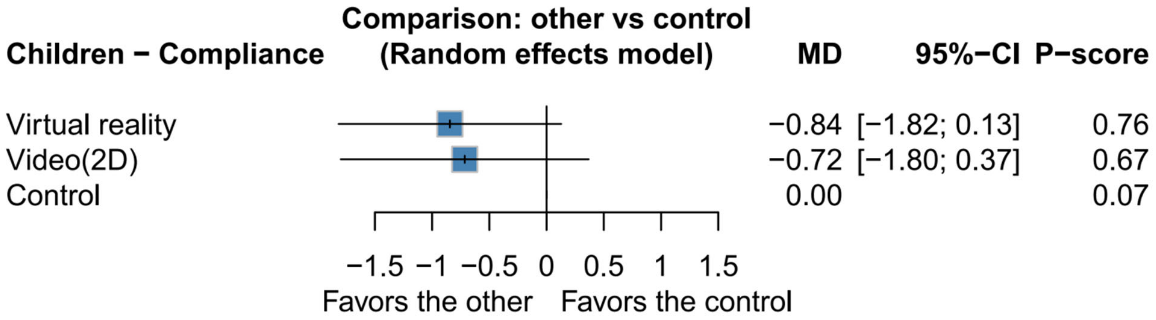

($I^2 = 90.6\%$ (81.0%–95.3%); $P < .001$ at Q test)

League table

Children - Compliance (MD,95%CI)

**Virtual reality**		
-0.13 (-1.58; 1.33)	**Video (2D)**	
-0.84 (-1.82; 0.13)	-0.72 (-1.80; 0.37)	**Control**

© 2025 Luo Z et al. *JAMA Pediatrics*.

E. Sensitivity analysis for Anxiety in Parents

Forest plot

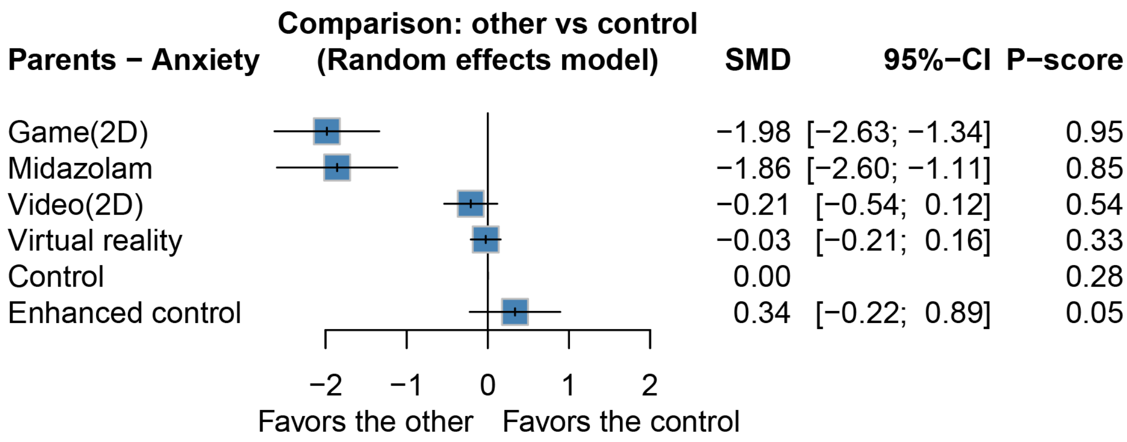

(I^2= 0% (0.0%–79.2%); P=0.8850 at Q test)

League table

Parents - Anxiety (SMD,95%CI)

**Virtual reality**					
**1.83 (1.07; 2.60)**	**Midazolam**				
**1.96 (1.29; 2.63)**	0.13 (-0.24; 0.49)	**Game (2D)**			
0.18 (-0.19; 0.56)	**-1.65 (-2.46; -0.84)**	**-1.77 (-2.50; -1.05)**	**Video (2D)**		
-0.03 (-0.21; 0.16)	**-1.86 (-2.60; -1.11)**	**-1.98 (-2.63; -1.34)**	-0.21 (-0.54; 0.12)	**Control**	
-0.36 (-0.95; 0.23)	**-2.19 (-3.12; -1.26)**	**-2.32 (-3.17; -1.47)**	**-0.55 (-1.00; -0.09)**	-0.34 (-0.89; 0.22)	**Enhanced control**

© 2025 Luo Z et al. *JAMA Pediatrics*.

F. Sensitivity analysis for Satisfaction in Parents

Forest plot

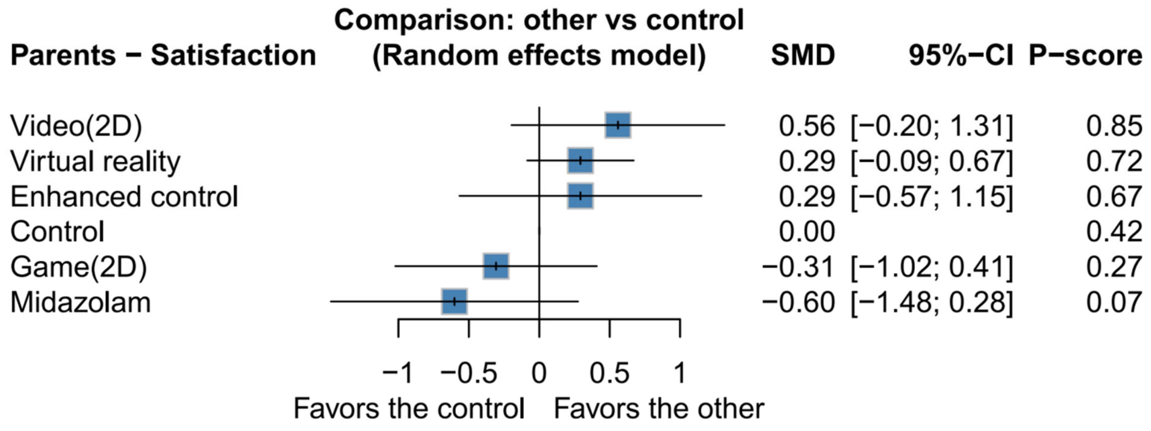

(I^2 = 67.4% (15.6%–87.4%); *P* = 0.015 at Q test)

League table

Parents - Satisfaction (SMD,95%CI)

**Virtual reality**					
0.89 (-0.06; 1.85)	**Midazolam**				
0.60 (-0.21; 1.41)	-0.30 (-0.80; 0.21)	**Game (2D)**			
-0.27 (-1.11; 0.58)	-1.16 (-2.32; 0.00)	-0.87 (-1.91; 0.18)	**Video (2D)**		
0.29 (-0.09; 0.67)	-0.60 (-1.48; 0.28)	-0.31 (-1.02; 0.41)	0.56 (-0.20; 1.31)	**Control**	
0.00 (-0.77; 0.77)	-0.89 (-2.12; 0.33)	-0.60 (-1.72; 0.52)	0.27 (-0.88; 1.41)	-0.29 (-1.15; 0.57)	**Enhanced control**

© 2025 Luo Z et al. *JAMA Pediatrics*.
