# Supplementary material for: Digital Health Interventions in Pediatric Perioperative Care: A Network Meta-Analysis
Source: JAMA Pediatr. 2025 Sep 15;179(11):1153–61. doi: 10.1001/jamapediatrics.2025.3099 (PMC12439185; doi:10.1001/jamapediatrics.2025.3099)
Supplement: Supplement 2. — Data sharing statement [file jamapediatr-e253099-s002.pdf]

Data Sharing Statement

Luo. Digital Health Interventions in Pediatric Perioperative Care. *JAMA Pediatr*. Published September 15, 2025. doi:10.1001/jamapediatrics.2025.3099

Data
Data available: No
